# Novel prenylated diketopiperazines with anti-osteoclastogenic activity from the deep-sea-derived *Penicillium limosum* ZEN48

**DOI:** 10.1007/s42995-026-00385-2

**Published:** 2026-05-19

**Authors:** Yong Zhang, Yan-Qing Guo, You Li, Zheng-Biao Zou, Zhe-Wen Chen, Rong Zhao, Robert J. Capon, Tai-Zong Wu, Chun-Lan Xie, Xian-Wen Yang

**Affiliations:** 1 Hainan Academy of Medical Sciences, Hainan Medical University, Haikou, 571199 China; 2 Key Laboratory of Tropical Translational Medicine of Ministry of Education, School of Basic Medicine and Life Sciences, Hainan Medical University, Haikou, 571199 China; 3 Key Laboratory of Marine Genetic Resources, Third Institute of Oceanography, Ministry of Natural Sources, Xiamen, 361005 China; 4 Institute for Molecular Bioscience, The University of Queensland, Brisbane, QLD 4072 Australia

**Keywords:** Deep-sea-derived fungus, *Penicillium limosum*, Diketopiperazines, Osteoclast differentiation, Ferroptosis, Anti-osteoporotic

## Abstract

A systematic chemical investigation of the deep-sea-derived fungus *Penicillium limosum* ZEN48 resulted in the isolation of four new indole-diketopiperazine alkaloids, limopiperazines A–D (**1**–**4**), alongside 16 known analogues (**5**–**20**). The structures of the new compounds were determined through comprehensive spectroscopic analysis, quantum chemical calculations, X-ray crystallography, and biogenetic considerations. Limopiperazine C (**3**) potently inhibited osteoclast differentiation and disrupted actin ring formation. Integrated RNA sequencing, RT-qPCR and molecular docking revealed that limopiperazine C exerts the anti-osteoclastogenic effect by modulating the ferroptosis signaling pathway via targeting heme oxygenase-1 (Hmox-1), positioning it as a promising lead compound for developing anti-osteoporotic agents.

**Supplementary Information:**

The online version contains supplementary material available at https://doi.org/10.1007/s42995-026-00385-2.

## Introduction

2,5-Diketopiperazines (2,5-DKPs) are a family of cyclodipeptides characterized by the double lactam core obtained by the “head to tail” condensation of two amino acids, primarily tryptophan and proline in natural products (Chi et al. 2025; Huang et al. 2014; Yu et al. 2017). The rigid backbone of 2,5-DKPs mimics stable peptide conformations and displays constrained amino acids while avoiding the poor physicochemical and metabolic properties of peptides, enabling them of a great value in drug discovery (Borthwick 2012). Naturally occurring 2,5-DKPs usually incorporate several isoprene units around the tryptophan moiety, thus generating a vast diversity of analogues (Wang et al. 2022). Those structurally diverse 2,5-DKPs exhibit comprehensive bioactivities, including antitumour (Kanoh et al. 1999), antibacterial (Wang et al. 2022), antifungus (Li et al. 2012), antihyperglycaemic (Song et al. 2003), analgesic (Canu et al. 2018) and neuroprotection (Faden et al. 2003).

**Fig. 1:**
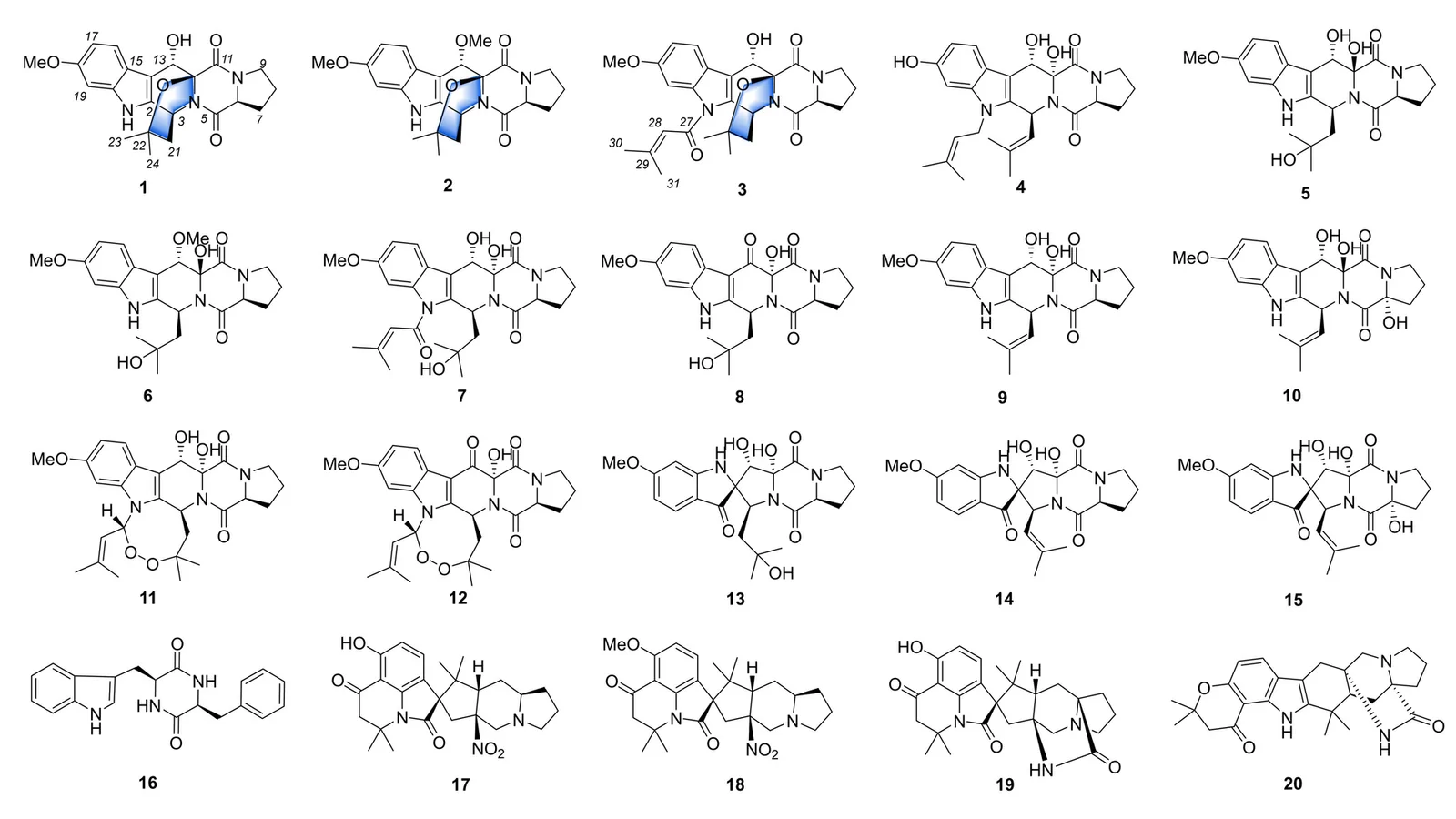
Chemical structures of compounds **1**–**20** from *Penicillium limosum* ZEN48

Osteoporosis, a prevalent bone disorder, is characterized by diminished bone density and mass resulting from the deterioration of bone microstructure, which increases bone brittleness (Reid and Billington 2022). Osteoclasts, critical regulators of bone resorption in osteoporosis, mediate efficient bone degradation. Extensive research has sought to define the molecular pathways governing osteoclast differentiation and activation, ultimately aiming to advance therapeutic interventions for this condition (Takegahara et al. 2024). Emerging evidence indicates that ferroptosis may critically contribute to the pathogenesis of osteoporosis (Jiang et al. 2024; Ru et al. 2023). Heme oxygenase-1 (Hmox-1), a critical mediator of ferroptosis, regulates osteoclastogenesis to promote bone mass accrual. Notably, under high-iron microenvironments such as one triggered by Hmox-1 overexpression can trigger the elimination of osteoclast precursors (Kang et al. 2016; Zwerina et al. 2005). In our ongoing research on structurally novel, potent anti-osteoporosis compounds from deep-sea-derived microorganisms (He et al. 2023a, b; Xie et al. 2023, 2024, 2025; Zhang et al. 2023), the crude extract of *Penicillium limosum* ZEN48 obtained from the Western Pacific was subjected to a systematic chemical investigation. As a result, four new and 16 known indole-diketopiperazines were discovered (Fig. 1). Herein, we report the isolation, structural elucidation, and anti-osteoporosis bioactivities of these compounds.

## Materials and methods

### ECD calculation, GIAO NMR calculation, and DP4+ analysis

As previously reported, random searching in the Stochastic using the MMFF94 force field was applied for conformational analysis. Each conformer was successively optimized using the PM6 and HF/6-31G(d) levels. Conformers exhibiting dominance were additionally optimized at the B3LYP/6-31G(d) level in a gas phase. ECD calculations were conducted at B3LYP/6-311G(d,p) level in MeOH with IEFPCM model. Gaussian functions were overlapped to simulate the ECD spectrum for each transition. NMR calculations were carried out by the Gauge-Including Atomic Orbitals (GIAO) method at mMPW1PW91/6-311G(2d,p) level in DMSO simulated by the IEFPCM model. The TMS-corrected NMR chemical shift values were averaged according to Boltzmann distribution, finally the calculated ^13^C NMR data were obtained by the linear regression analysis method. The ^1^H and ^13^C chemical shifts were computed from NMR calculations, then the counted and experimental chemical shifts were applied in the excel to use DP4+ calculations according to the Boltzmann statistics using the computed sum of electronic and zero-point energies as the input. On the DP4+ analysis excel, functional mPW1PW91, basis set 6-31G(d) and solvent PCM were chosen to calculate.

### X-ray crystallographic analysis

The crystal data were recorded with an Xcalibur Eos Gemini single crystal diffractometer with Cu Kα radiation. The crystallographic data were deposited in the Cambridge Crystallographic Data Center (CCDC) with the deposition number of 2479054. Copies of the data can be obtained, free of charge, on application to CCDC, 12 Union Road, Cambridge CB21EZ, U.K. (fax +44(0)-1233-336033; email: depos it@ccdc.cam.ac.uk).

Compound **1**: Space group P2_1_2_1_2, *a* = 24.3342(4) Å, *b* = 10.8113(2) Å, *c* = 8.14360(10) (4) Å, *α* = β = *γ* = 90°, *V* = 2142.45(6) Å3, Z = 4, ρ_calcd_ = 1.276 g/cm^3^; *μ* = 0.690 mm^−1^, F(000) = 872.0; the final *R* indices [I > 2σ(I)] *R*_1_ = 0.0517, w*R*2 = 0.1293. The absolute structure parameter was − 0.02(12).

### BMMs cell extraction and culture

Bone marrow-derived monocytes (BMMs) were isolated from the femur of C57BL/6J mice aged 6-week-old with the methods as previously described (Xie et al. 2025). The BMMs were cultured with α-MEM complete medium containing 10% fetal bovine serum (gibco), 1% penicillin, streptomycin, and 25 ng/mL of macrophage colony-stimulating factor (M-CSF) (HY-P7085, Med-Chem Express, China).

### CCK-8 assay

The in vitro cytotoxic bioassay was conducted using the CCK-8 method according to the previously reported protocols (Xie et al. 2025). BMMs cells were inoculated in 96-well plates (5000 cells/well) and cultured with α-MEM complete medium containing M-CSF (25 ng/mL) for 24 h. The medium was replaced with medium containing compounds or without compounds for 48 h. Then 10 μL of CCK-8 (HY-K0301, Med-Chem Express, China) reagent to each well, and then placed in the incubator for 2 h. Finally, the cell viability was calculated by measuring the absorbance at 450 nm using an enzyme marker (TECAN, China).

### Osteoclast differentiation

BMMs were seeded in 96-well plates at a density of 5000 cells/well. Following overnight adherence, cells were maintained in α-MEM medium supplemented with 25 ng/mL M-CSF and 25 ng/mL receptor activator of nuclear factor-κB ligand (RANKL)(462-TEC-010/CF, R&D Systems, USA), containing test compounds. The culture medium was refreshed every 48 h for 5‒6 days.

### Trap staining

After osteoclast differentiation and compound treatment, the culture medium was aspirated from 96-well plates. Cells were then washed with PBS and fixed by adding 50 μL of 4% paraformaldehyde (PFA) per well for 30 min at room temperature. After fixation, the cells were washed three times with ultrapure water. Subsequently, the cells were stained with tartrate resistant acid phosphatase (TRAP) staining solution for 5 min. TRAP-positive multinucleated cells containing three or more nuclei were identified as osteoclasts and counted under a microscope (RIDET ZERO, China).

### F-actin staining

BMMs were seeded onto glass-bottom culture dishes (NEST, 801001-1; 20 mm diameter, TC-treated) at 33,000 cells/dish and allowed to adhere overnight. Cells were then cultured in α-MEM medium containing test compounds, supplemented with 25 ng/mL M-CSF and 25 ng/mL RANKL. The medium was replaced with fresh compound-containing induction medium every 48 h for 6 days. For cytoskeleton analysis, differentiated osteoclasts were fixed with 4% paraformaldehyde for 30 min at room temperature, followed by three washes with PBS. Cells were then permeabilized with 0.5% Triton X-100 in PBS for 10 min. F-actin was visualized by staining with phalloidin (Absin, abs47048271; 1:200 dilution) for 60 min, after which nuclei were counterstained with DAPI (P0131-25 mL, Beyotime, China). Samples were imaged using a confocal laser scanning microscope (ZEISS, LSM980 Airyscan2, Germany). The quantification of immunofluorescence was performed using ImageJ software.

### RT-qPCR

Total RNA was extracted from osteoblasts using the RNA isolater Total RNA Extraction Reagent reagent (R401-01, Vazyme, China) and reverse transcribed by Evo M-MLV Reverse Transcription Kit (AG11615, Accurate Biology, China) into cDNA. mRNA levels were subsequently determined by RT-qPCR using 2× Universal Blue SYBR Green qPCR Master Mix (G3326-15, Servicebio, China). The relative expression level of each gene was calculated by the 2^−ΔΔCt^ method.

### RNA-seq and data processing

BMMs were seeded in 6-well plates at 150,000 cells/well and cultured for 7 days in α-MEM medium containing 25 ng/mL M-CSF and 25 ng/mL RANKL, with or without 10 μmol/L compound **3**. Medium containing fresh compound and cytokines was replaced every 48 h. 0.1% dimethyl sulfoxide (DMSO) was used as a control. Total RNA was extracted using TRIzol reagent according to the manufacturer’s protocol as previously described (Xie et al. 2025). Differential expression analysis was performed using DESeq2 using the Ouyi Cloud platform, with a *P*-value < 0.05 and a fold change of > 1.5 or < − 1.5 being set as the screening threshold for differentially expressed genes (DEGs). Gene ontology (GO) and Kyoto Encyclopedia of Genes and Genomes (KEGG) pathway enrichment analyses of differentially expressed genes were performed using the Microbiology Letter platform, respectively, to screen for significantly enriched entries. GESA pathway enrichment was performed using the Ouyi Cloud platform. Protein–protein interaction (PPI) networks were constructed by the STRING database (https://cn.string-db.org/) and visualized using Cytoscape software (v3.10.1).

### Molecular docking

The crystal structure of Hmox-1 (ID: 5BTQ) was retrieved from the Protein Data Bank (PDB; https://www.rcsb.org). Molecular docking was performed through the Yinfu Cloud Computing Platform. The 3D structure of compound **3** and compound **4** were generated and energy-minimized using the MMFF94 force field. The binding pocket was defined based on the position of the co-crystallized ligand. Semi-flexible docking was performed using DOCK 6.9, and the resulting poses were evaluated using the Grid scoring function. Structural visualization was performed using PyMOL software. Hmox-1 was depicted as a gray cartoon model, while hydrogen bonds, π-cation interactions, and hydrophobic interactions were represented by cyan dashed lines, yellow dashed lines, and purple dashed lines, respectively.

### Detection of intracellular lipid peroxide

BMMs were seeded in glass-bottom culture dishes (801001-1 35 mm diameter, NEST, China) at a density of 3.0 × 10^4^ cells/dish and allowed to adhere overnight. Cells were cultured in α-MEM medium supplemented with 25 ng/mL M-CSF and 50 ng/mL RANKL. Starting on day 4, they were treated with either 0.1% DMSO (DMSO control) or 20 μmol/L compound **3**. Once mature osteoclasts were visibly formed, the medium was replaced with induced medium containing 5 μmol/L ferrostatin-1(Fer-1) (F408509, Aladdin, China) for 12 h. After treatment, the supernatant was removed and cells were washed twice with MEM medium, followed by incubation with BODIPY™ 581/591 C11 working solution for 30 min at 37 °C under 5% CO_2_. Finally, the staining solution was aspirated, cells were washed twice with HBSS, and then imaged using a confocal laser scanning microscope (ZEISS, LSM980 Airyscan2, Germany). The mean fluorescence intensity was quantified using ImageJ software.

## Results

### Structure elucidation

**Fig. 2:**
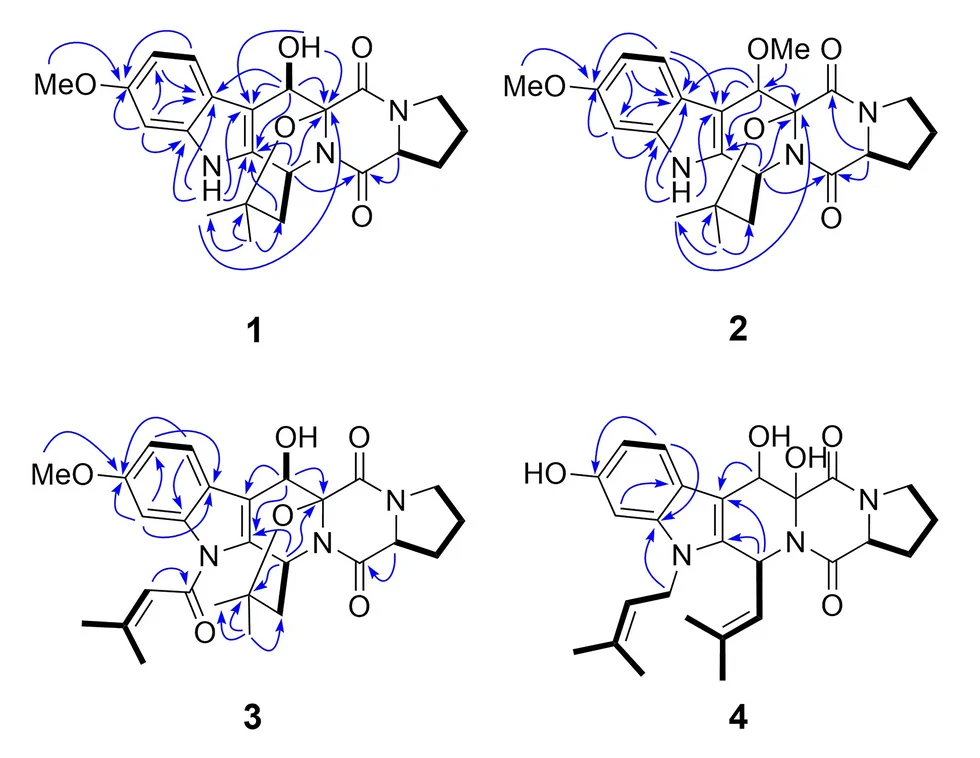
Key ^1^H-^1^H COSY (bold) and HMBC (arrow) correlations of **1–4**

Compound **1** was obtained as a white power. Its molecular formula was established as C_22_H_25_N_3_O_5_ on the basis of its positive HRESIMS peak at *m/z* 434.1692 [M + Na]^+^, suggesting twelve degrees of unsaturation. The ^1^H and ^13^C NMR spectra (Figures S1 and S2) in association with the HSQC spectrum revealed two methyl singlets, one methoxy group, four *sp*^*3*^ methylenes, three *sp*^*3*^ methines, three *sp*^*2*^ methines and nine non-hydrogenated carbons including two carbonyl, five olefinic as well as two *sp*^*3*^ carbons (Table 1). These resonances accounted for six degrees of unsaturation in total, suggesting that the molecule incorporated six rings. The HMBC correlations observed from NH-1 to C-2/C-14/C-15/C-20, H-16/C-18/20, H-17/C-15/19, OMe to C-18, together with the COSY correlations of H-16/H-17 indicated a 2,3-disubstited-6-methoyl indole substructure (Fig. 2). In addition, diagnostic HMBC cross-peaks from H-13 to C-2/12/14, H-3 to C-2/12 and COSY correlation of H-13/OH-13 constructed a third piperidine ring fused to the indole. A comparison of NMR data of **1** with that of the co-metabolite verruculogen TR-2 (**5**) (Fill et al. 2013), in association with the spin system of H-6/H_2_-7/H_2_-8/H_2_-9 in the COSY spectrum and HMBC correlations of H-3/H-6 to C-5, implied a similar diketopiperizine-containing hexacyclic scaffold in **1**. The final ring was assigned a 1,3-oxazinane formed through C-12–O–C-22 bridge, evident by the COSY cross-peak of H-3/H_2_-21, HMBC correlations from H_2_-21 to C-2, H_3_-23/H_3_-24 to C-21/22 as well as a diagnostic long-range signal from H_3_-23 to C-12. Therefore, the planar structure of **1** was established.

**Table 1:** ^1^H (600 MHz) and ^13^C (150 MHz) NMR spectroscopic data of compounds 1–2 (*δ* in ◊10^–6^, *J* in Hz within Parentheses)

No.	**1**^*a*^		**2**^*a*^	
	*δ*_C_	*δ*_H_	*δ*_C_	*δ*_H_
2	134.9 C		135.5 C	
3	41.6 CH	5.80 d (9.0)	41.7 CH	5.80 d (9.0)
5	164.5 C		164.6 C	
6	58.8 CH	4.23 dd (11.1, 6.1)	58.8 CH	4.20 dd (11.2, 6.1)
7	29.2 CH_2_	2.30 m; 1.79 m	29.2 CH_2_	2.31 m; 1.79 m
8	21.4 CH_2_	1.96 m; 1.89 m	21.3 CH_2_	1.98 m; 1.90 m
9	45.0 CH_2_	3.55 m; 3.45 m	45.0 CH_2_	3.55 m; 3.48 m
11	164.9 C		164.5 C	
12	86.4 C		84.9 C	
13	67.6 CH	4.68 d (7.3)	76.7 CH	4.55 s
14	108.0 C		105.4 C	
15	120.7 C		121.4 C	
16	118.6 CH	7.33 d (8.6)	118.8 CH	7.42 d (8.7)
17	109.0 CH	6.67 dd (8.6, 2.3)	109.4 CH	6.69 dd (8.7, 2.3)
18	155.5 C		155.5 C	
19	95.0 CH	6.88 d (2.3)	95.1 CH	6.90 d (2.3)
20	136.5 C		136.5 C	
21	39.2 CH_2_	2.13 dd (13.3, 9.0); 1.81 d (13.3)	39.5 CH_2_	2.14 dd (13.5, 9.0); 1.81 d (13.5)
22	73.0 C		73.3 C	
23	32.0 CH_3_	0.99 s	32.0 CH_3_	0.96 s
24	29.1 CH_3_	1.13 s	29.2 CH_3_	1.13 s
25	55.3 CH_3_	3.75 s	55.3 CH_3_	3.76 s
26			57.3 CH_3_	3.37 s
NH-1		10.91 s		11.05 s
13-OH		5.75 d (7.3)		

^*a*^Recorded in DMSO-*d*_6_

**Fig. 3:**
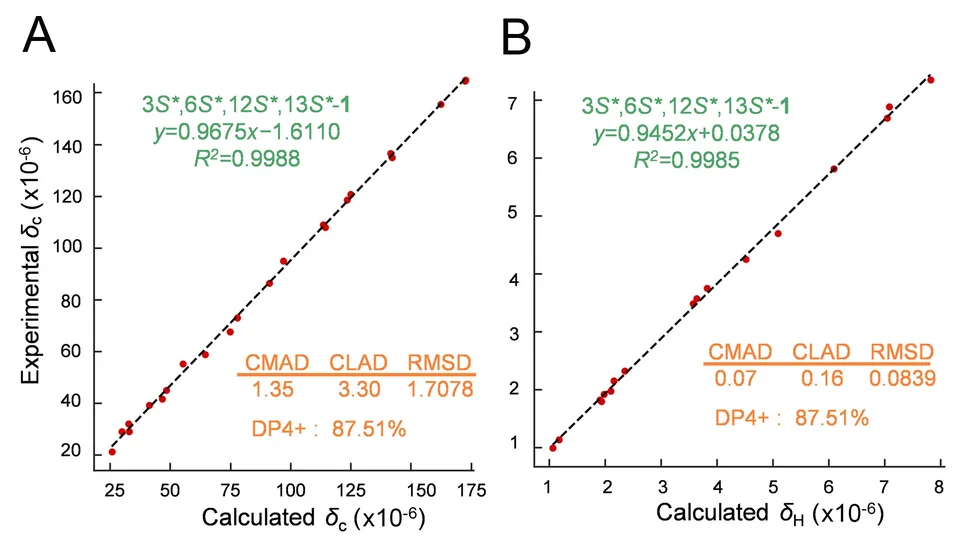
NMR calculation for **1**: Regression analysis of experimental and calculated ^13^C NMR and ^1^H chemical shifts for 3S*,6S*,12S*,13S*-**1**, with probability of each isomer analyzed by DP4+ included

**Fig. 4:**
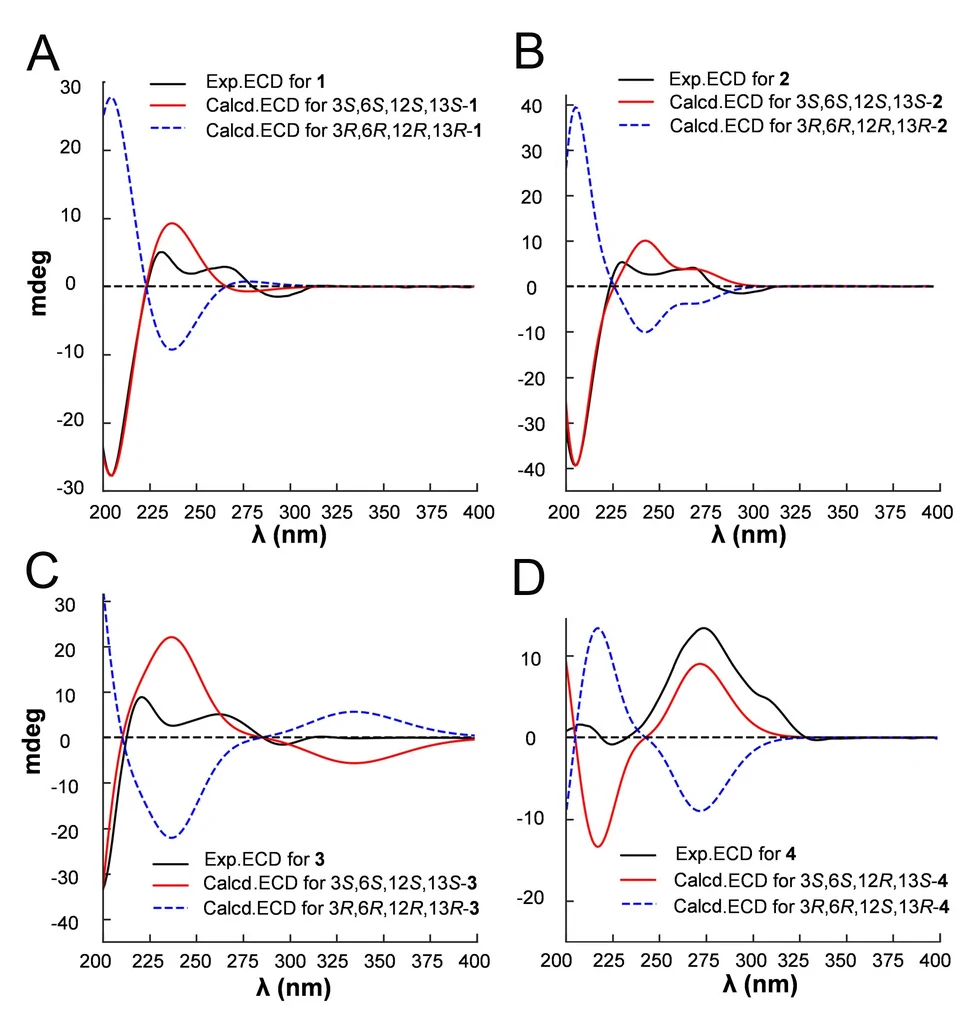
Calculated and experimental ECD spectra of **1–4**

**Fig. 5:**
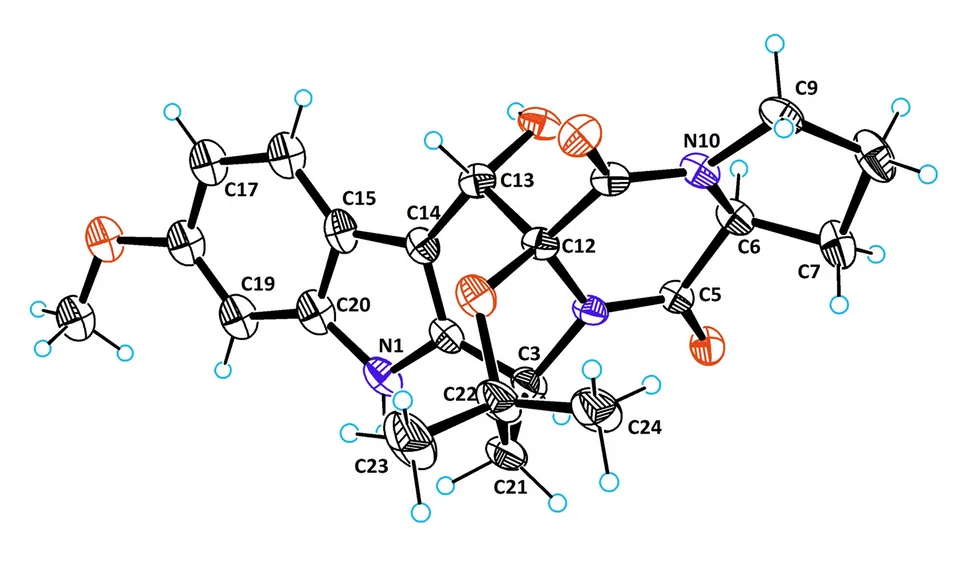
The single crystal X-ray crystallographic structure of **1**

Due to the relatively long distance among protons around chiral centers, the NOESY signals were insufficient to suggest the relative configuration of **1**. Therefore, quantum calculations of the NMR data for the four possible diastereomers, 3S*,6S*,12S*,13S*-1, 3S*,6S*,12S*,13R*-1, 3S*,6R*,12S*, 13S*-1, and 3S*,6R*,12S*, 13R*-1 (3R*,6S*,12R*, 13S*-1 in SI), were performed using the GIAO method at the mPW1PW91/6311+G (2d, p) level of theory (C-3 and C-12 were supposed to have fixed relative configuration due to the formation of the six-membered bridge ring) (Grimblat et al. 2015). This along with a subsequent DP4+ analysis strongly suggested the 3S*,6S*,12S*,13S*-isomer as the best option, which exhibited a 87.51% probability (Fig. 3). A further comparison of the calculated ECD spectrum of the isomer (3*S*,6*S*,12*S*,13*S*)-**1** with experimental data of **1** revealed a considerable agreement (Fig. 4), thereby establishing the absolute configuration of **1**. Fortunately, the crystal of **1** was obtained in the mixture of MeOH and DCM with a ratio of 10:1 by slowly evaporating. Finally, the complete structure of **1** was unambiguously confirmed by the single crystal diffraction analysis as shown (Fig. 5). On the basis of the above evidence, compound **1** was then assigned, and named limopiperazine A.

Compound **2** was isolated as a white powder. The molecular formula of C_23_H_27_N_3_O_5_ was established based on its HRESIMS spectrum at *m/z* 448.1820 [M + Na]^+^, suggestive of an analogue of **1**. The ^1^H and ^13^C NMR spectra (Table 1) were almost identical to those of **1** except that the hydroxy positioned at C-13 in **1** was replaced by a methoxy group (*δ*_H_ 3.37 s, *δ*_C_ 57.3 q), evident from the diagnostic HMBC correlation of the methoxy to C-13 (Fig. 2). Due to a biogenetic relationship with **1**, the origin of C-6 in **2** was assumed to be from l-Pro as **1**, thus retaining a *S* configuration. The relative and absolute configurations of remaining chiral centers of **2** were further confirmed to be identical as **1** by quantum calculation of the NMR chemical shifts (Figure S19) as well as ECD data (Fig. 4). On the basis of the above evidence, compound **2** was determined as a methylated analogue of **1** and named limopiperazine B.

The molecular formula of compound **3** was determined to be C_27_H_31_N_3_O_6_ according to the sodium adduct peak at *m/z* 516.2094 [M + Na]^+^ in its HRESIMS spectrum, indicating fourteen degrees of unsaturation. The ^1^H and ^13^C NMR spectra of **3** (Table 2) much resembled those of **1** with the primary difference the replacement of the NH-1 in **1** with a senecioyl unit in **3**, which was implied by the COSY correlations of H-28/H_3_-30/H_3_-31, together with the HMBC correlation observed from H-28 to C-27 (Fig. 2). The stereochemistry of **3** was assumed to be the same as those of **1** and **2** based on a biogenetic ground and further confirmed by quantum calculation of the NMR and ECD data (Fig. 4 and S29). On the basis of the above evidence, compound **3** was determined as a senecioylated analogue of **1** and named limopiperazine C.

**Table 2:** ^1^H (600 MHz) and ^13^C (150 MHz) NMR spectroscopic data of compounds 3–4 (*δ* in ◊10^–6^, *J* in Hz within Parentheses)

No.	**3**^*a*^		**4**^*b*^	
	*δ*_C_	*δ*_H_	*δ*_C_	*δ*_H_
2	135.7 C		131.4 C	
3	42.2 CH	6.24 d (9.3)	49.9 CH	6.01 d (10.1)
5	164.5 C		173.0 C	
6	58.9 CH	4.23 dd (11.1, 6.1)	60.2 CH	4.50 m
7	29.1 CH_2_	2.30 m; 1.80 m	30.1 CH_2_	2.42 m; 1.99 m
8	21.3 CH_2_	1.96 m; 1.90 m	23.5 CH_2_	2.07 m; 1.98 m
9	44.9 CH_2_	3.56 m; 3.46 m	46.5 CH_2_	3.60 m
11	164.4 C		168.1 C	
12	85.8 C		84.9 C	
13	66.9 CH	4.70 d (7.4)	69.9 CH	5.66 s
14	115.4 C		106.8 C	
15	122.1 C		121.5 C	
16	119.5 CH	7.47 d (8.6)	122.4 CH	7.72 d (8.5)
17	111.3 CH	6.94 dd (8.6, 2.1)	110.5 CH	6.59 dd (8.5, 2.1)
18	156.9 C		154.2 C	
19	100.0 CH	7.28 d (2.1)	96.3 CH	6.62 d (2.1)
20	135.4 C		139.8 C	
21	39.0 CH_2_	2.14 dd (13.8, 9.3); 1.91 d (13.8)	124.3 CH	4.75 m
22	73.3 C		136.2 C	
23	32.1 CH_3_	1.03 s	25.9 CH_3_	1.63 d (1.4)
24	28.6 CH_3_	1.14 s	18.7 CH_3_	1.96 d (1.4)
25	55.4 CH_3_	3.80 s		
27	165.3 C		42.6 CH_2_	4.55 m
28	119.5 CH	6.51 s	122.0 CH	5.01 m
29	157.1 C		135.5 C	
30	26.5 CH_3_	2.09 s	18.3 CH_3_	1.86 d (1.4)
31	20.7 CH_3_	2.10 s	25.7 CH_3_	1.70 d (1.4)
13-OH		6.13 d (7.4)		

^*a*^Recorded in DMSO-*d*_6_

^*b*^Recorded in CD_3_OD

The molecular formula of compound **4** was established as C_26_H_31_N_3_O_5_ by its positive HRESIMS signal at *m*/*z* 488.2158 [M + Na]^+^, requiring thirteen degrees of unsaturation. Analysis of the NMR data of **4** together with a comparison of those of **1**–**3** revealed resonances for a similar 5/5/6/6/5 pentacyclic framework as **1**–**3**, an isoprenyl group as well as an isobutenyl group, which accounted for thirteen degrees of unsaturation and suggested a lack of C-12–O–C-22 linkage in **4**. The diagnostic HMBC correlation from H_2_-27 to C-20 and COSY correlation of H-21/H-3 permitted the isoprenyl group at position N-1 and the isobutenyl group at C-3, respectively (Fig. 2). Regarding the stereochemistry, due to the common substitution at position C-12 of DKPs, the configuration of C-12 cannot be simply determined based on its the biosynthetic origin from L-tryptophan. A classic criterion for orientational determination of OH-12 is the marked down-filed proton chemical shift of H-21 (or H_2_-21), due to a deshielding effect caused by the hydroxy group towards a same side (Yu et al. 2017). Then the observation of a shielded chemical shift of H_2_-21 compared to known 3-prenylated DKPs (Cui et al. 1997) permitted an *anti-*relationship between OH-12 and H_2_-21. Furthermore, quantum calculations of the NMR data for the four possible diastereomers (3S*,6S*,12R*,13S*-4, 3S*,6S*,12R*,13R*-4, 3S*,6R*,12R*,13S*-4, and 3S*,6R*,12R*,13R*-4) were performed followed by DP4+ probability analysis which indicated the 3S*,6S*,12R*,13S* was the most likely (Figures S39 and S40). The absolute configuration was then confirmed by ECD calculation and comparison of the experimental ECD spectrum with theoretical data (Fig. 4). Therefore, the complete structure of **4** was assigned as shown, and named limopiperazine D.

By comparison of the NMR, HRMS, specific rotation data with those reported in references, the known compounds were identified as verruculogen TR-2 (**5**) (Fill et al. 2013), cyclotryprostatin E (**6**) (He et al. 2012), 2,3,11,12-tetrahydro-5a-hydroxy-12-(2-hydroxy-2-methylpropyl)-9-methoxy-trione (**7**) (Ding et al. 2013), asperfumigatin (**8**) (Xie et al. 2015), cyclotryprostatins A (**9**) (Cui et al. 1997), hydrocycloprostatin B (**10**) (Yu et al. 2017), verruculogen (**11**) (Fayos et al. 1974), 13-oxoverruculogen (**12**) (Wang et al. 2008), spirobrefeldin A (**13**) (Shi et al. 2022), spiro[5*H*,10*H*-dipyrrolo[1,2-*a*:1′,2′-*d*]pyrazine-2(3*H*),2′-[2*H*]indole]-3′,5,10(1′*H*)-trione (**14**) (Wang et al. 2008), spirotryprostatin G (**15**) (Zhang et al. 2019), isorugulosuvine (**16**) (Yin et al. 2010), *O*-demethylcyclopiamine B (**17**) (Ali et al. 2019), cyclopiamine B (**18**) (Ali et al. 2019), spiro[5*H*,6*H*-5a,9a-(iminomethano)-1*H*-cyclopent-[*f*]indolizine-7(8*H*) (**19**) (Onodera et al. 2013.1.9), 8*H*,10*H*-7a,12a-(iminomethano)indolizino[6,7-*h*]pyrano-[3,2-a]carbazole-1,16-(13*H*)-dione (**20**) (Onodera et al. 2013.1.9).

### Compounds activity on osteoclast differentiation

**Fig. 6:**
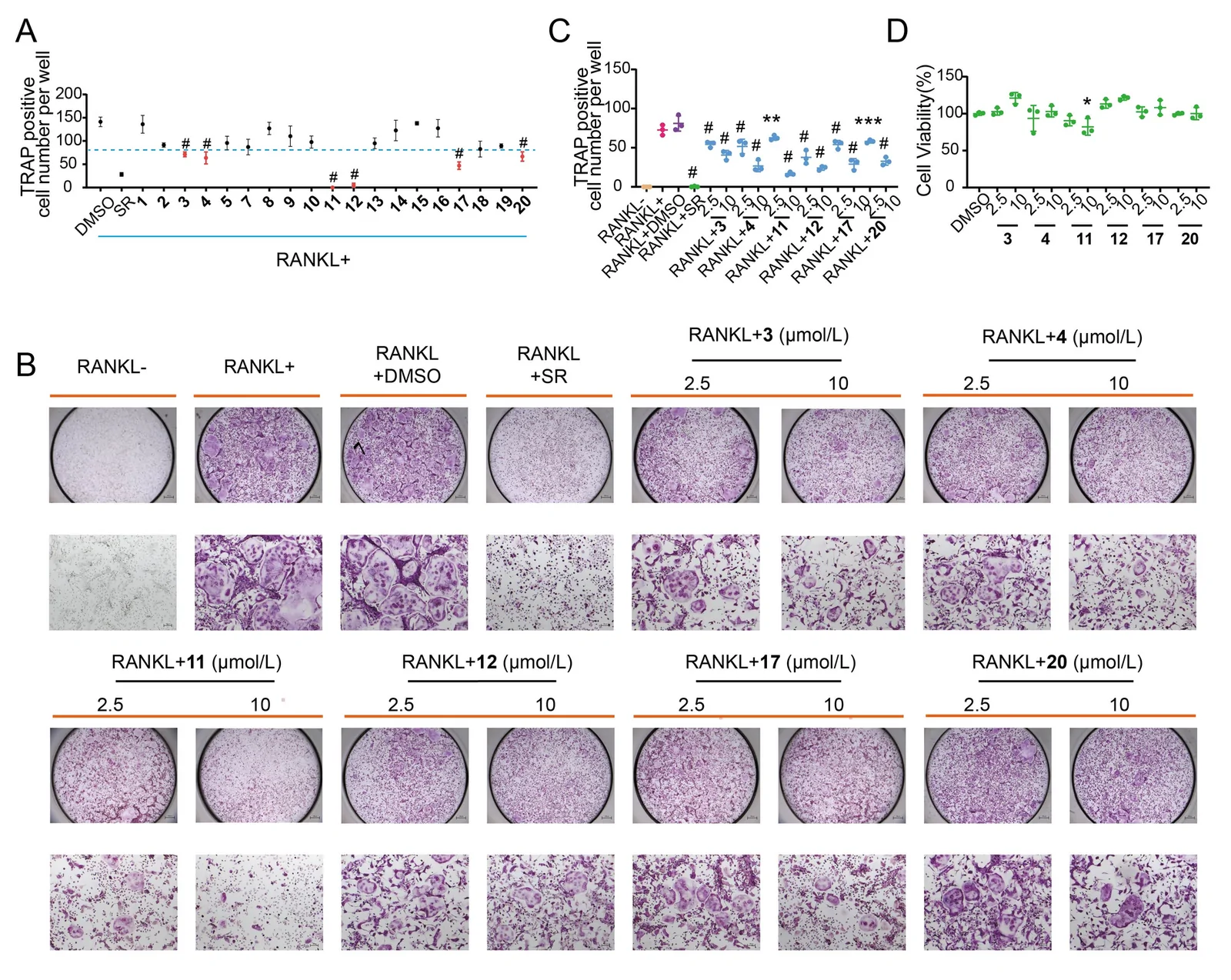
Six compounds suppress RANKL-induced osteoclastogenesis in BMMs. **A** Quantitative assessment of TRAP-positive multinucleated cells (> 3 nuclei) per well (*n* = 3). BMMs were treated with compounds (20 μmol/L) under the stimulation of RANKL (25 ng/mL) and MCSF (25 ng/mL) for 5 days. Then the cells were stained with TRAP staining solution and the number of osteoclasts was counted under a microscope. 0.1% Dimethyl sulfoxide (DMSO) and 1.5 mmol/L strontium ranelate (SR) were used as the solvent group and the positive control group, respectively. **B** Six compounds inhibited osteoclastogenesis in a dose-dependent manner. Representative images of osteoclasts following treatment with osteoclast inducers and compounds at concentrations of 2.5 μmol/L and 10 μmol/L. **C** Quantitative assessment of TRAP-positive multinucleated cells (> 3 nuclei) per well (*n* = 3). **D** The cytotoxicity of compounds on BMMs was measured by an CCK-8 assay (*n* = 3). All bar graphs are presented as the mean ± SD. ^*^P < 0.05, ^**^P < 0.01, ^***^P < 0.001, ^#^*P* < 0.0001 vs the DMSO (0.1%) group

The BMMs differentiate into osteoclasts upon stimulation with RANKL and macrophage colony-stimulating factor (M-CSF). Targeting this differentiation process represents a key therapeutic strategy for osteoporosis. In our ongoing effort to discover anti-osteoporotic natural products, all 20 compounds were evaluated for anti-osteoclastogenic activity using TRAP staining. Six compounds (**3**, **4**, **11**, **12**, **17**, **20**) significantly inhibited osteoclast formation at 20 µmol/L in BMMs (Fig. 6A). Meanwhile, we also explored the structure–activity relationship of osteoclast differentiation activity. Compounds **3** and** 1** share an identical diketopiperazine core; the sole structural difference is the presence of an isopentenyl group attached to the N-1 position in compound **3**. Osteoclast differentiation assays revealed that only compound **3** exhibited potent anti-osteoclastogenic activity, whereas compound **1** was inactive. This clearly indicates that the isopentenyl substitution at N-1 is a critical pharmacophore for activity in this diketopiperazine series. Furthermore, comparing compounds **1** and** 2**—which differ only by methylation of the 13-OH group—showed that neither compound displayed anti-osteoclastogenic activity. Thus, methylation at the 13-position is not essential for activity in this structural family. Similarly, modifications such as ketonation at C-27 or opening of the a 1,3-oxazinane ring formed via the C-12–O–C-22 bridge also proved to be non-essential for the observed bioactivity. Moreover, analysis of compounds** 5**–**12** suggests that the 8-membered ring linking C-22 and the isopentenyl moiety is a key structural feature required for activity. Finally, the marked activity of compounds **17** and **18**, compared to their non-hydroxylated analogues, indicates that hydroxyl groups on the phenyl ring substantially contribute to anti-osteoclastogenic potency. In summary, the anti-osteoclastogenic activity of this diketopiperazine series depends critically on the isopentenyl group at N-1 and the eight-membered ring bridging C-22, while modifications such as methylation of the 13-OH or opening of the 1,3-oxazinane ring are not essential for activity. Moreover, these active compounds suppressed osteoclast differentiation in a concentration-dependent manner (Fig. 6B and C). All compounds showed no cytotoxicity except for **11** at 10 μmol/L (Fig. 6D).

### Compounds disrupt actin ring formation in osteoclasts

**Fig. 7:**
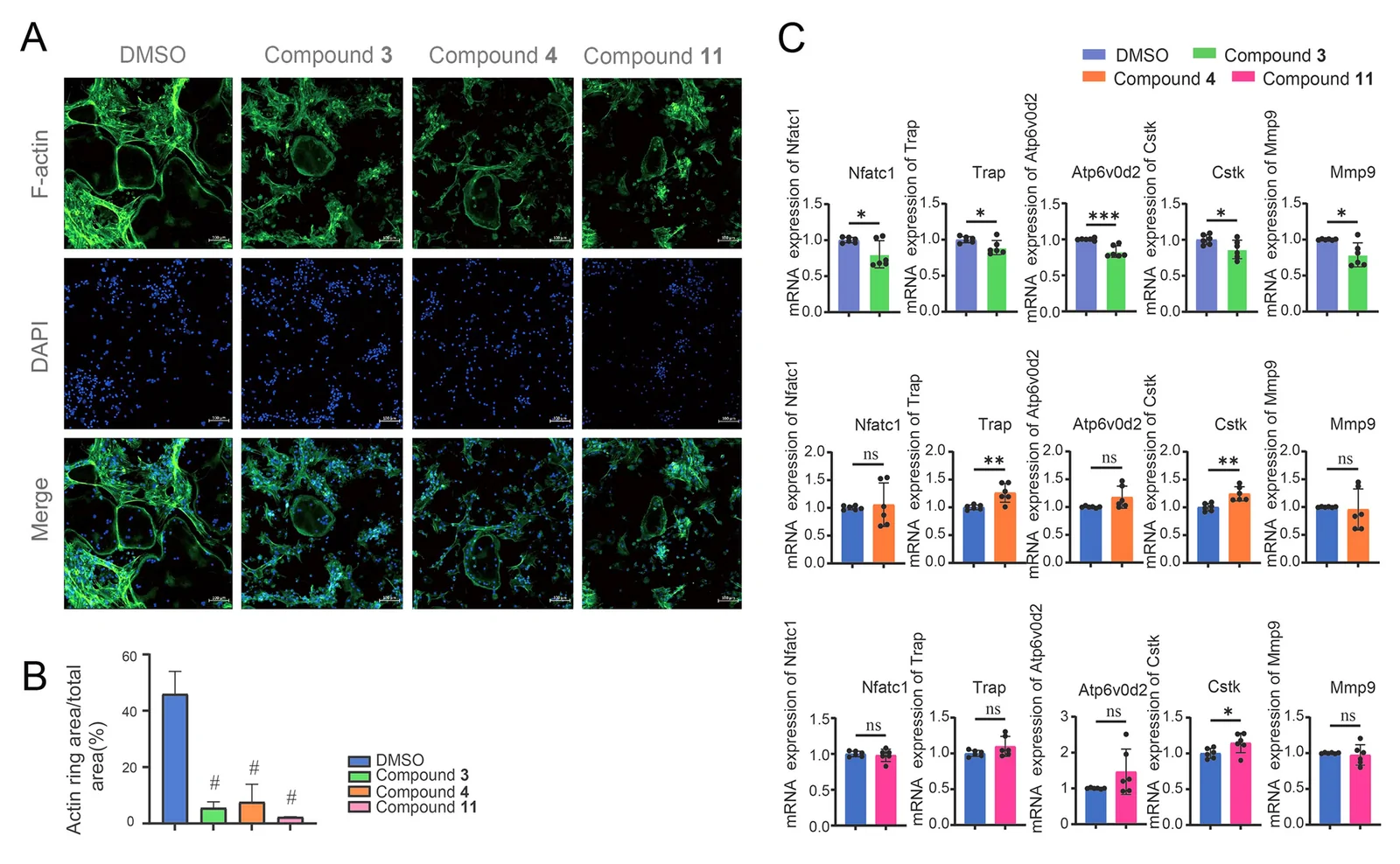
Compounds **3**, **4**, and **11** disrupt actin ring formation, while compound **3** suppresses osteoclast-related gene expression. **A** Actin cytoskeleton staining in RANKL/M-CSF-induced osteoclasts. BMMs were stimulated with RANKL (25 ng/mL) and M-CSF (25 ng/mL) for 6 days with **3** (10 μmol/L), **4** (10 μmol/L), or **11** (2.5 μmol/L). Actin rings were visualized by phalloidin immunofluorescence (scale bar: 50 μm). **B** Quantification of F-actin ring area per field (%) (*n* = 3). **C** RT-qPCR analysis of osteoclastogenesis markers. BMMs were treated with compound** 3** (10 μmol/L),** 4** (10 μmol/L), or **11** (2.5 μmol/L). Data represent mean ± SD (*n* = 6); ns, not significant, *P < 0.05, **P < 0.01, ***P < 0.001 and ^#^*P* < 0.0001 *vs* DMSO (0.1% DMSO)

Notably, osteoclast differentiation requires multinucleation via cell fusion, polarization to form resorptive functional zones, and actin cytoskeleton reorganization. Phalloidin staining demonstrated that compounds **3**,** 4**, and **11** disrupted actin ring formation in osteoclasts (Fig. 7A and B). Critically, only compound **3** significantly downregulated core osteoclastogenic markers (Fig. 7C), including nuclear factor of activated T-cells 1 (*Nfatc1*), *Trap*, ATPase H+ transporting V0 subunit D2 (*Atp6v0d2*), cathepsin K (*Ctsk*), and matrix metalloproteinase 9 (*Mmp9*).

### Compound 3 inhibits osteoclastogenesis via ferroptosis

**Fig. 8:**
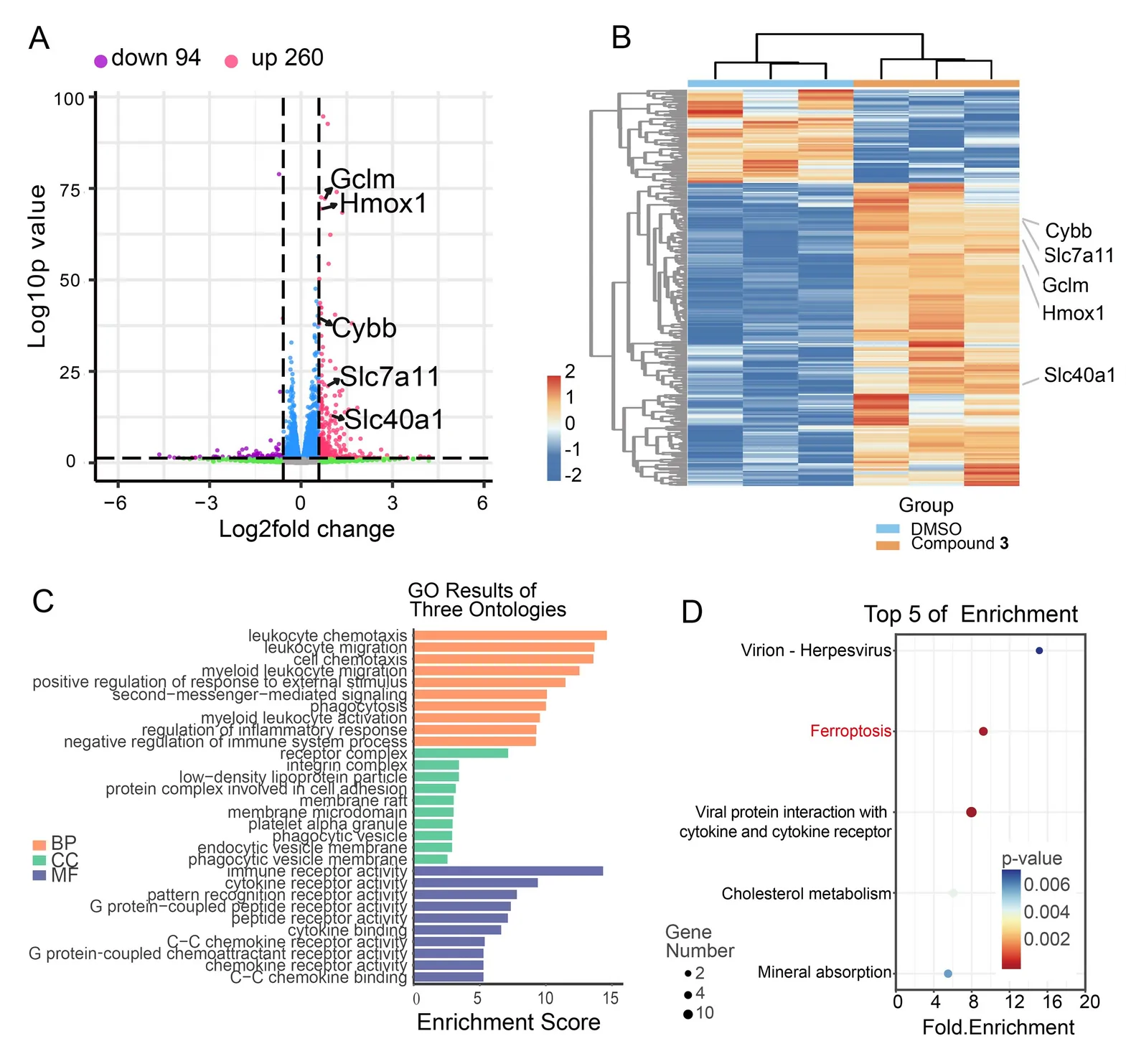
RNA-seq reveals that compound **3** inhibits osteoclast formation via the ferroptosis pathway. **A** Volcano plot of DEGs in BMMs treated with compound **3** versus the DMSO control. Downregulated genes (purple); upregulated genes (red). **B** Heatmap analysis of DEGs expression profiles. **C** GO enrichment analysis showing significantly upregulated biological processes (TOP10) in compound **3**-treated BMMs. **D** KEGG pathway enrichment (TOP 5)

RNA-seq was performed to elucidate the mechanism by which compound **3** inhibits osteoclastogenesis in BMMs cells. Volcano plots and heatmap analysis revealed significant differential expressed genes (DEGs) between compound **3**-treated and untreated cells, identifying 260 upregulated and 94 downregulated genes (Fig. 8A and B). GO enrichment analysis revealed that these DEGs are significantly involved immune receptor activity, receptor complex and leukocyte chemotaxis across molecular function (MF), cellular component (CC), and biological process (BP) categories (Fig. 8C). This aligns with established roles of immune regulation in bone homeostasis: regulatory T cells (Tregs) suppress osteoclast differentiation via anti-inflammatory cytokines (e.g. IL-10) (Luo et al. 2011); Pro-inflammatory factors (TNFα, IL-1β, IL-6) promote osteoclastogenesis (Redlich and Smolen 2012). Noteworthily, both KEGG and Gene Set Enrichment Analysis (GSEA) analyses consistently identified the ferroptosis pathway as one of the most significantly enriched mechanisms through which compound **3** modulates osteoclast activity. This pathway was prioritized for further investigation not only due to its high enrichment fold and statistical significance (ranking as the second most enriched KEGG pathway), but also because of its emerging biological relevance to osteoclast differentiation and function (Fig. 8D and Figure S73).

### Compound 3 activates ferroptosis by targeting Hmox-1

**Fig. 9:**
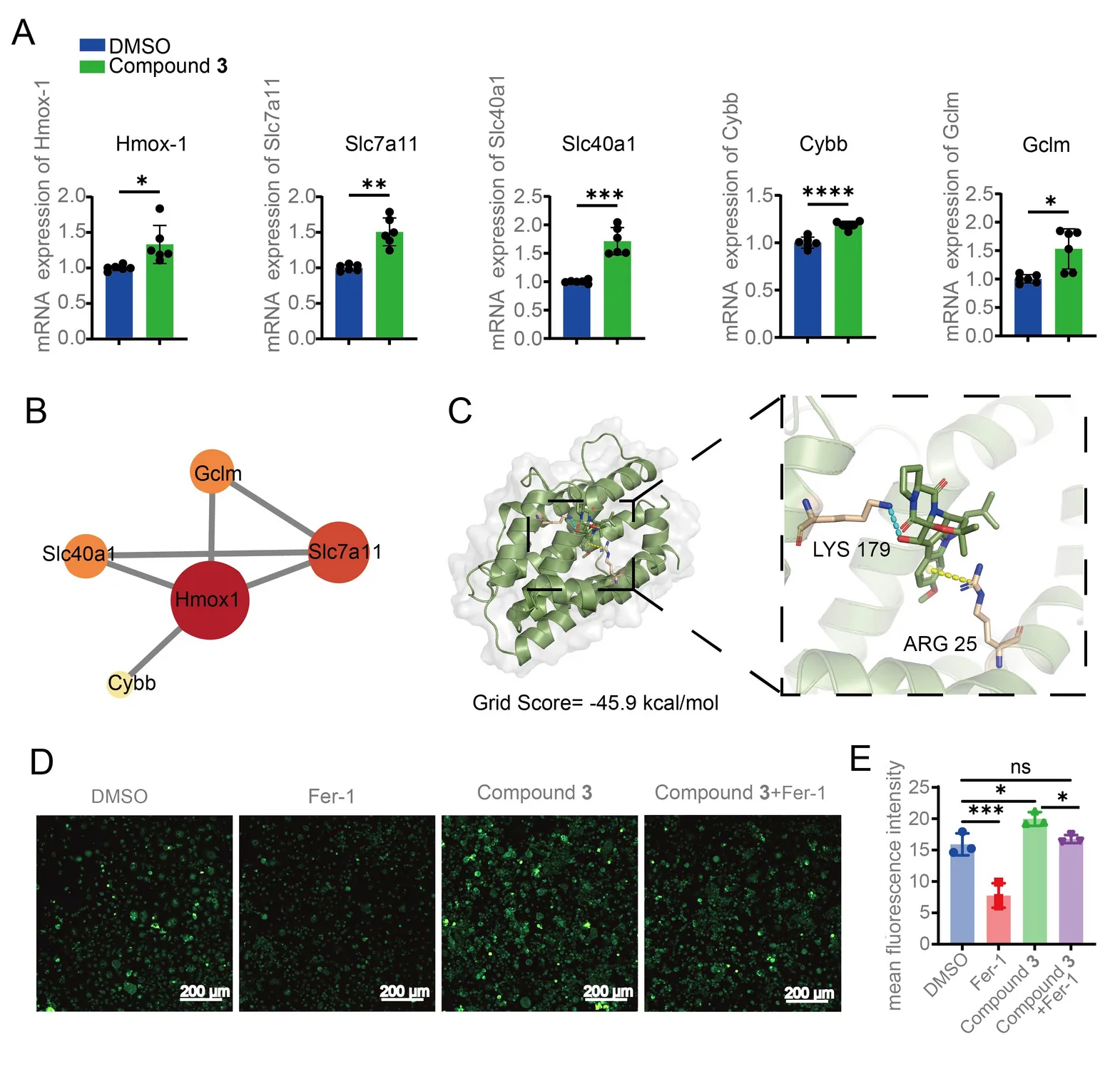
Compound **3** inhibits osteoclastogenesis by targeting Hmox‑1 via ferroptosis. **A** RT‑qPCR analysis of ferroptosis‑related DEGs markers (*n* = 6). **B** PPI network of ferroptosis‑associated DEGs. **C** Molecular docking pose of compound **3** bound to Hmox-1. **D** BODIPY™ C11 staining of lipid peroxidationScale bar = 200 μm. **E** Quantification of mean fluorescence intensity from **D**. Data are presented as mean ± SD. *P < 0.05, **P < 0.01, ***P < 0.001, ****P < 0.0001 vs. DMSO (0.1%) control

Furthermore, RT-qPCR analysis of ferroptosis-related DEGs confirmed that compound **3** activates ferroptosis in osteoclasts (Fig. 9A). PPI network mapping of ferroptosis-related DEGs identified Hmox-1 as the central hub (5 nodes, 6 edges) (Fig. 9B). As a key ferroptosis regulator, Hmox-1 upregulation activates antioxidant systems that inhibit BMM-derived osteoclast activation (Chen et al. 2024; Ma et al. 2024; Peng et al. 2025). This regulation is mediated through complex interactions with various cellular pathways and transcription factors, highlighting the potential of targeting Hmox-1 and its associated pathways in therapeutic strategies for diseases involving oxidative stress and bone metabolism dysregulation. Therefore, Hmox-1 was identified as a major target of compound **3** in the ferroptosis signaling pathway. To investigate whether compound **3** binds to Hmox-1, we focused our analysis on protein–ligand interactions, identifying and classifying all functional residues according to their specific interactions. It was found that multiple sets of residues are involved in the interaction between Hmox-1 and compound **3**, including the hydrogen bonding formed by LYS179 of Hmox-1 with the ligand, and the π-cation interaction formed by ARG25 of Hmox-1 with the ligand. These interactions are functionally relevant: LYS179 lies within a domain involved in heme binding and protein–partner interactions, suggesting that compound **3** may allosterically modulate Hmox-1 activity. ARG25, located near the active-site entrance, plays a role in substrate/product channeling; engagement by compound **3** could alter local conformational dynamics or electrostatic properties. These interacting forces resulted in a protein–ligand complex of − 45.9 kcal/mol (Grid Score) binding energy, indicating a strong binding force, and thus compound **3** inhibited osteoclast formation by targeting Hmox-1 (Fig. 9C).

To functionally confirm the role of ferroptosis, lipid peroxidation, a key marker of this process, was measured using BODIPY™ 581/591 C11 staining in cells treated with compound **3**. We further applied the ferroptosis inhibitor Fer-1 to examine whether the anti-osteoclastogenic effect of the compound could be reversed, thus testing whether ferroptosis is necessary for its activity. As shown in Fig. 9D, compound **3** significantly increased lipid peroxidation compared to the DMSO control, whereas Fer-1 alone reduced basal peroxidation. Co-treatment with compound **3** and Fer-1 partially but not fully reversed the peroxidation induced by compound **3**, supporting the involvement of ferroptosis in its mechanism.

## Discussion

The 2,5-diketopiperazines (2,5-DKPs) are widespread in fungal metabolites, of which the indole tryptophan-proline DKPs cyclo(l-Trp-l-Pro) core constitutes the most abundant examples (Borthwick 2012). Heterocyclization and isoprenyl addition on the core are the main modifications that lead to the structure diversity of 2,5-DKPs. For instance, the prenylation for the tryptophan-incorporating DKPs can occur at any position on the tryptophan residue including the N atom while the isoprenyl units have multiple forms when released from the precursor dimethylallkyl diphosphate (DMAPP) or isopentenyl diphosphate (IPP) (Raju et al. 2011). Limopiperazines A–C (**1**–**3**), with prenylation at C-2 position of the tryptophan residue, represent the sole exemplar of 2,5-DKPs bearing a bridged 1,3-oxazinane ring formed between the C2-isoprenyl and α carbon hydroxy of Trp moiety. Limopiperazine D (**4**) features isoprenyl groups at both positions C-2 and N atom of tryptophan residue. Meanwhile, apart from the bioactivities for 2,5-DKPs of marine origin in the past (Song et al. 2021), our study provides the first report of this natural product backbone with regulatory effect on osteoclast differentiation. Bone remodeling is a tightly regulated process that maintains skeletal homeostasis through the balanced activities of bone-resorbing osteoclasts and bone-forming osteoblasts. Disruption of this equilibrium can lead to metabolic bone diseases such as osteoporosis. The clinical management of osteoporosis continues to face significant challenges despite available antiresorptive therapies (Compston et al. 2019; Reid and Billington 2022). Mainstay treatments, particularly bisphosphonates and the anti-RANKL antibody denosumab, effectively reduce bone resorption but are associated with substantial limitations. Long-term bisphosphonate use has been linked to atypical femoral fractures and osteonecrosis of the jaw due to excessive suppression of bone turnover, while denosumab treatment carries the risk of rapid bone loss and multiple vertebral fractures upon discontinuation (Compston 2017). Although these drugs are effective, the long-term use of these therapeutics is often limited by their adverse effects. As a result, there is a clear and pressing need to develop novel chemical entities or derived compounds with effective antiresorptive properties. In this study, the new compound limopiperazine C (**3**) potently inhibited osteoclast differentiation and disrupted actin ring formation. This provides a new chemical entity for the development of anti-osteoporosis drugs in the future.

Marine natural products have recently gained attention as promising therapeutic candidates for osteoporosis due to their structural diversity and unique bioactivities (El-Desoky and Tsukamoto 2022). However, the active mechanisms of most marine natural products are not clear. RNA-seq technology has become instrumental in elucidating the mechanisms underlying these effects (Hansen et al. 2024; Liu et al. 2024; Xie et al. 2025). By profiling global transcriptomic changes, RNA-seq enables the identification of key pathways through which marine compounds exert their protective actions. This high-throughput approach facilitates the unbiased discovery of novel targets and compound–gene interactions, providing a systems-level understanding of how marine compounds influence bone cell viability and function. In our study, integrating RNA-seq with functional assays offers a powerful strategy to accelerate the development of marine-derived therapies that selectively target pathological processes such as ferroptosis.

In conclusion, our investigation into the deep-sea-derived *Penicillium limosum* ZEN48 led to the discovery of four new (**1**–**4**) and 16 known (**5**–**20**) compounds. Among them limopiperazines A–C (**1**–**3**) feature an unprecedented 6/5/6/6/6/5 hexacyclic diketopiperazine framework with one or two prenylation positions. Moreover, limopiperazine C exhibits potent inhibitory effect against osteoclast differentiation and disrupts actin ring formation. The integration of RNA-seq, RT-qPCR and molecular docking analysis revealed the possible molecular target of limopiperazine C on this anti-osteoclastogenic effect as Hmox-1, which functions as a modulator of the ferroptosis signaling pathway. Our study highlights that the diketopiperazines can serve as potential scaffold in the development of anti-osteoporotic agents.

## Supplementary Information

Below is the link to the electronic supplementary material.

Supplementary file1 (PDF 14945 KB)

## Data Availability

Any information can be requested from the first author.
