# Supplementary material for: Novel prenylated diketopiperazines with anti-osteoclastogenic activity from the deep-sea-derived Penicillium limosum ZEN48
Source: Mar Life Sci Technol. 2026 May 19;8(3):922–34. doi: 10.1007/s42995-026-00385-2 (PMC13525149; doi:10.1007/s42995-026-00385-2)
Supplement: Supplementary file 1 [file 42995_2026_385_MOESM1_ESM.pdf]

Supporting Information

Novel Prenylated Diketopiperazines with Anti-Osteoclastogenic Activity from the Deep-Sea-Derived *Penicillium limosum* ZEN48

Yong Zhang,[a,b,c,1] Yan-Qing Guo,[a,b,1] You Li,[a] Zheng-Biao Zou,[a] Zhe-Wen Chen,[c] Rong Zhao,[c] Robert J. Capon,[d] Tai-Zong Wu,[c,*] Chun-Lan Xie,[b,*] and Xian-Wen Yang[a,*]

[a] Hainan Academy of Medical Sciences, Hainan Medical University, 3 Xueyuan Road, Haikou 571199, China

[b] Key Laboratory of Tropical Translational Medicine of Ministry of Education, School of Basic Medicine and Life Sciences, Hainan Medical University, 3 Xueyuan Road, Haikou 571199, China

[c] Key Laboratory of Marine Genetic Resources, Third Institute of Oceanography, Ministry of Natural Sources, 184 Daxue Road, Xiamen 361005, China

[d] Institute for Molecular Bioscience, The University of Queensland, Brisbane, QLD 4072, Australia

[1] Y, Z, and Y-Q, G. contributed equally

Content

Figure S1. ^{1}H NMR spectrum of **1** in DMSO-d_6. 1
Figure S2. ^{13}C NMR spectrum of **1** in DMSO-d_6. 2
Figure S3. HSQC spectrum of **1** in DMSO-d_6. 3
Figure S4. ^{1}H-^{1}H COSY spectrum of **1** in DMSO-d_6. 4
Figure S5. HMBC spectrum of **1** in DMSO-d_6. 5
Figure S6. NOESY spectrum of **1** in DMSO-d_6. 6
Figure S7. HR-ESI-MS spectrum of **1**. 7
Figure S8. Specific rotation data of **1**. 8
Figure S9. Regression analysis of experimental versus calculated ^{1}H, ^{13}C NMR chemical shifts of **1**. 9
Figure S10. The DP4+ probability analysis of **1**. 10
Figure S11. ^{1}H NMR spectrum of **2** in DMSO-d_6. 11
Figure S12. ^{13}C NMR spectrum of **2** in DMSO-d_6. 12
Figure S13. HSQC spectrum of **2** in DMSO-d_6. 13
Figure S14. ^{1}H-^{1}H COSY spectrum of **2** in DMSO-d_6. 14
Figure S15. HMBC spectrum of **2** in DMSO-d_6. 15
Figure S16. NOESY spectrum of **2** in DMSO-d_6. 16
Figure S17. HR-ESI-MS spectrum of **2**. 17
Figure S18. Specific rotation data of **2**. 18
Figure S19. Regression analysis of experimental versus calculated ^{1}H, ^{13}C NMR chemical shifts of **2**. 19
Figure S20. The DP4+ probability analysis of **2**. 19
Figure S21. ^{1}H NMR spectrum of **3** in DMSO-d_6. 20
Figure S22. ^{13}C NMR spectrum of **3** in DMSO-d_6. 21
Figure S23. HSQC spectrum of **3** in DMSO-d_6. 22
Figure S24. ^{1}H-^{1}H COSY spectrum of **3** in DMSO-d_6. 23
Figure S25. HMBC spectrum of **3** in DMSO-d_6. 24
Figure S26. NOESY spectrum of **3** in DMSO-d_6. 25
Figure S27. HR-ESI-MS spectrum of **3**. 26
Figure S28. Specific rotation data of **3**. 27
Figure S29. Regression analysis of experimental versus calculated ^{1}H, ^{13}C NMR chemical shifts of **3**. 28
Figure S30. The DP4+ probability analysis of **3**. 28
Figure S31. ^{1}H NMR spectrum of **4** in CD_3OD. 29
Figure S32. ^{13}C NMR spectrum of **4** in CD_3OD. 30
Figure S33. HSQC spectrum of **4** in CD_3OD. 31
Figure S34. ^{1}H-^{1}H COSY spectrum of **4** in CD_3OD. 32
Figure S35. HMBC spectrum of **4** in CD_3OD. 33
Figure S36. NOESY spectrum of **4** in CD_3OD. 34
Figure S37. HR-ESI-MS spectrum of **4**. 35
Figure S38. Specific rotation data of **4**. 36
Figure S39. Regression analysis of experimental versus calculated ^{1}H, ^{13}C

NMR chemical shifts of **4.** 37
Figure S40. The DP4+ probability analysis of **4.** 38
Figure S41. ^{1}H NMR spectrum of **5** in $CDCl_3$. 39
Figure S42. HR-ESI-MS spectrum of **5**...40
Figure S43. ^{1}H NMR spectrum of **6** in CD_3OD. 41
Figure S44. HR-ESI-MS spectrum of **6**...42
Figure S45. ^{1}H NMR spectrum of **7** in CD_3OD. 43
Figure S46. HR-ESI-MS spectrum of **7**...44
Figure S47. ^{1}H NMR spectrum of **8** in DMSO-d_6. 45
Figure S48. HR-ESI-MS spectrum of **8**...46
Figure S49. ^{1}H NMR spectrum of **9** in DMSO-d_6. 47
Figure S50. HR-ESI-MS spectrum of **9**...48
Figure S51. ^{1}H NMR spectrum of **10** in DMSO-d_6. 49
Figure S52. HR-ESI-MS spectrum of **10**. 50
Figure S53. ^{1}H NMR spectrum of **11** in DMSO-d_6. 51
Figure S54. HR-ESI-MS spectrum of **11**...52
Figure S55. ^{1}H NMR spectrum of **12** in DMSO-d_6. 53
Figure S56. HR-ESI-MS spectrum of **12**. 54
Figure S57. ^{1}H NMR spectrum of **13** in CD_3OD. 55
Figure S58. HR-ESI-MS spectrum of **13**. 56
Figure S59. ^{1}H NMR spectrum of **14** in CD_3OD. 57
Figure S60. HR-ESI-MS spectrum of **14**. 58
Figure S61. ^{1}H NMR spectrum of **15** in DMSO-d_6. 59
Figure S62. HR-ESI-MS spectrum of **15**. 60
Figure S63. ^{1}H NMR spectrum of **16** in CD_3OD. 61
Figure S64. HR-ESI-MS spectrum of **16**. 62
Figure S65. ^{1}H NMR spectrum of **17** in $CDCl_3$. 63
Figure S66. HR-ESI-MS spectrum of **17**. 64
Figure S67. ^{1}H NMR spectrum of **18** in CD_3OD. 65
Figure S68. HR-ESI-MS spectrum of **18**. 66
Figure S69. ^{1}H NMR spectrum of **19** in CD_3OD. 67
Figure S70. HR-ESI-MS spectrum of **19**. 68
Figure S71. ^{1}H NMR spectrum of **20** in CD_3OD. 69
Figure S72. HR-ESI-MS spectrum of **20**. 70
Figure S73. Compund **3** inhibits osteoclastogenesis via ferroptosis GSEA of ferroptosis-related pathways..71
Figure S74. Molecular docking conformation of compounds **3** and **4** bound to HO-1. 72
Figure S75. Effect of Fer-1 on BMMs viability measured by the CCK-8 assay. 74
Table S1. Primers used in this work 75
Table S2. Gibbs fee energyand equilibrium populations of low-energy conformers of **1-4** in NMR calculations...76

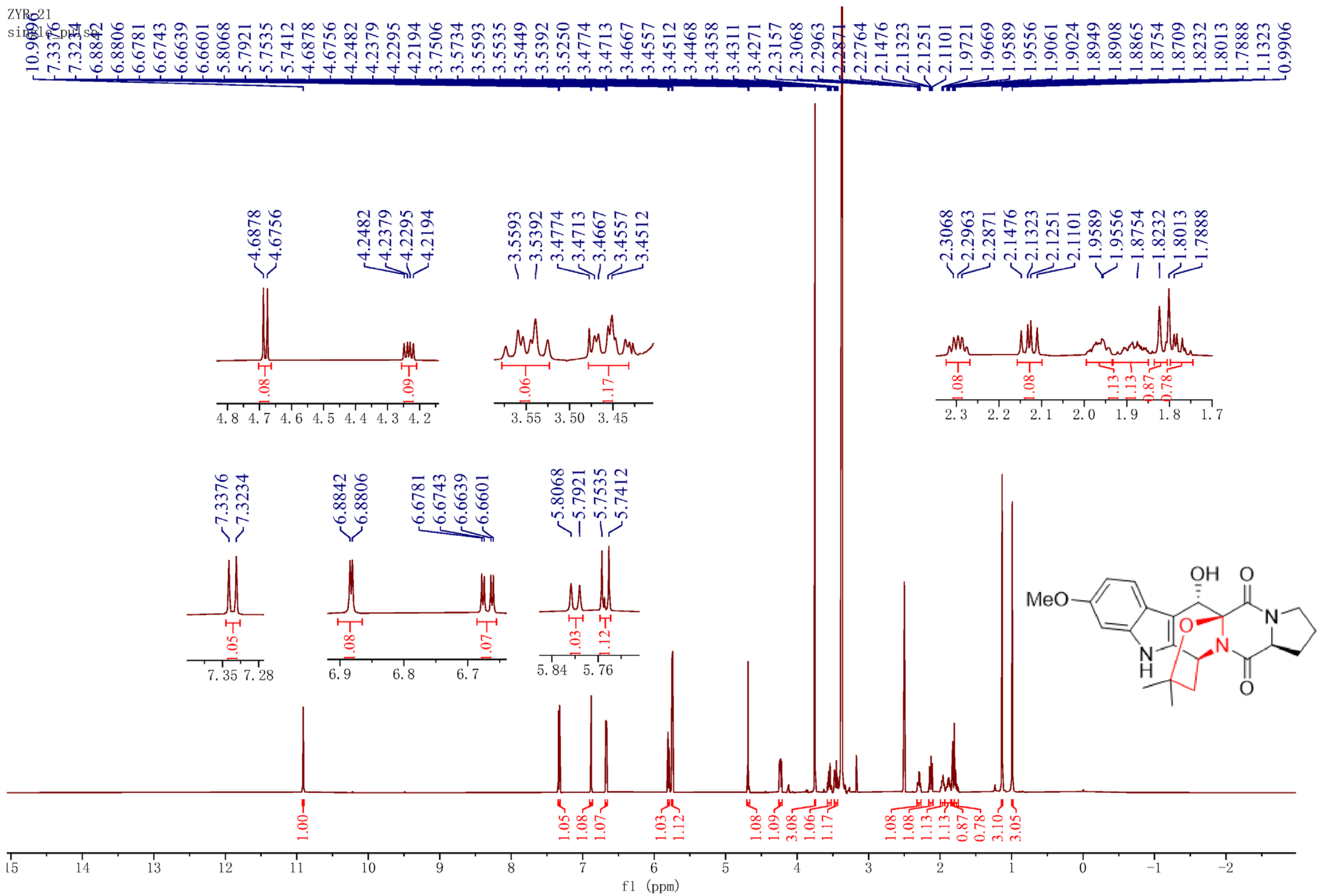

Figure S1. ^{1}H NMR spectrum of **1** in DMSO-d_6.

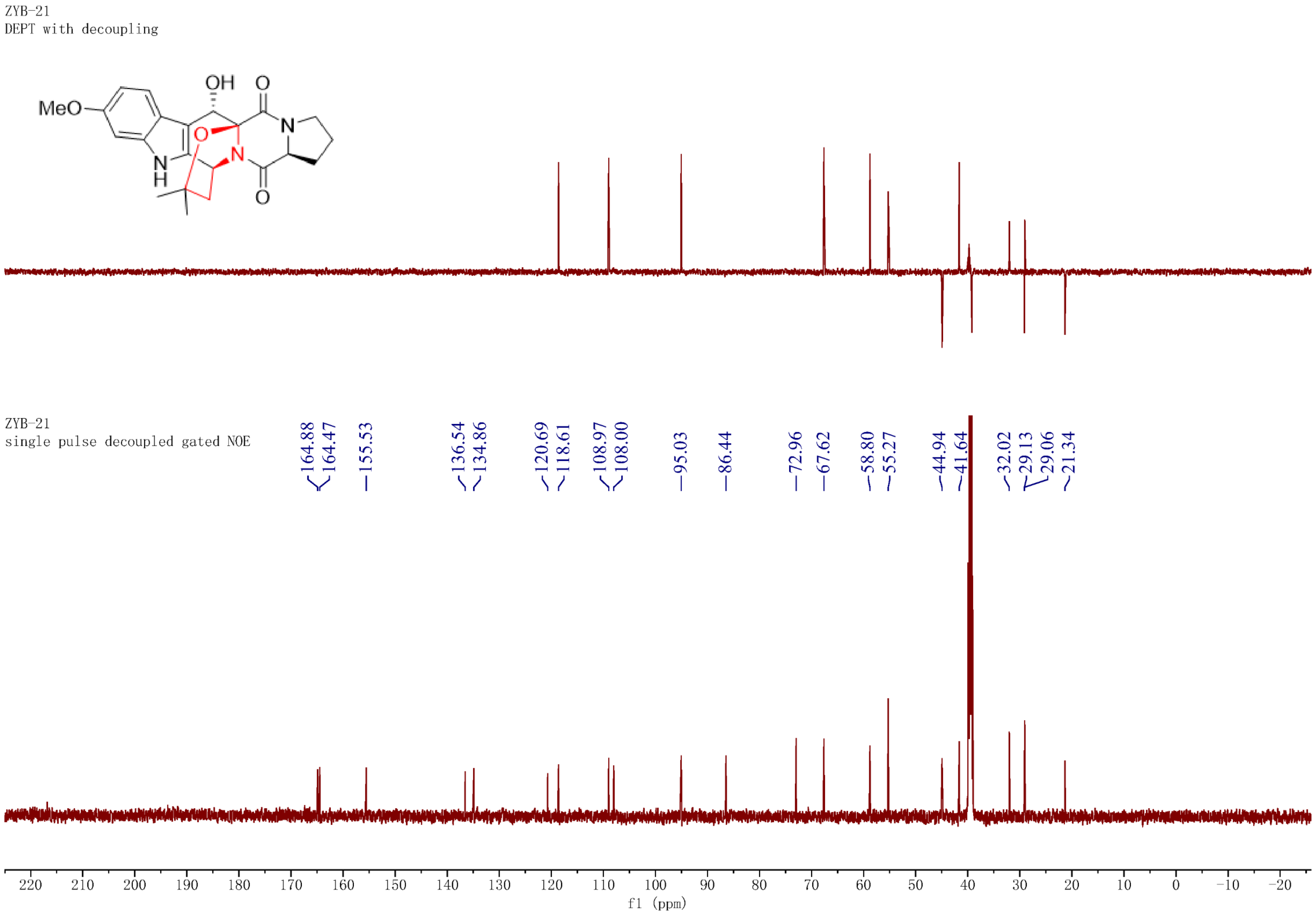

Figure S2. ^{13}C NMR spectrum of **1** in DMSO-d_6.

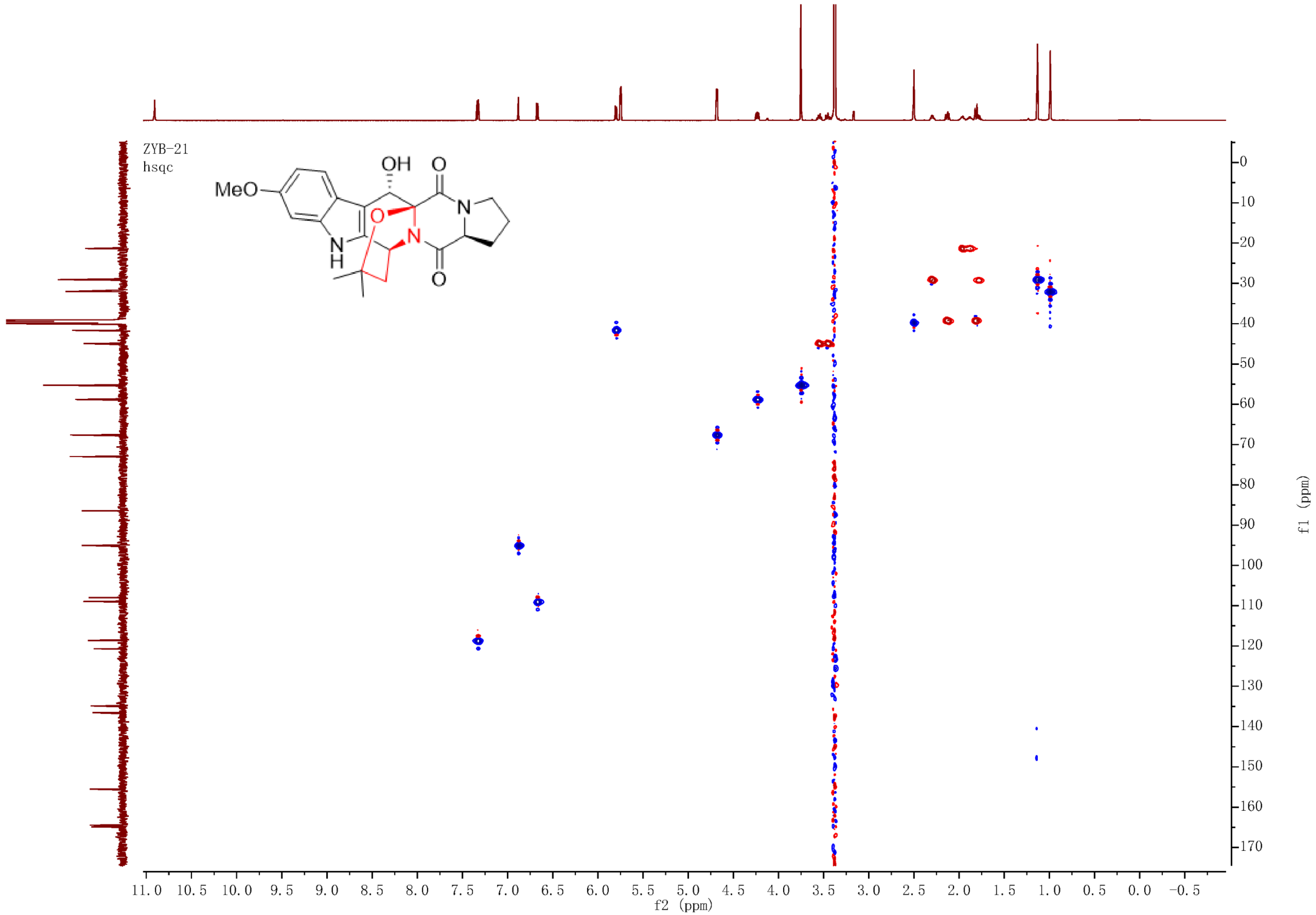

Figure S3. HSQC spectrum of **1** in DMSO-d_6.

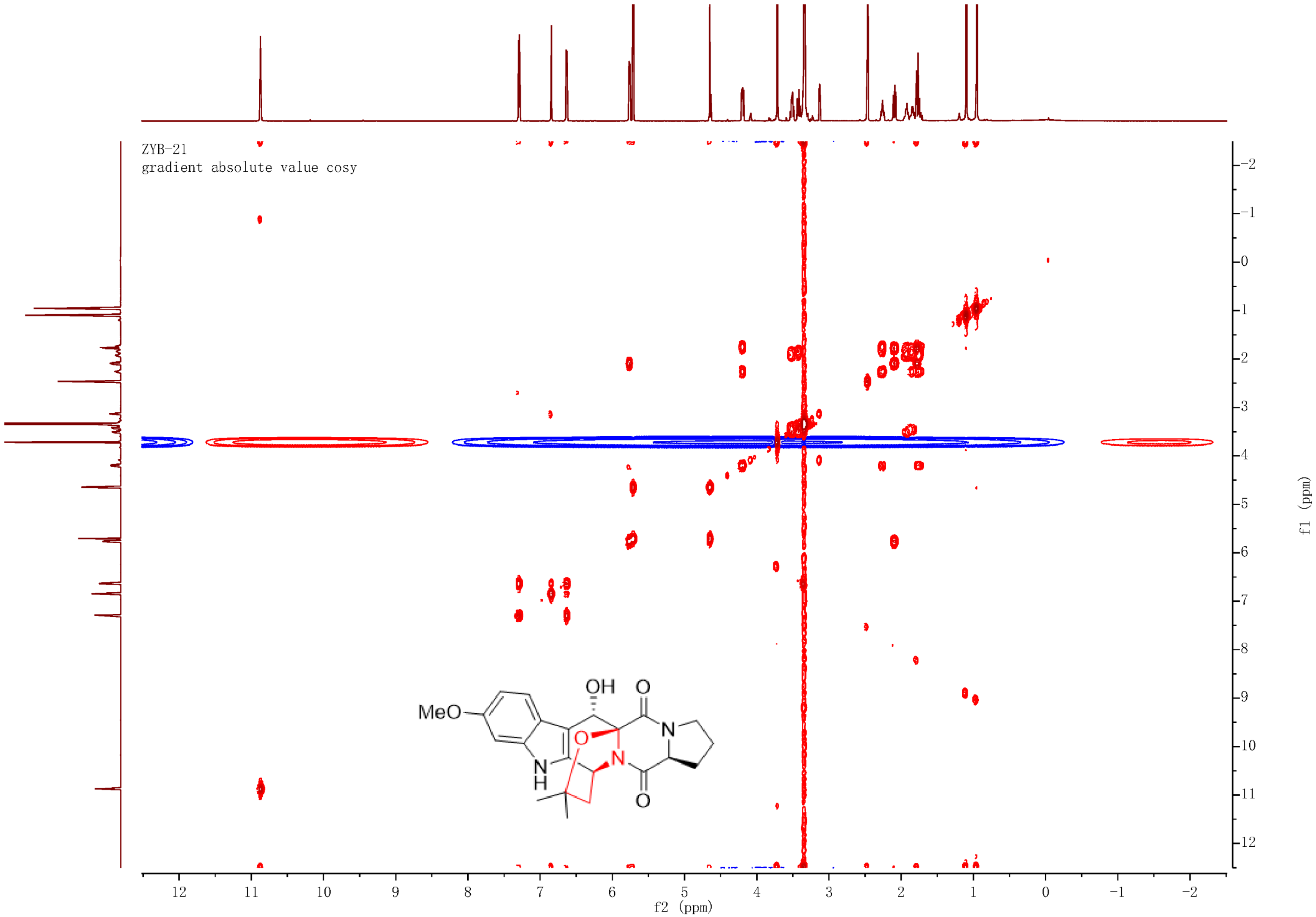

Figure S4. ^{1}H-^{1}H COSY spectrum of **1** in DMSO-d_6.

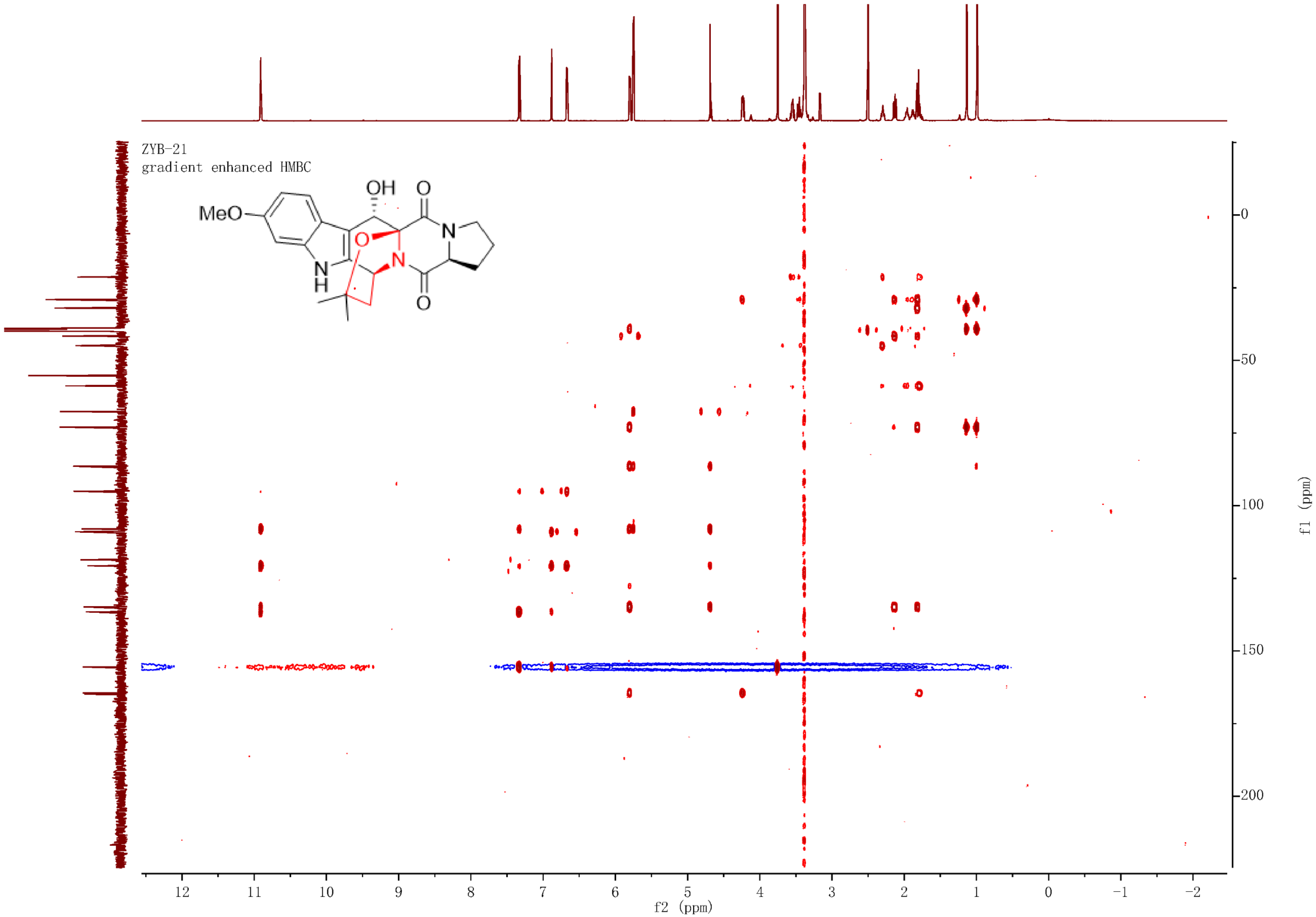

Figure S5. HMBC spectrum of **1** in DMSO-d_6.

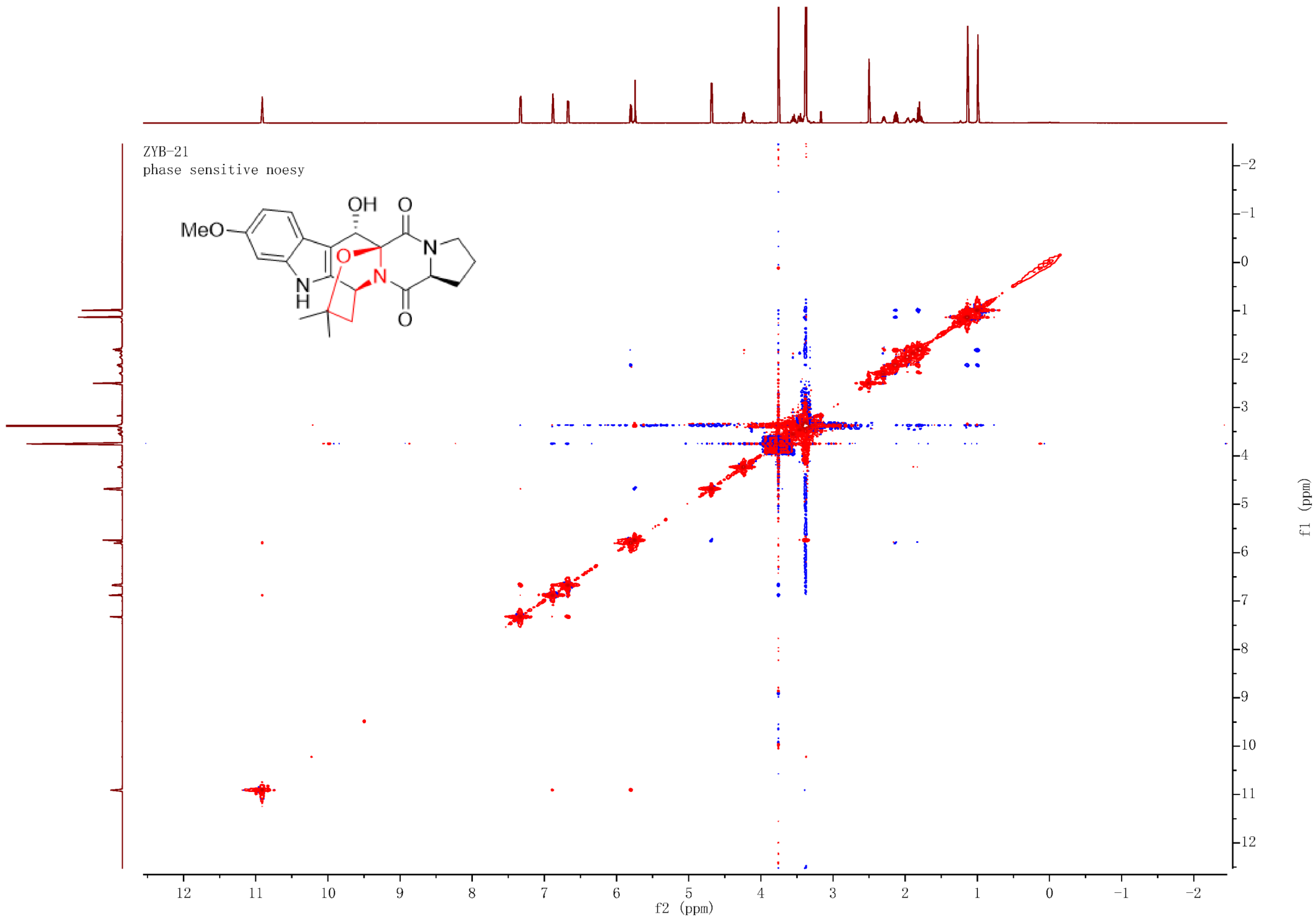

Figure S6. NOESY spectrum of **1** in DMSO-d_6.

Elemental Composition Report

Single Mass Analysis

Tolerance = 5.0 mDa / DBE: min = -1.5, max = 50.0
Element prediction: Off
Number of isotope peaks used for i-FIT = 3

Monoisotopic Mass, Even Electron Ions
422 formula(e) evaluated with 3 results within limits (up to 50 best isotopic matches for each mass)
Elements Used:
C: 20-30 H: 0-35 N: 0-5 O: 0-20 Na: 0-1
ZYB-21 70 (0.290) Cm (70:78)
1: TOF MS ES+

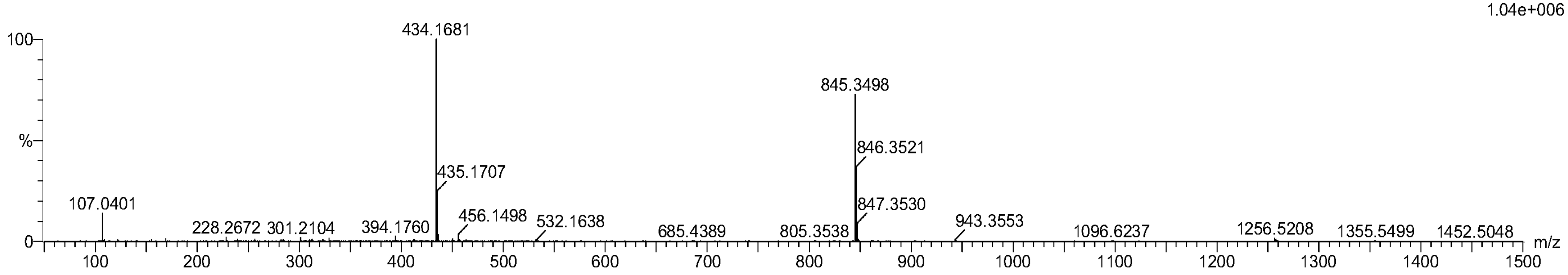

Minimum:				-1.5				
Maximum:		5.0	10.0	50.0				
Mass	Calc. Mass	mDa	PPM	DBE	i-FIT	Norm	Conf(%)	Formula
434.1681	434.1692	-1.1	-2.5	11.5	1052.2	0.054	94.71	C22 H25 N3 O5 Na
	434.1716	-3.5	-8.1	14.5	1055.1	2.993	5.01	C24 H24 N3 O5
	434.1633	4.8	11.1	20.5	1058.0	5.909	0.27	C29 H21 N3 Na

Figure S7. HR-ESI-MS spectrum of **1**.

Anton Paar GmbH
Anton Paar Strasse 10
8054 Graz
Austria

Anton Paar Polarimeter - Measurement(s)

MCP 100
Software Version: 1.50.4098.87
Serial Number: 99032784

Unique Id 303

► Sample Name:	ZYB-21
► Date:	02/01/2024 - 08:21 PM
► Username:	Administrator
► Sample State:	Ok
► Measurement Mode:	Specific Rotation
► Measurement Result:	-12.000 °
► Concentration:	0.200 g/100ml
► Optical Rotation:	-0.024 °
► Set Temperature:	25.0 °C
► Temperature:	25.0 °C

Thursday, February 01, 2024

Page 1 of 1

Figure S8. Specific rotation data of **1**.

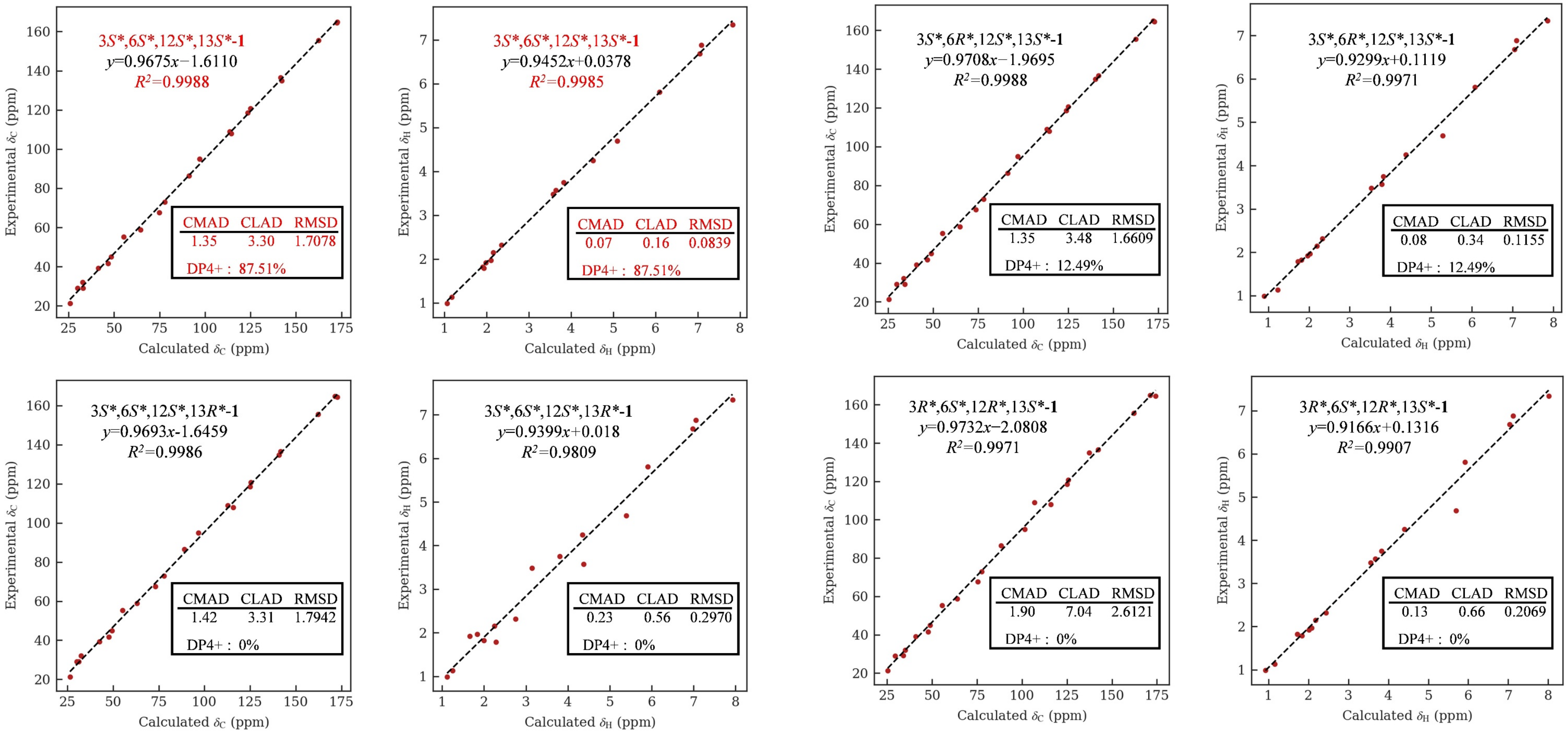

Figure S9. Regression analysis of experimental versus calculated ^{1}H, ^{13}C NMR chemical shifts of **1**.

Functional	Solvent?		Basis Set	
mPW1PW91	PCM		6-31G(d)	
	Isomer 1	Isomer 2	Isomer 3	Isomer 4
sDP4+ (H data)	88.13%	0.00%	11.86%	0.01%
sDP4+ (C data)	38.54%	10.43%	51.03%	0.00%
sDP4+ (all data)	84.88%	0.00%	15.12%	0.00%
uDP4+ (H data)	32.55%	0.00%	65.97%	1.47%
uDP4+ (C data)	5.75%	56.99%	2.27%	35.00%
uDP4+ (all data)	48.15%	0.00%	38.57%	13.28%
DP4+ (H data)	78.57%	0.00%	21.43%	0.00%
DP4+ (C data)	23.77%	63.79%	12.44%	0.01%
DP4+ (all data)	87.51%	0.00%	12.49%	0.00%

Figure S1. The DP4+ probability analysis of **1**.

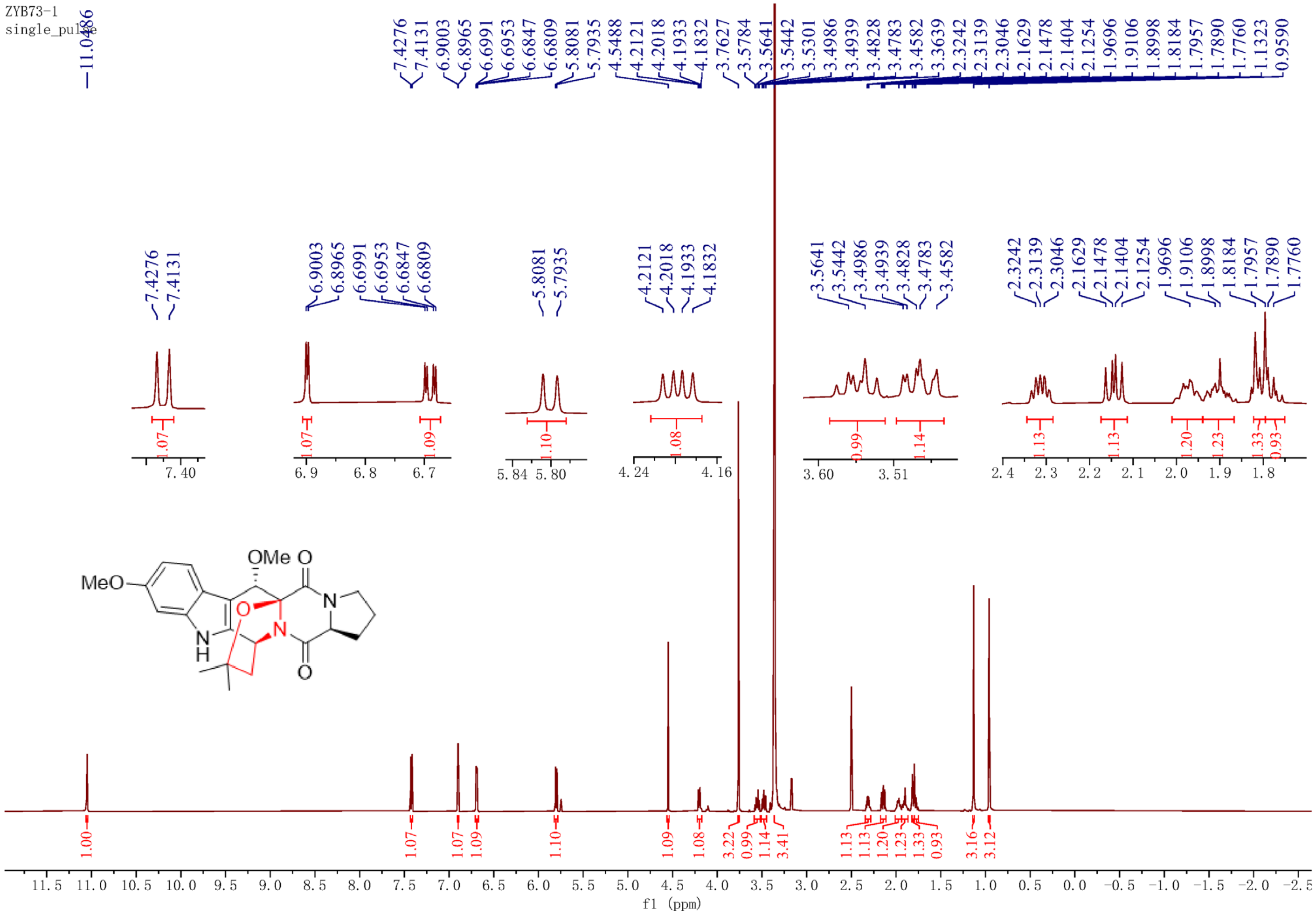

Figure S11. ^{1}H NMR spectrum of **2** in DMSO-d_6.

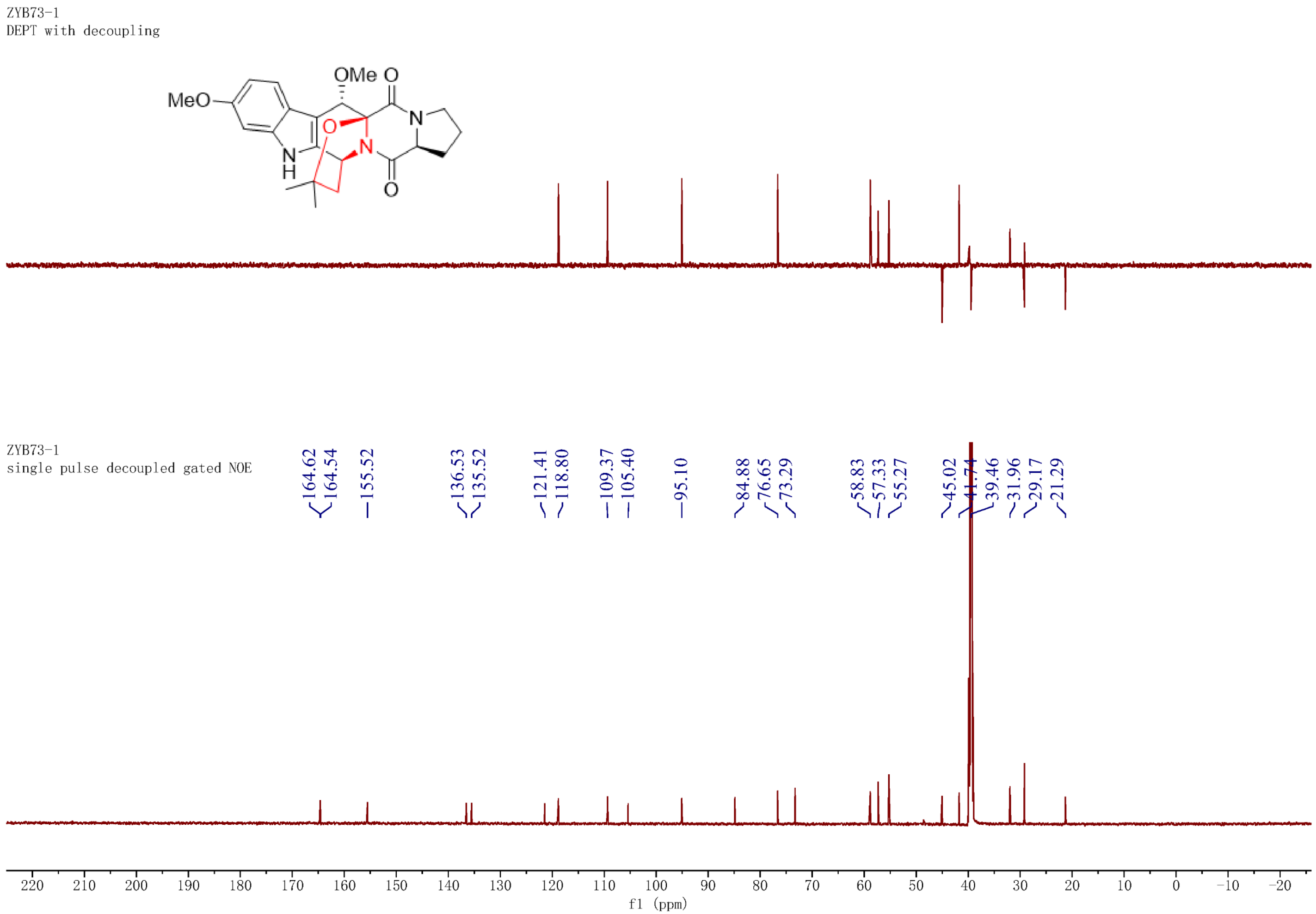

Figure S12. ^{13}C NMR spectrum of **2** in DMSO-d_6.

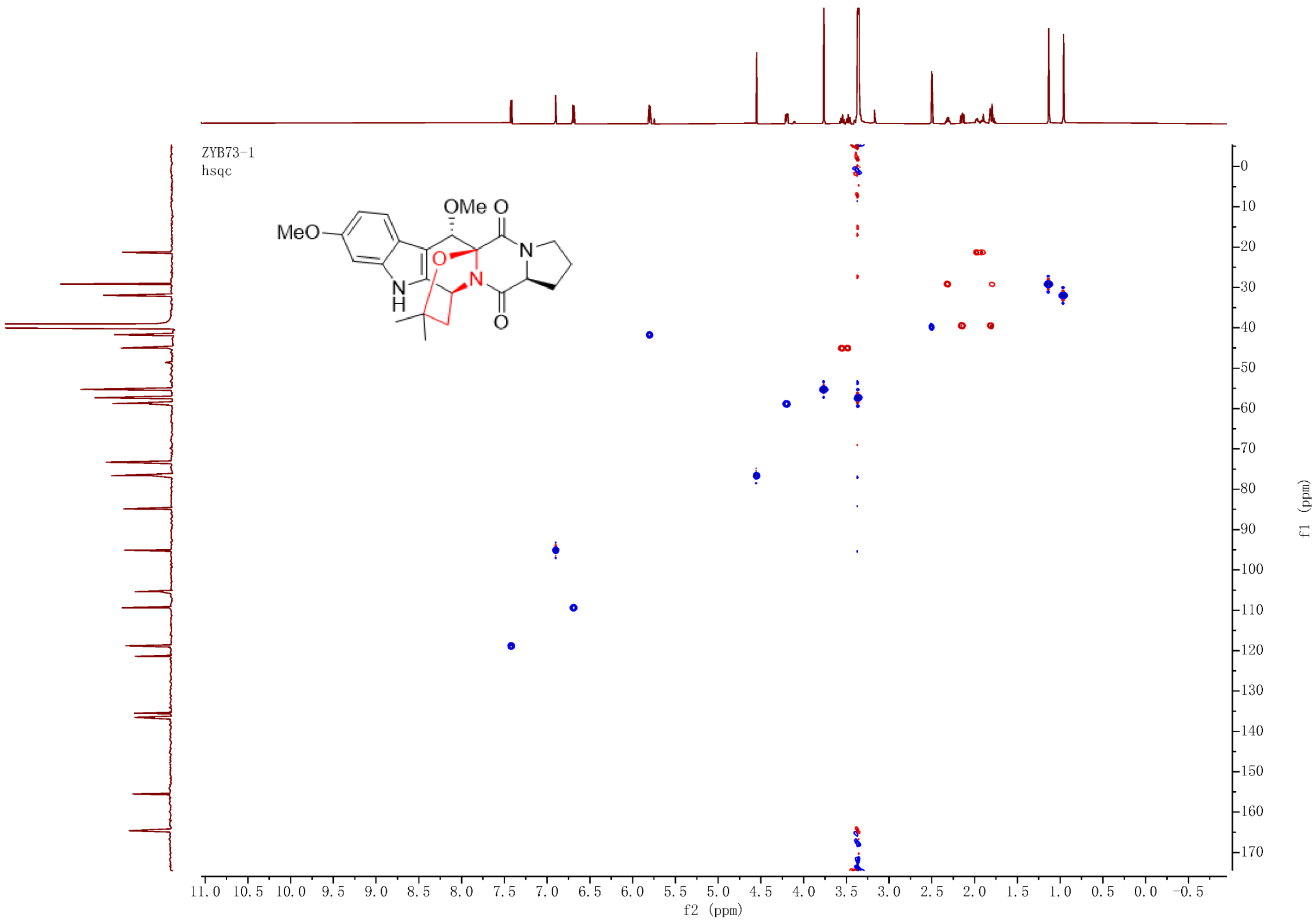

Figure S13. HSQC spectrum of **2** in DMSO-d_6.

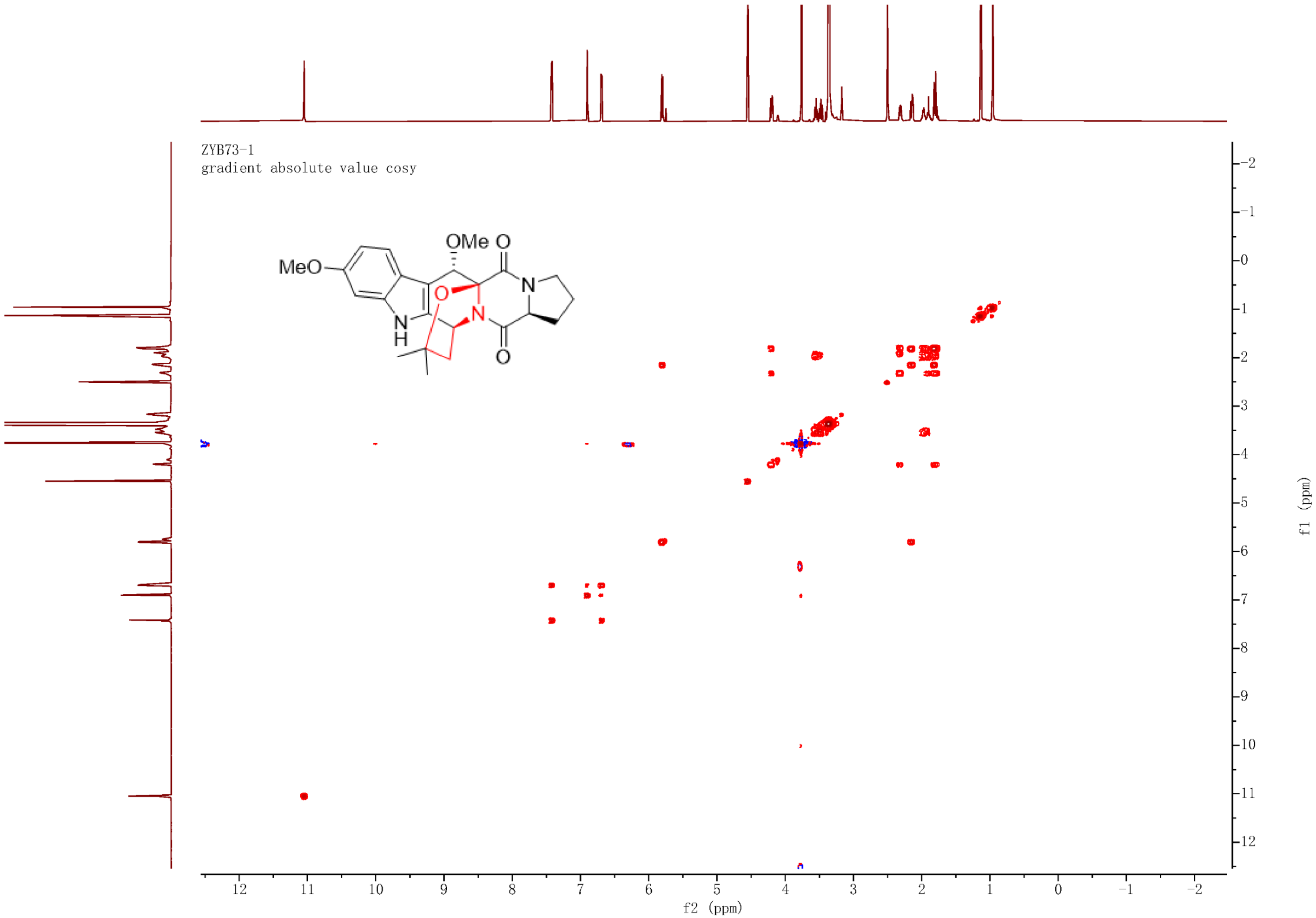

Figure S14. ^{1}H-^{1}H COSY spectrum of **2** in DMSO-d_6.

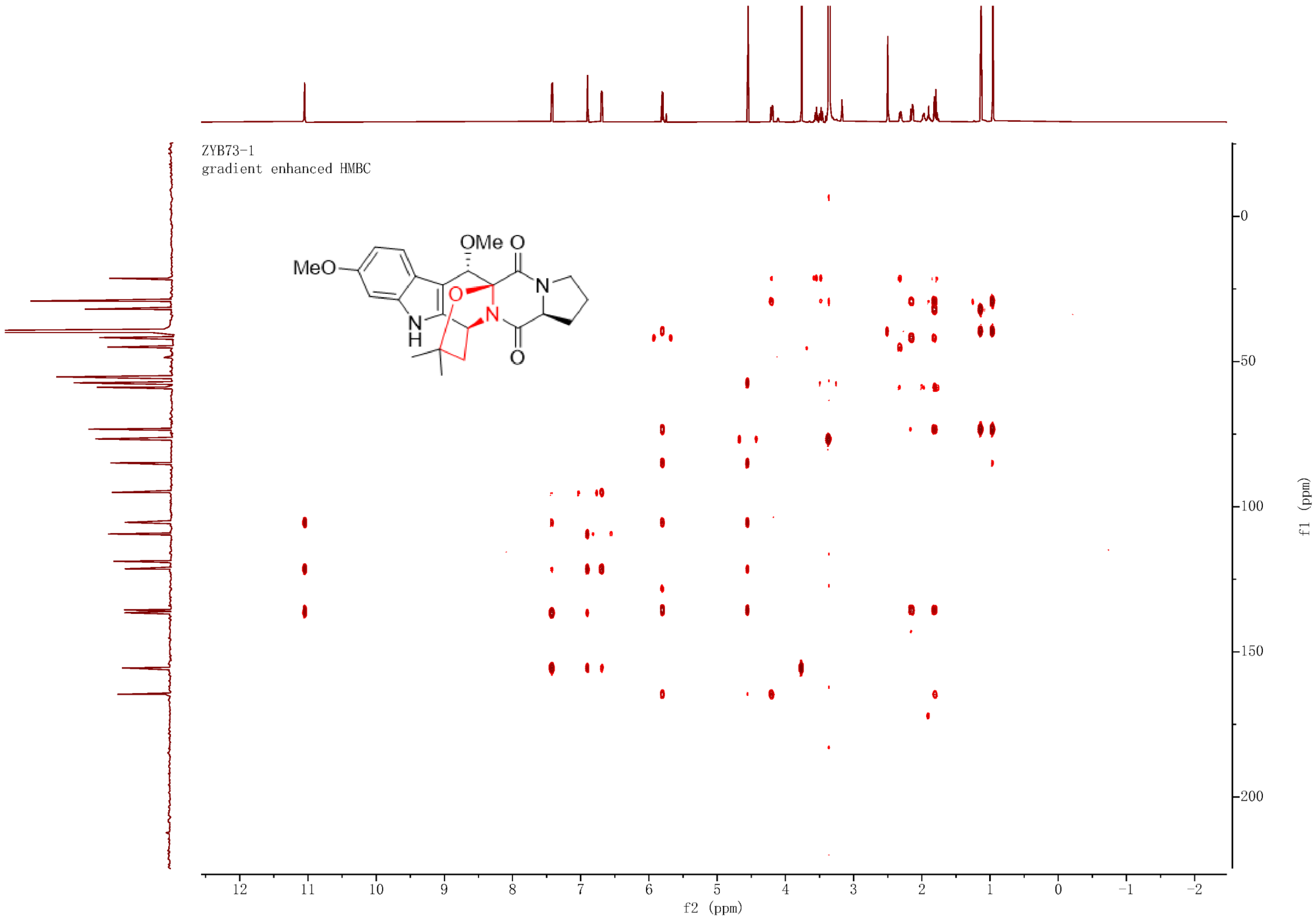

Figure S15. HMBC spectrum of **2** in DMSO-d_6.

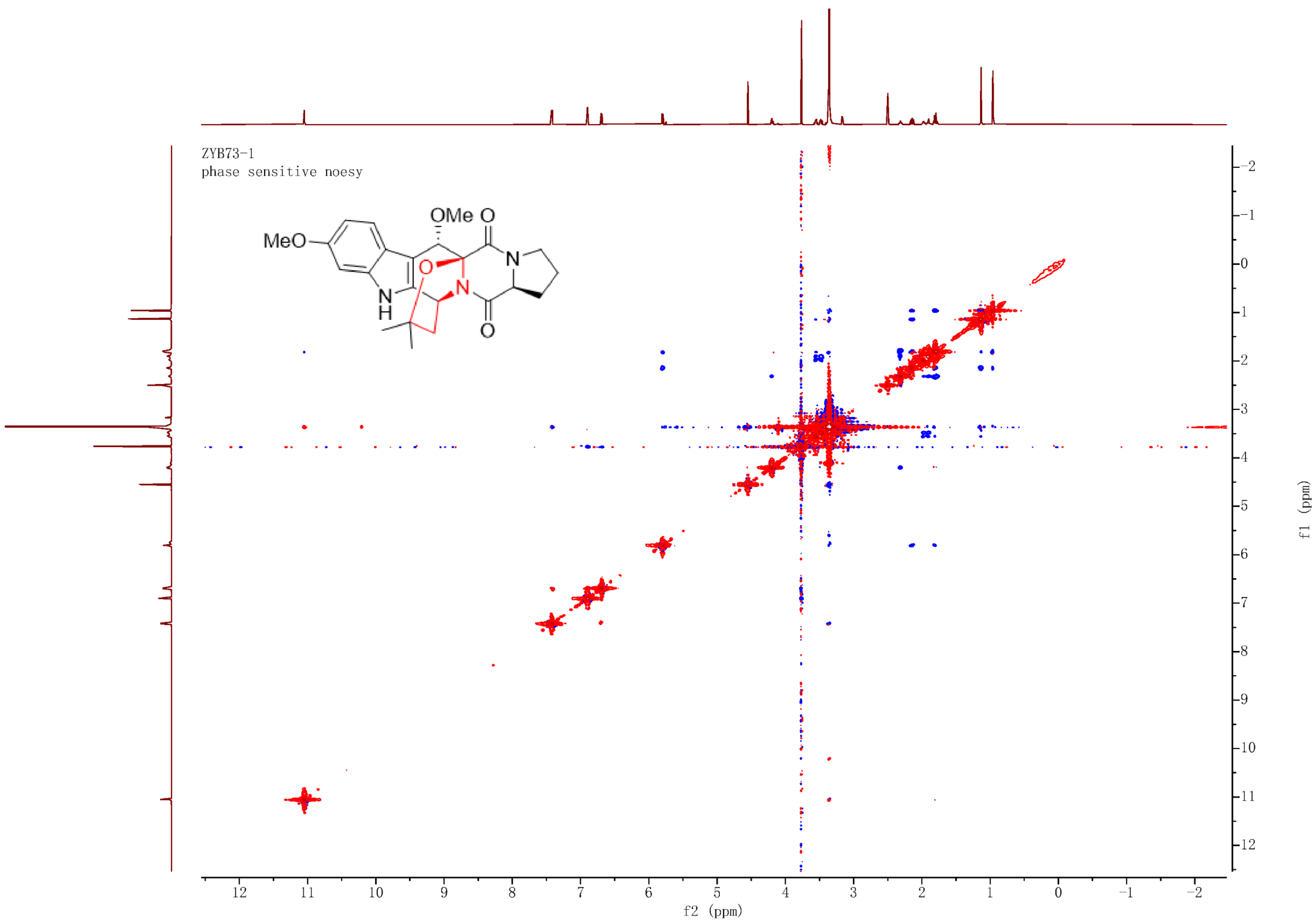

Figure S16. NOESY spectrum of **2** in DMSO-*d*6.

Elemental Composition Report

Single Mass Analysis

Tolerance = 5.0 mDa / DBE: min = -1.5, max = 50.0
Element prediction: Off
Number of isotope peaks used for i-FIT = 3

Monoisotopic Mass, Even Electron Ions
459 formula(e) evaluated with 6 results within limits (up to 50 best isotopic matches for each mass)
Elements Used:
C: 18-30 H: 0-35 N: 0-5 O: 0-20 Na: 0-1
ZYB-73-1-April 80 (0.526) Cm (76:84)
1: TOF MS ES+

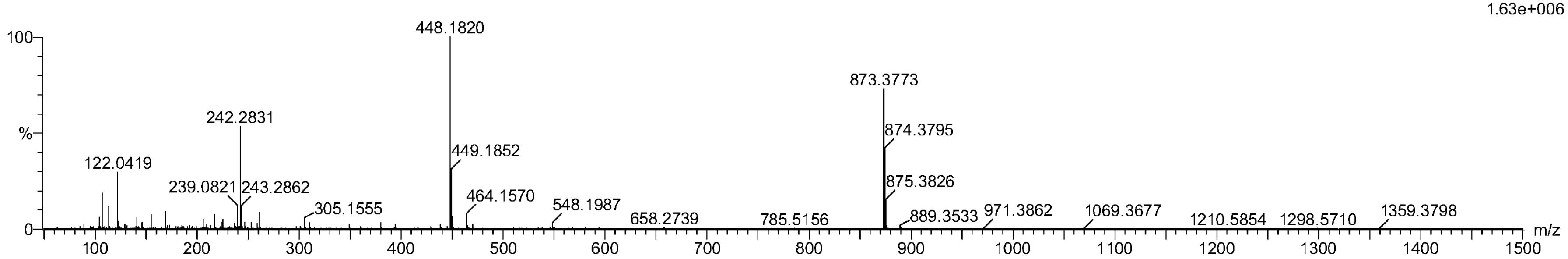

Minimum:				-1.5				
Maximum:		5.0	10.0	50.0				
Mass	Calc. Mass	mDa	PPM	DBE	i-FIT	Norm	Conf(%)	Formula
448.1820	448.1819	0.1	0.2	5.5	1751.3	2.912	5.44	C19 H30 N O11
	448.1832	-1.2	-2.7	10.5	1750.9	2.457	8.57	C20 H26 N5 O7
	448.1808	1.2	2.7	7.5	1752.2	3.775	2.29	C18 H27 N5 O7 Na
	448.1848	-2.8	-6.2	11.5	1751.0	2.601	7.42	C23 H27 N3 O5 Na
	448.1790	3.0	6.7	20.5	1749.3	0.902	40.59	C30 H23 N3 Na
	448.1773	4.7	10.5	19.5	1749.4	1.030	35.69	C27 H22 N5 O2

Figure S17. HR-ESI-MS spectrum of **2**.

Anton Paar GmbH
Anton Paar Strasse 10
8054 Graz
Austria

Anton Paar

Anton Paar Polarimeter - Measurement(s)

MCP 100
Software Version: 1.50.4098.87
Serial Number: 99032784

Unique Id 309

- Sample Name: ZYB-73-1
- Date: 02/01/2024 - 08:42 PM
- Username: Administrator
- Sample State: Ok

- Measurement Mode: Specific Rotation
- Measurement Result: -24.000 °
- Concentration: 0.100 g/100ml
- Optical Rotation: -0.025 °
- Set Temperature: 25.0 °C
- Temperature: 25.0 °C

Thursday, February 01, 2024 Page 1 of 1

Figure S18. Specific rotation data of **2**.

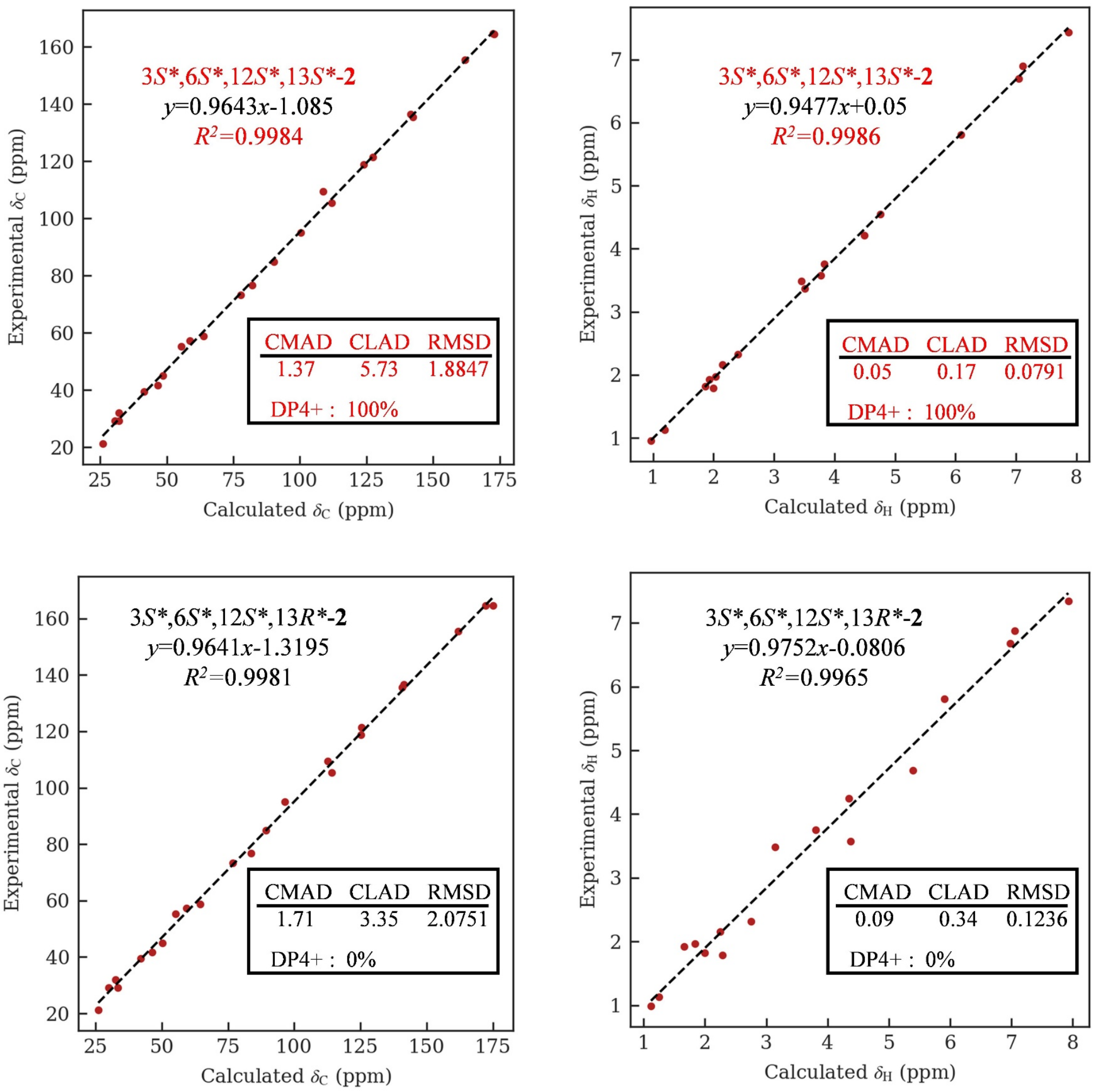

Figure S19. Regression analysis of experimental versus calculated ^1H, ^{13}C NMR chemical shifts of **2**.

Functional	Solvent?	Basis Set	
mPW1PW91	PCM	6-31G(d)	

	Isomer 1	Isomer 2	Isomer 3	Isomer 4
sDP4+ (H data)	99.57%	0.43%	–	–
sDP4+ (C data)	99.95%	0.05%	–	–
sDP4+ (all data)	100.00%	0.00%	–	–
uDP4+ (H data)	98.31%	1.69%	–	–
uDP4+ (C data)	98.68%	1.32%	–	–
uDP4+ (all data)	99.98%	0.02%	–	–
DP4+ (H data)	99.99%	0.01%	–	–
DP4+ (C data)	100.00%	0.00%	–	–
DP4+ (all data)	100.00%	0.00%	–	–

Figure S20. The DP4+ probability analysis of **2**.

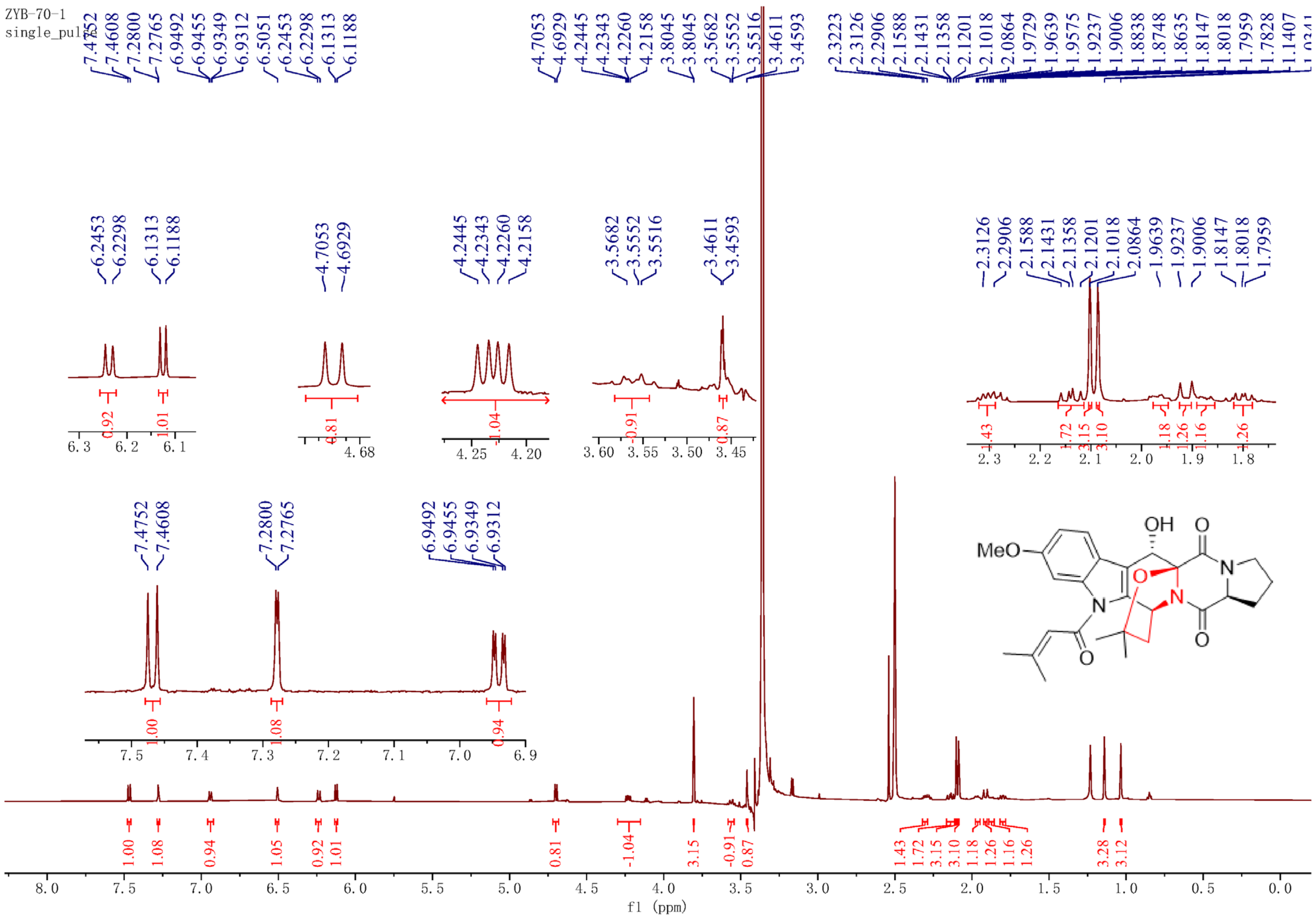

Figure S21. ^{1}H NMR spectrum of **3** in DMSO-d_6.

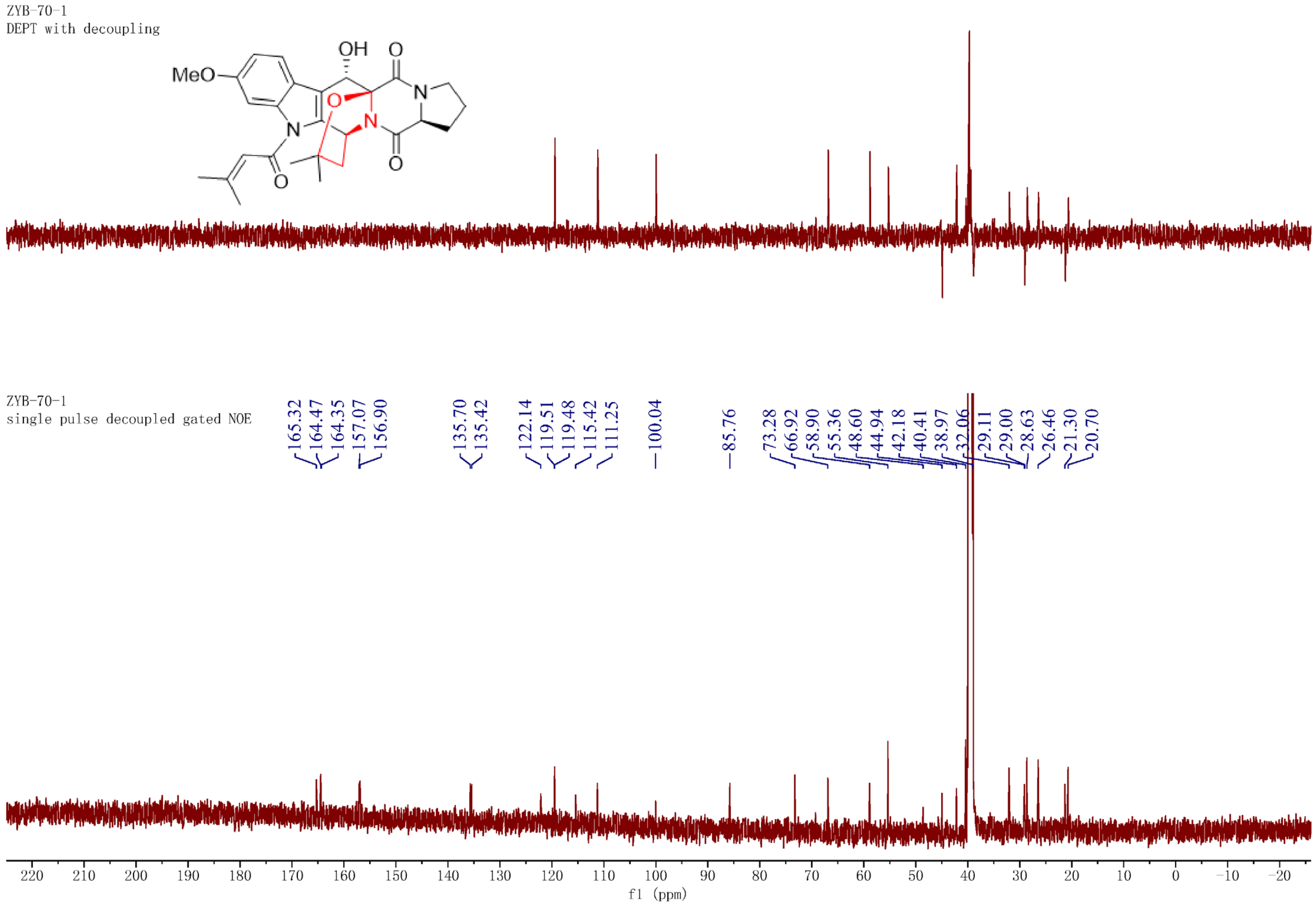

Figure S2. ^{13}C NMR spectrum of **3** in DMSO-d_6.

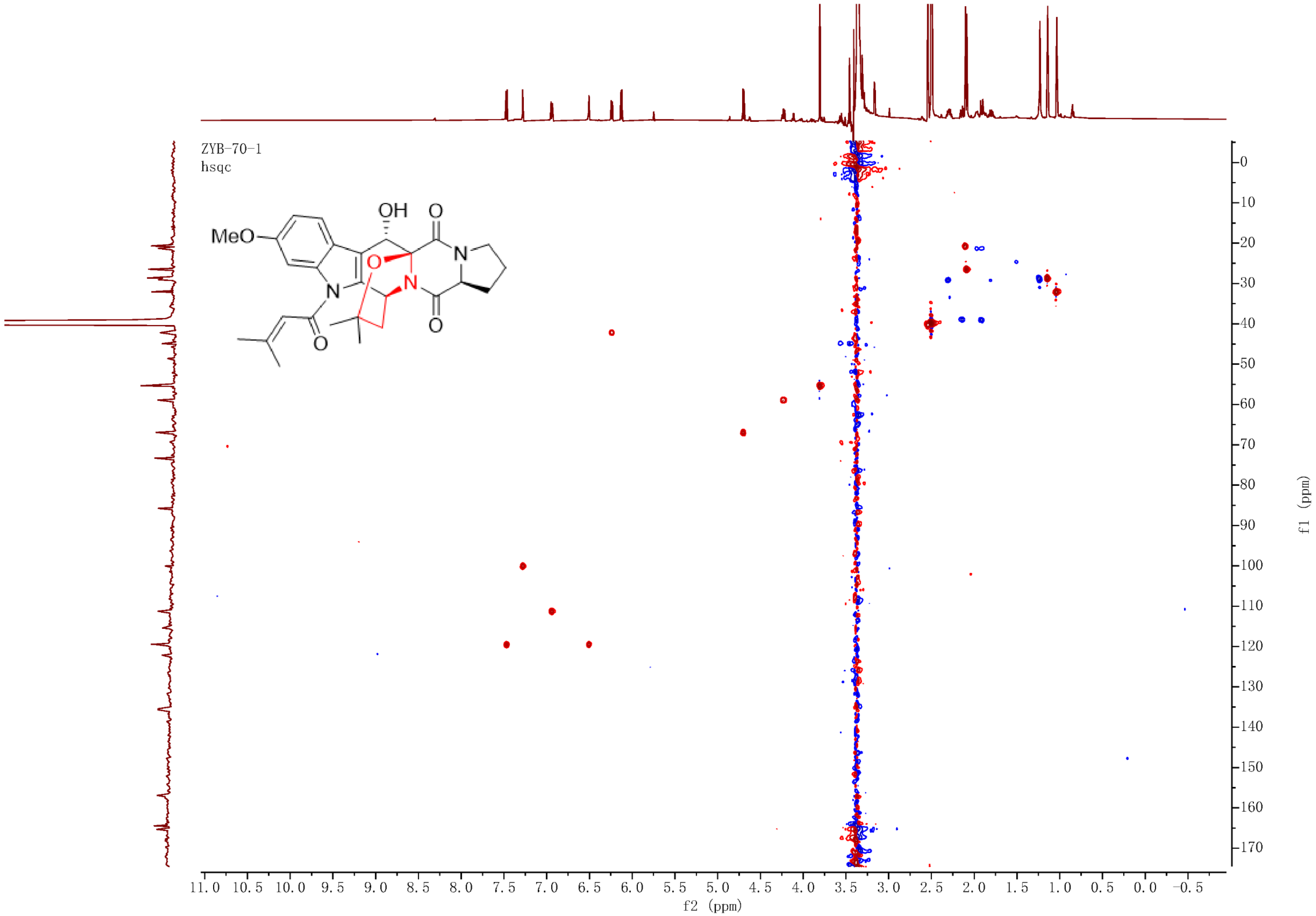

Figure S23. HSQC spectrum of **3** in DMSO-d_6.

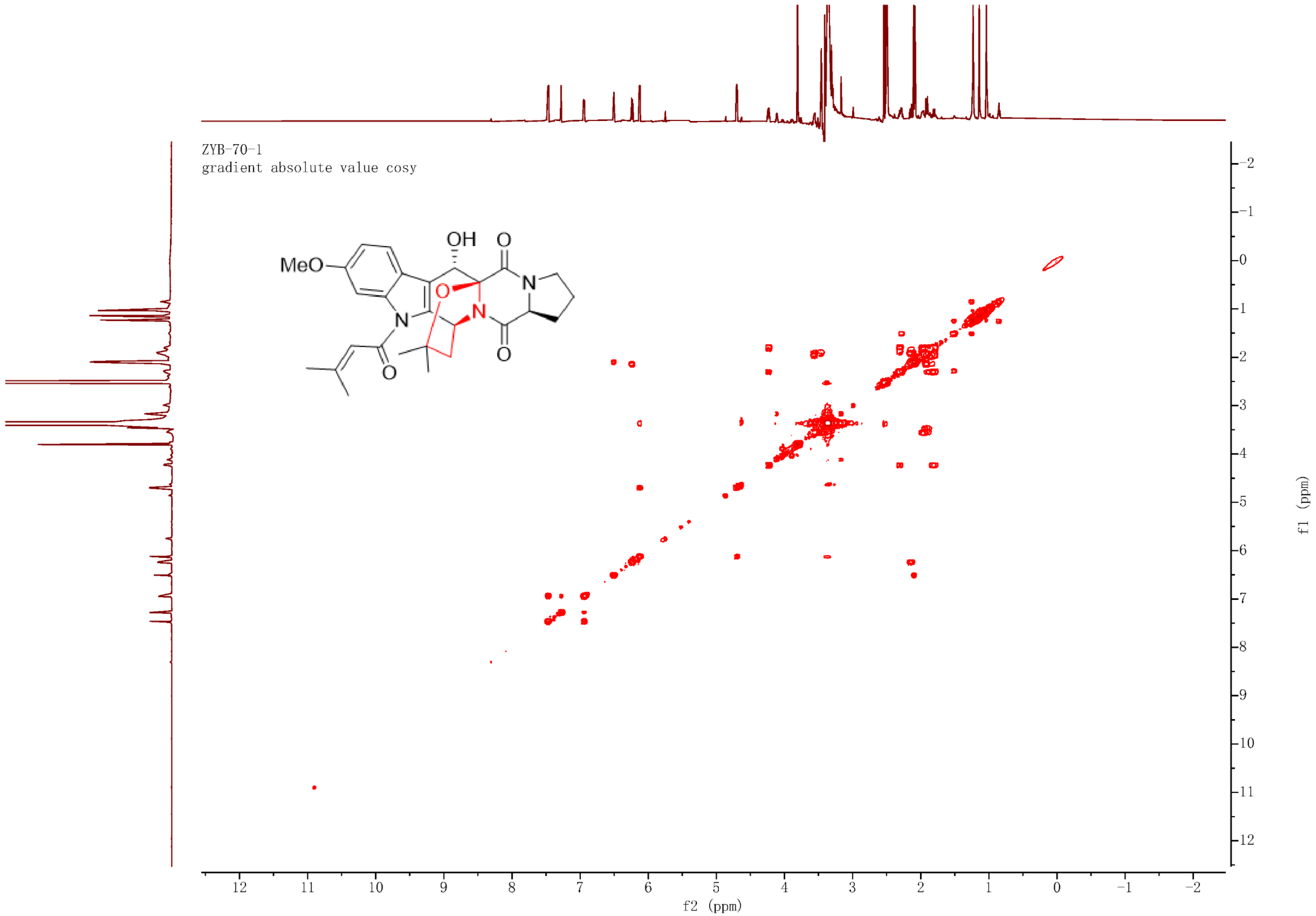

Figure S24. ^1H-^1H COSY spectrum of **3** in DMSO-d_6.

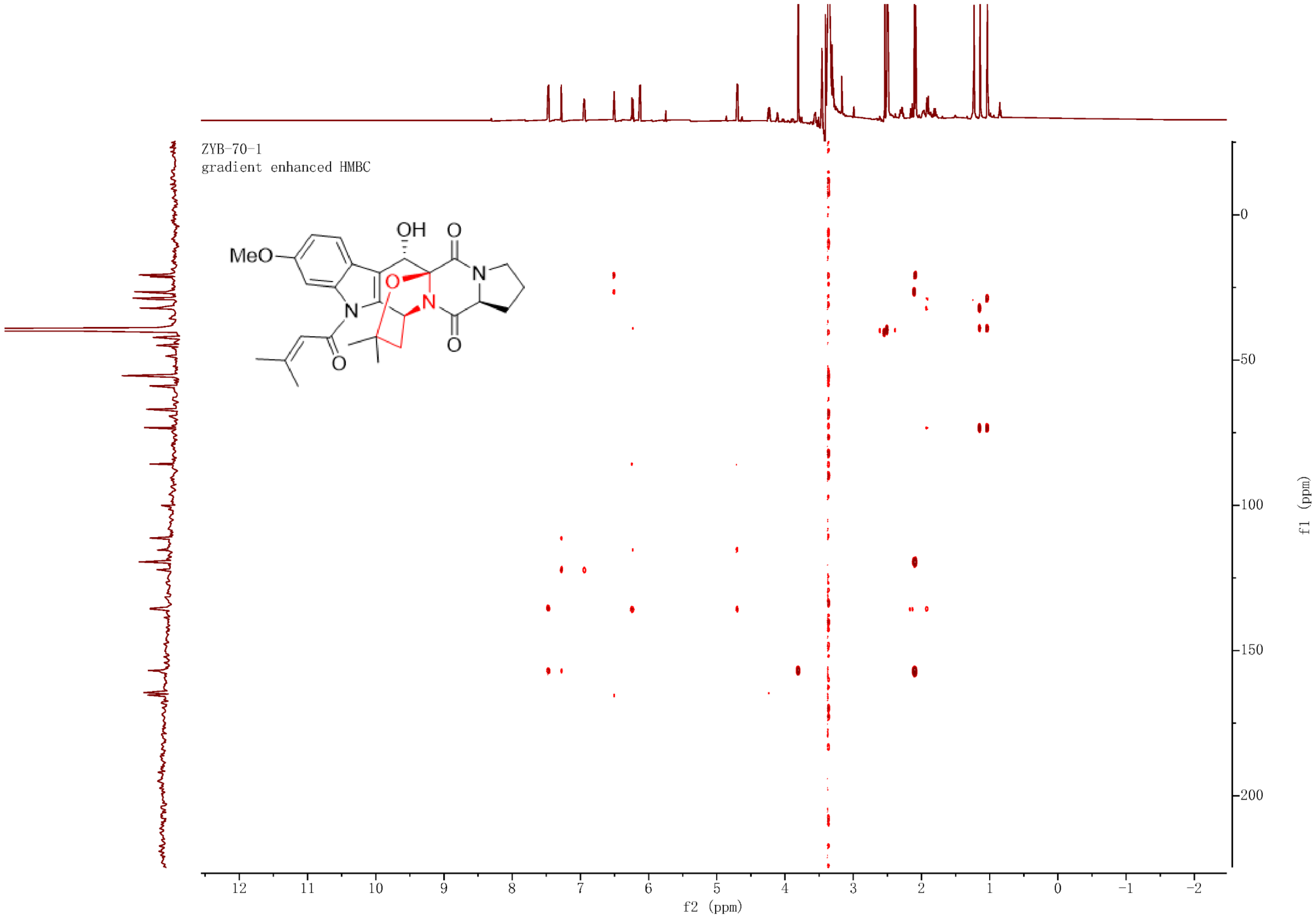

Figure S25. HMBC spectrum of **3** in DMSO-d_6.

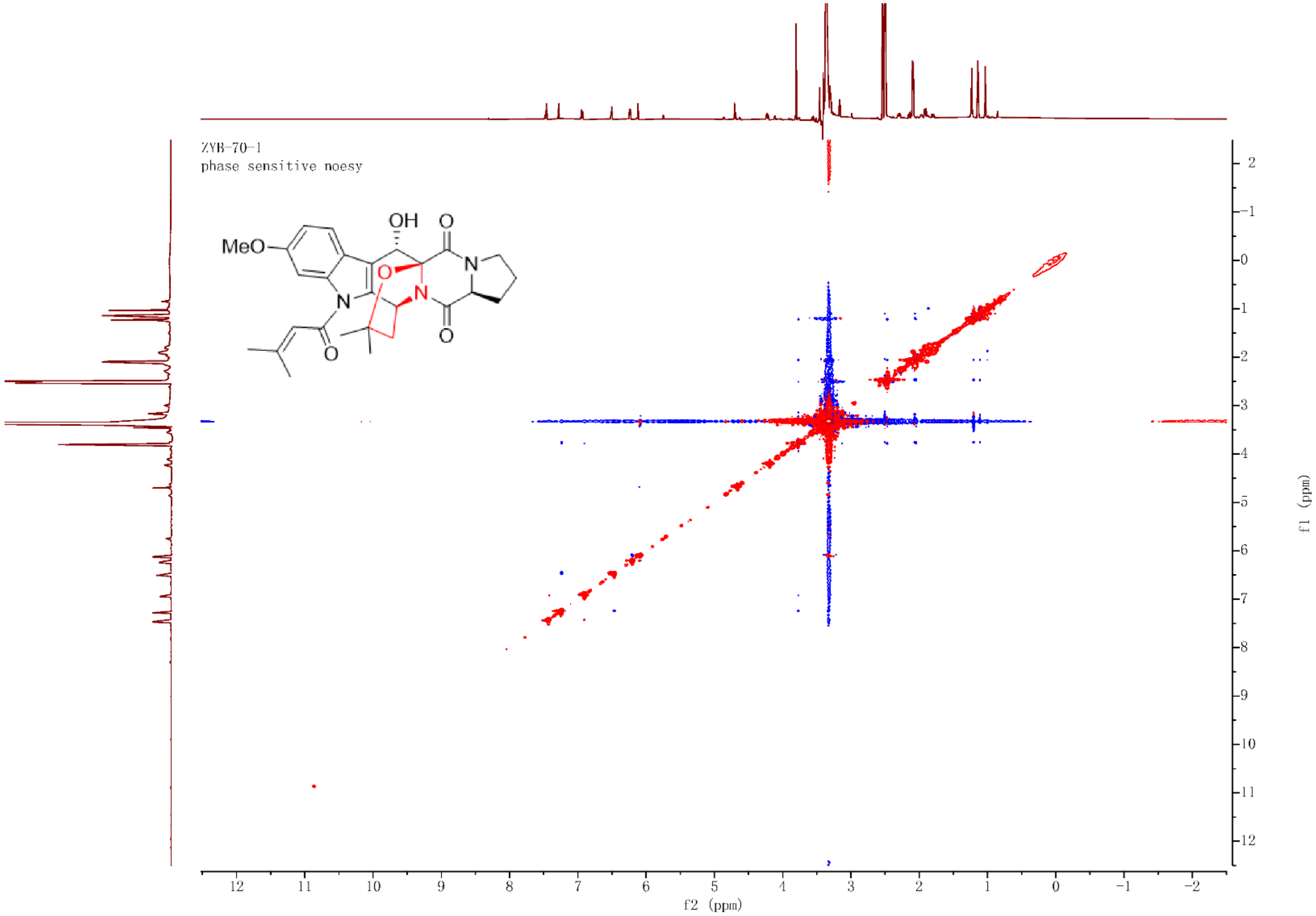

Figure S26. NOESY spectrum of **3** in DMSO-*d*6.

Elemental Composition Report **Page 1**

Single Mass Analysis
Tolerance = 5.0 mDa / DBE: min = -1.5, max = 50.0
Element prediction: Off
Number of isotope peaks used for i-FIT = 3

Monoisotopic Mass, Even Electron Ions
406 formula(e) evaluated with 6 results within limits (up to 50 best isotopic matches for each mass)
Elements Used:
C: 20-30 H: 0-35 N: 0-5 O: 0-20 Na: 0-1
ZYB-70-1-April 41 (0.272) Cm (36:54)
1: TOF MS ES+

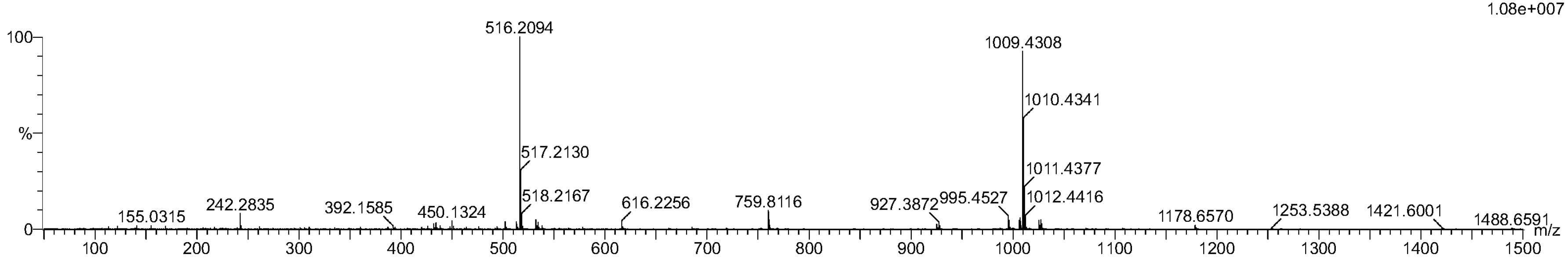

Minimum:				-1.5				
Maximum:		5.0	10.0	50.0				
Mass	Calc. Mass	mDa	PPM	DBE	i-FIT	Norm	Conf(%)	Formula
516.2094	516.2111	-1.7	-3.3	13.5	1899.4	0.758	46.87	C27 H31 N3 O6 Na
	516.2135	-4.1	-7.9	16.5	1899.7	1.060	34.64	C29 H30 N3 O6
	516.2094	0.0	0.0	12.5	1900.8	2.190	11.19	C24 H30 N5 O8
	516.2081	1.3	2.5	7.5	1901.5	2.909	5.45	C23 H34 N O12
	516.2070	2.4	4.6	9.5	1902.9	4.308	1.35	C22 H31 N5 O8 Na
	516.2057	3.7	7.2	4.5	1903.9	5.300	0.50	C21 H35 N O12 Na

Figure S27. HR-ESI-MS spectrum of **3**.

Anton Paar GmbH
Anton Paar Strasse 10
8054 Graz
Austria

Anton Paar Polarimeter - Measurement(s)

MCP 100
Software Version: 1.50.4098.87
Serial Number: 99032784

Unique Id 305

- Sample Name: ZYB-70-1
- Date: 02/01/2024 - 08:28 PM
- Username: Administrator
- Sample State: Ok

- Measurement Mode: Specific Rotation
- Measurement Result: 34.000 °
- Concentration: 0.050 g/100ml
- Optical Rotation: 0.017 °
- Set Temperature: 25.0 °C
- Temperature: 25.0 °C

Thursday, February 01, 2024 Page 1 of 1

Figure S28. Specific rotation data of **3**.

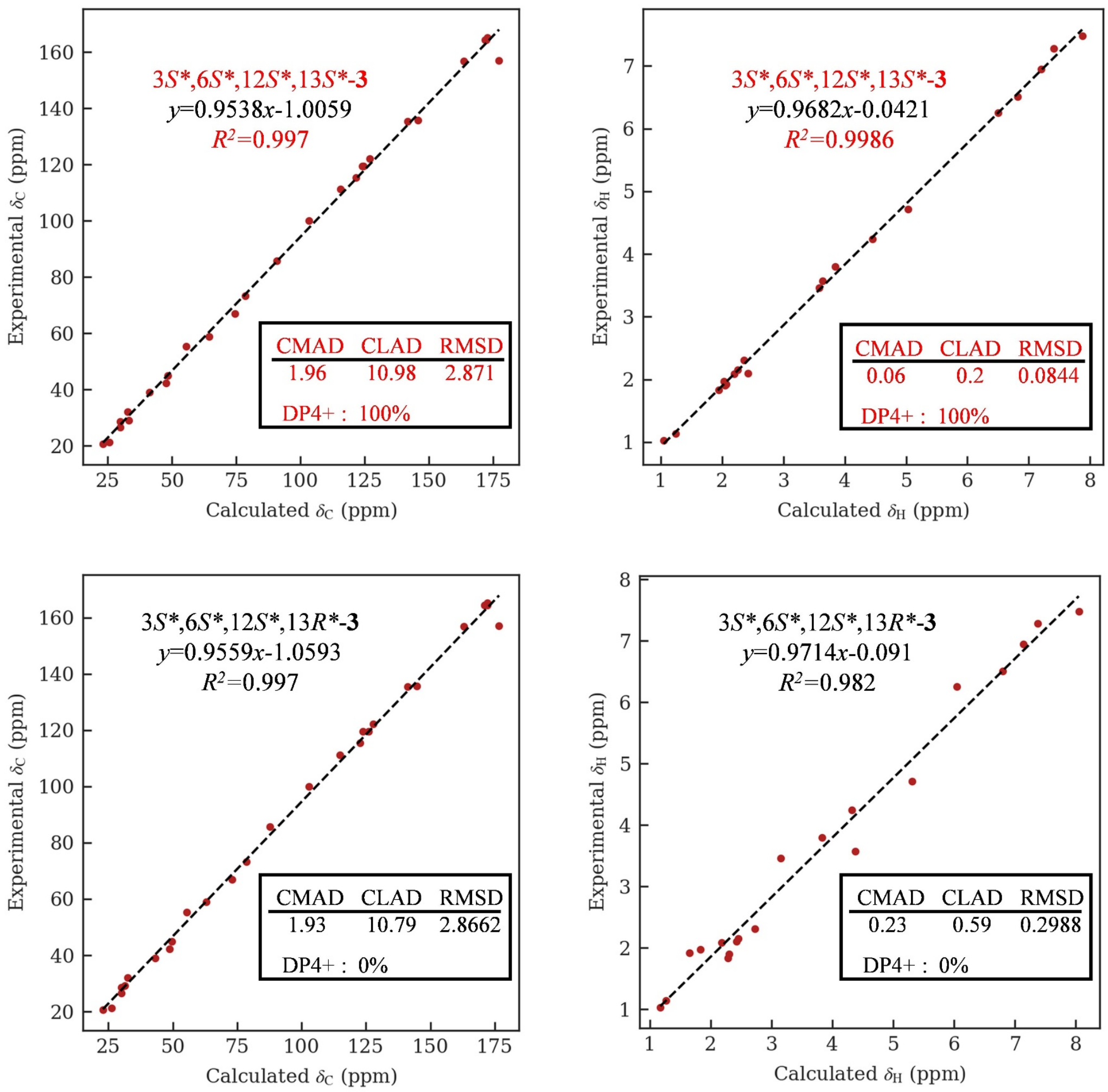

Figure S29. Regression analysis of experimental versus calculated ^{1}H, ^{13}C NMR chemical shifts of **3**.

Functional	Solvent?	Basis Set
mPW1PW91	PCM	6-31G(d)

	Isomer 1	Isomer 2	Isomer 3	Isomer 4
sDP4+ (H data)	100.00%	0.00%	–	–
sDP4+ (C data)	48.53%	51.47%	–	–
sDP4+ (all data)	100.00%	0.00%	–	–
uDP4+ (H data)	100.00%	0.00%	–	–
uDP4+ (C data)	10.56%	89.44%	–	–
uDP4+ (all data)	100.00%	0.00%	–	–
DP4+ (H data)	100.00%	0.00%	–	–
DP4+ (C data)	10.02%	89.98%	–	–
DP4+ (all data)	100.00%	0.00%	–	–

Figure S30. The DP4+ probability analysis of **3**.

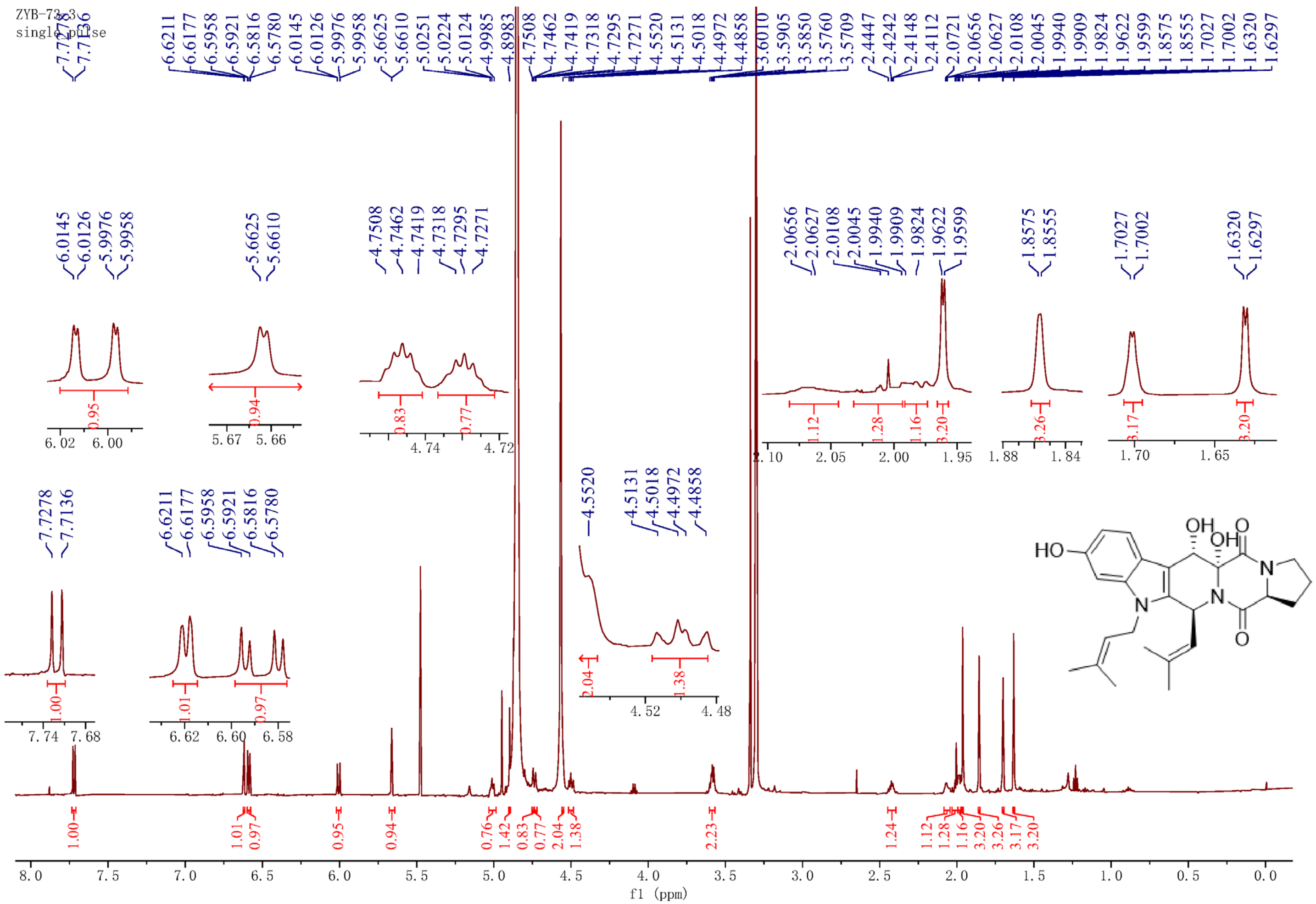

Figure S31. ^{1}H NMR spectrum of **4** in CD_3OD.

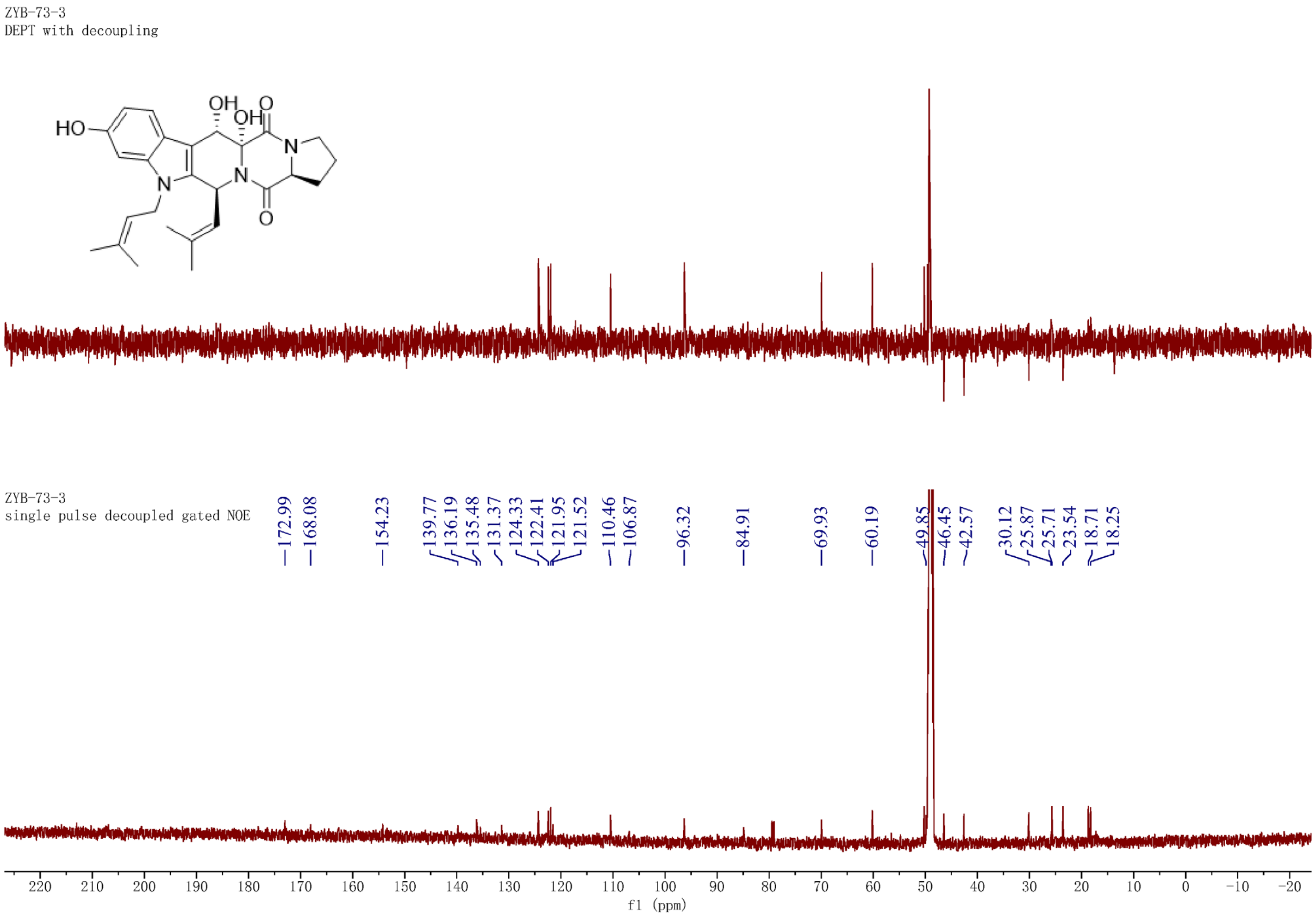

Figure S32. ^{13}C NMR spectrum of **4** in CD_3OD.

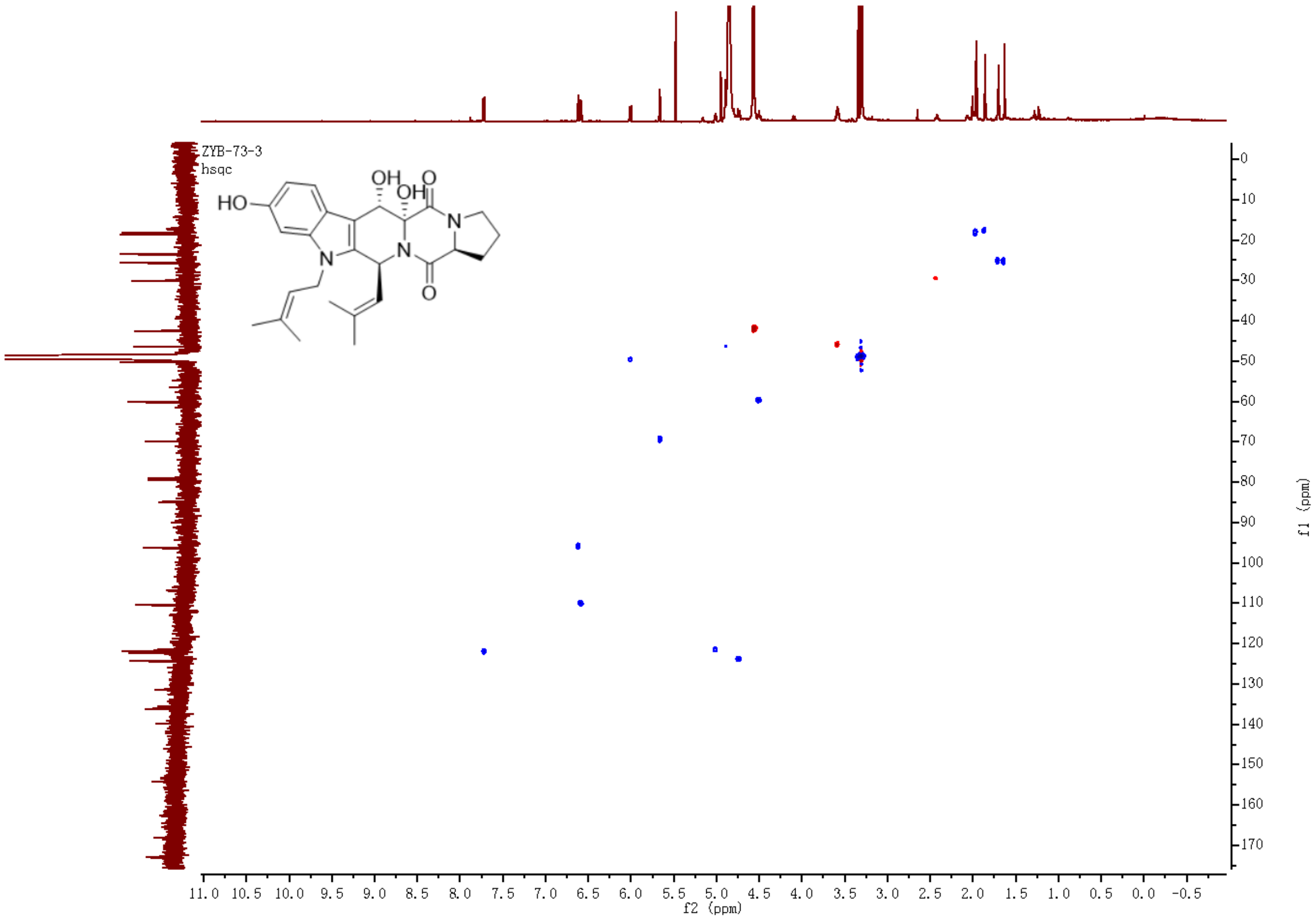

Figure S33. HSQC spectrum of **4** in CD_3OD.

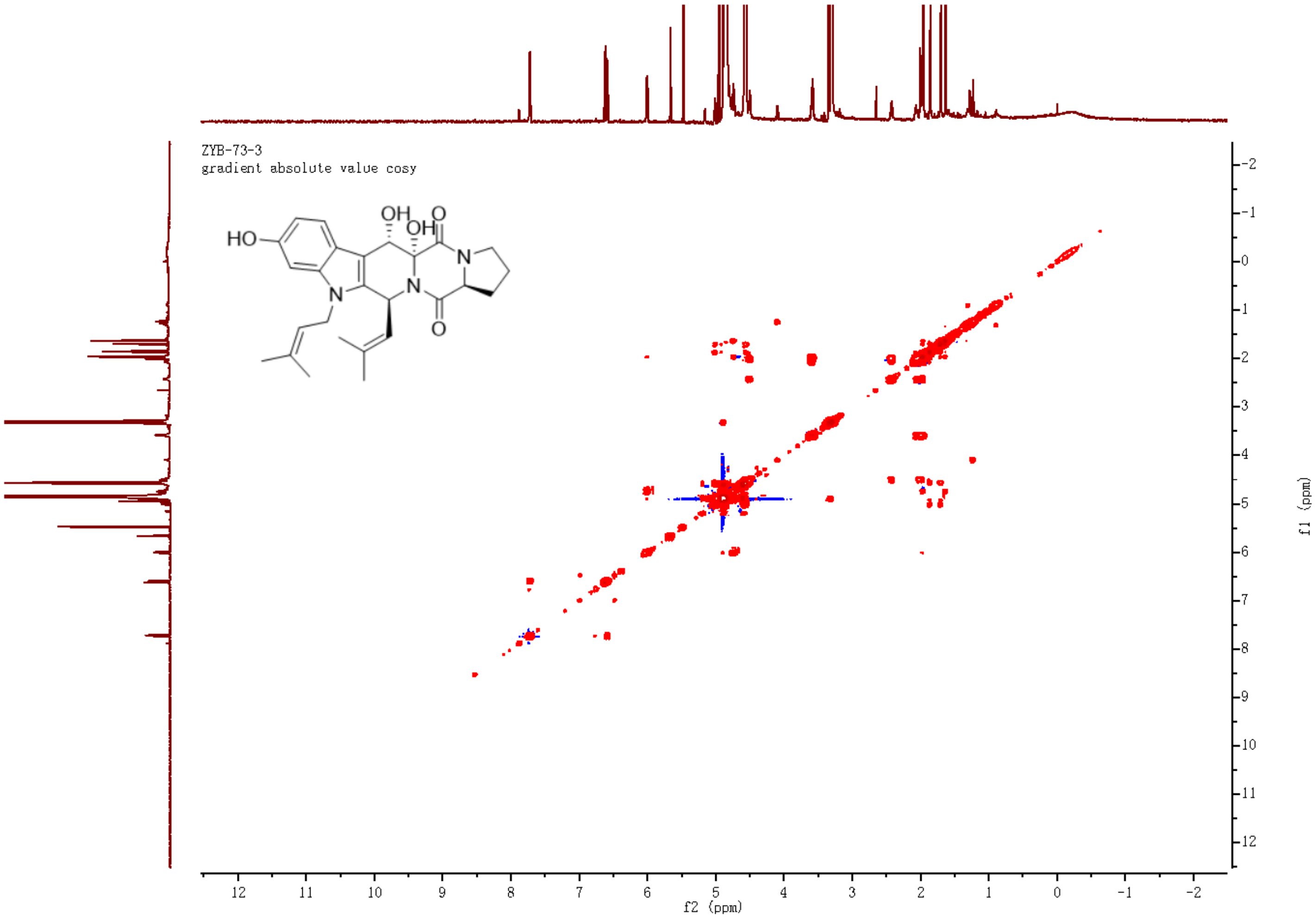

Figure S34. ^1H-^1H COSY spectrum of **4** in CD_3OD.

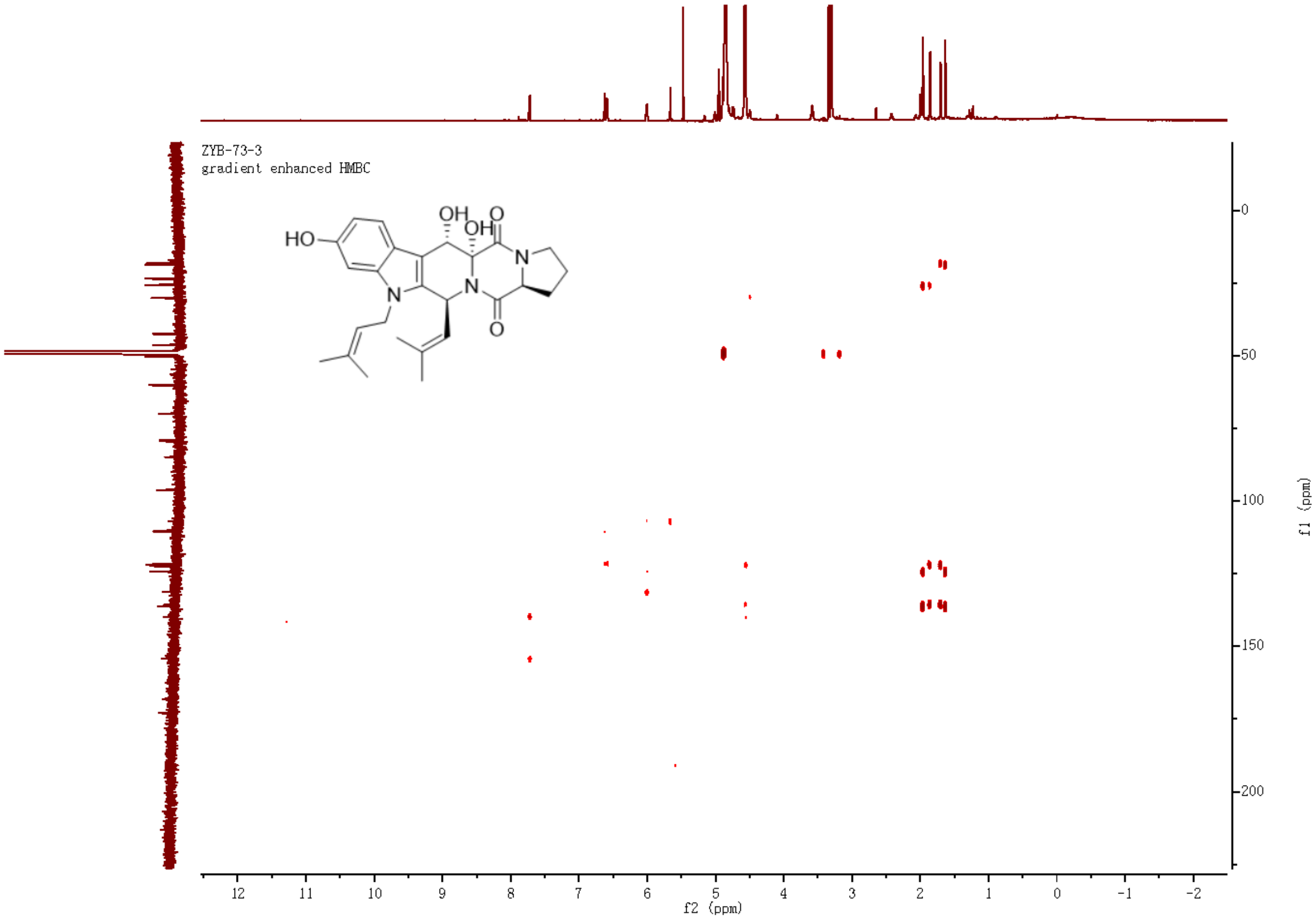

Figure S35. HMBC spectrum of **4** in CD_3OD.

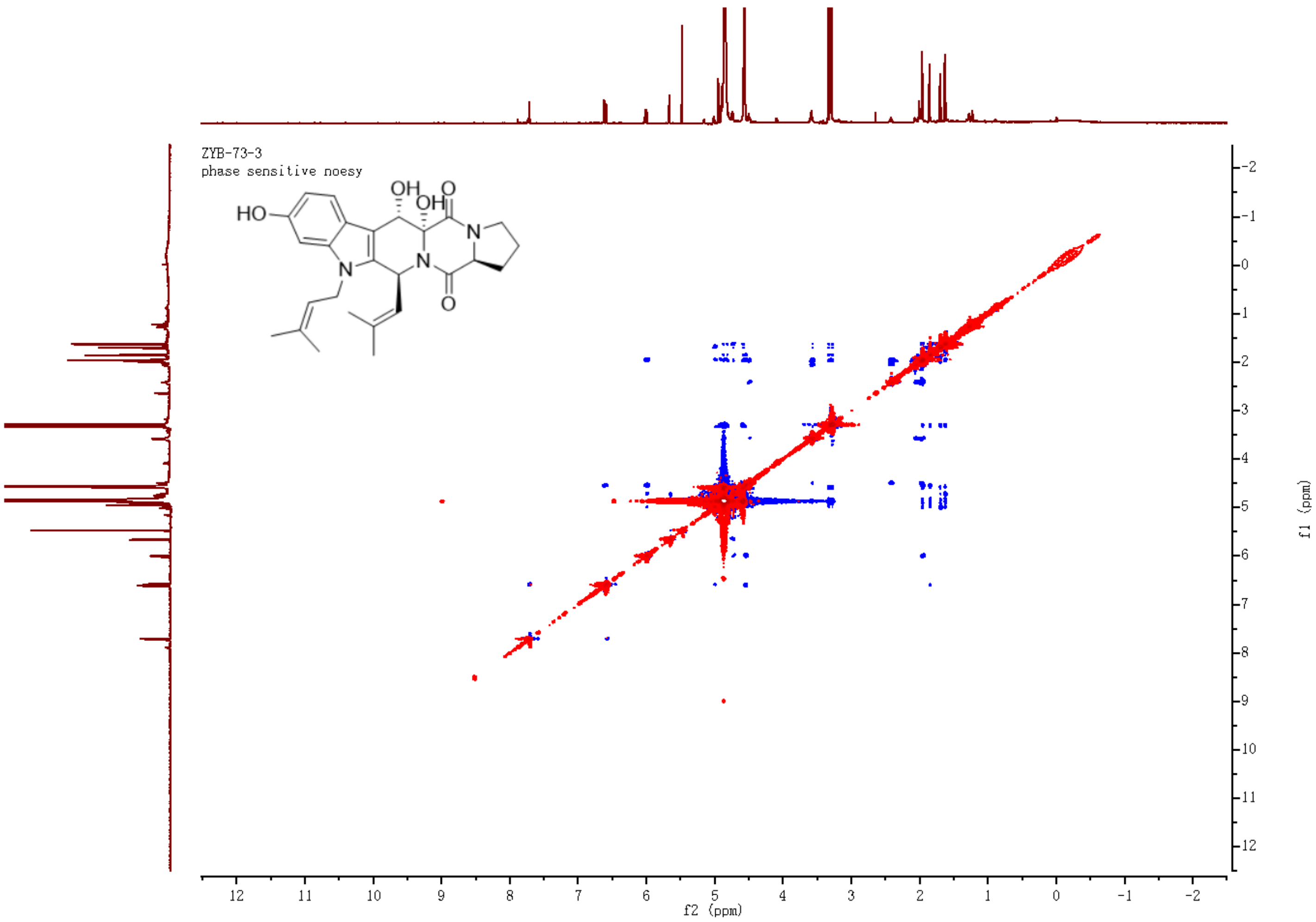

Figure S36. NOESY spectrum of **4** in CD_3OD.

Elemental Composition Report

Single Mass Analysis

Tolerance = 5.0 mDa / DBE: min = -1.5, max = 50.0
Element prediction: Off
Number of isotope peaks used for i-FIT = 3

Monoisotopic Mass, Even Electron Ions
450 formula(e) evaluated with 5 results within limits (up to 50 best isotopic matches for each mass)
Elements Used:
C: 18-30 H: 0-35 N: 0-5 O: 0-20 Na: 0-1
ZYB-73-3-April 47 (0.305) Cm (40:56)
1: TOF MS ES+

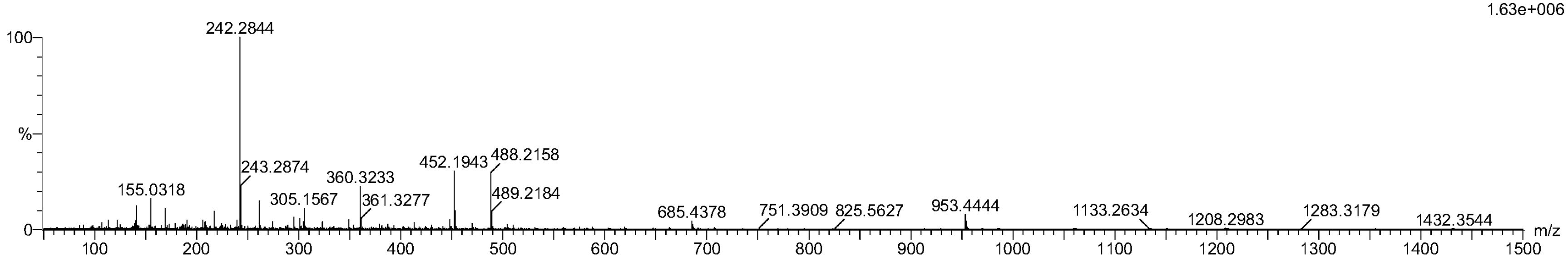

Minimum:				-1.5				
Maximum:		5.0	10.0	50.0				
Mass	Calc. Mass	mDa	PPM	DBE	i-FIT	Norm	Conf(%)	Formula
488.2158	488.2161	-0.3	-0.6	12.5	2106.4	0.458	63.25	C26 H31 N3 O5 Na
	488.2145	1.3	2.7	11.5	2107.6	1.690	18.46	C23 H30 N5 O7
	488.2132	2.6	5.3	6.5	2110.3	4.378	1.25	C22 H34 N O11
	488.2185	-2.7	-5.5	15.5	2107.7	1.828	16.07	C28 H30 N3 O5
	488.2121	3.7	7.6	8.5	2110.5	4.634	0.97	C21 H31 N5 O7 Na

Figure S37. HR-ESI-MS spectrum of **4**.

Anton Paar GmbH
Anton Paar Strasse 10
8054 Graz
Austria

Anton Paar

Anton Paar Polarimeter - Measurement(s)

MCP 100
Software Version: 1.50.4098.87
Serial Number: 99032784

Unique Id 310

- Sample Name: ZYB-73-3
- Date: 02/01/2024 - 08:53 PM
- Username: Administrator
- Sample State: Ok

- Measurement Mode: Specific Rotation
- Measurement Result: 22.000 °
- Concentration: 0.100 g/100ml
- Optical Rotation: 0.022 °
- Set Temperature: 25.0 °C
- Temperature: 25.0 °C

Thursday, February 01, 2024 Page 1 of 1

Figure S3. Specific rotation data of **4**.

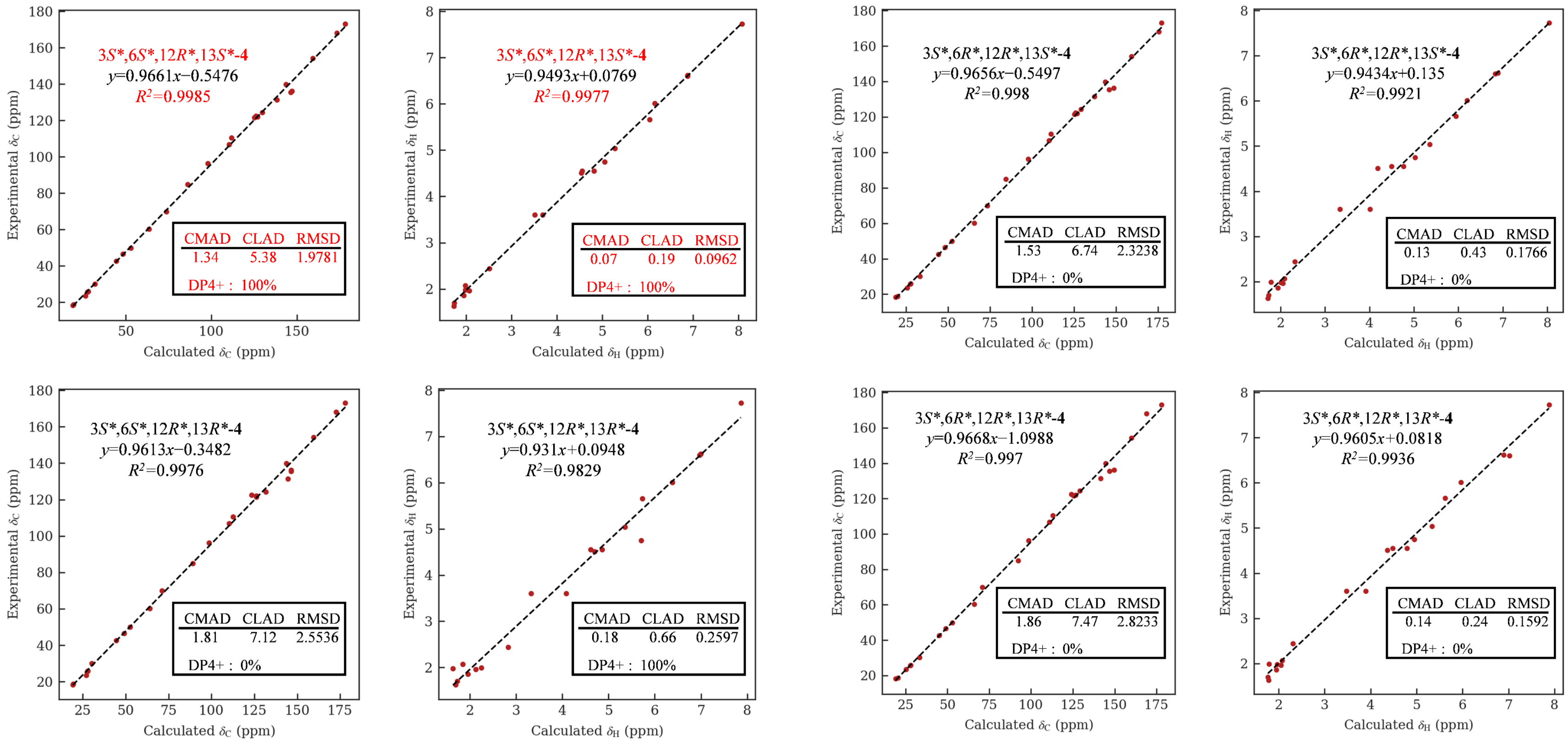

Figure S39. Regression analysis of experimental versus calculated ^{1}H, ^{13}C NMR chemical shifts of **4**.

Functional	Solvent?	Basis Set
mPW1PW91	PCM	6-31G(d)

	Isomer 1	Isomer 2	Isomer 3	Isomer 4
sDP4+ (H data)	100.00%	0.00%	0.00%	0.00%
sDP4+ (C data)	98.09%	0.00%	1.90%	0.00%
sDP4+ (all data)	100.00%	0.00%	0.00%	0.00%
uDP4+ (H data)	29.20%	0.00%	0.03%	70.77%
uDP4+ (C data)	90.89%	2.92%	6.11%	0.07%
uDP4+ (all data)	99.80%	0.00%	0.01%	0.20%
DP4+ (H data)	100.00%	0.00%	0.00%	0.00%
DP4+ (C data)	99.87%	0.00%	0.13%	0.00%
DP4+ (all data)	100.00%	0.00%	0.00%	0.00%

Figure S4. The DP4+ probability analysis of **4**.

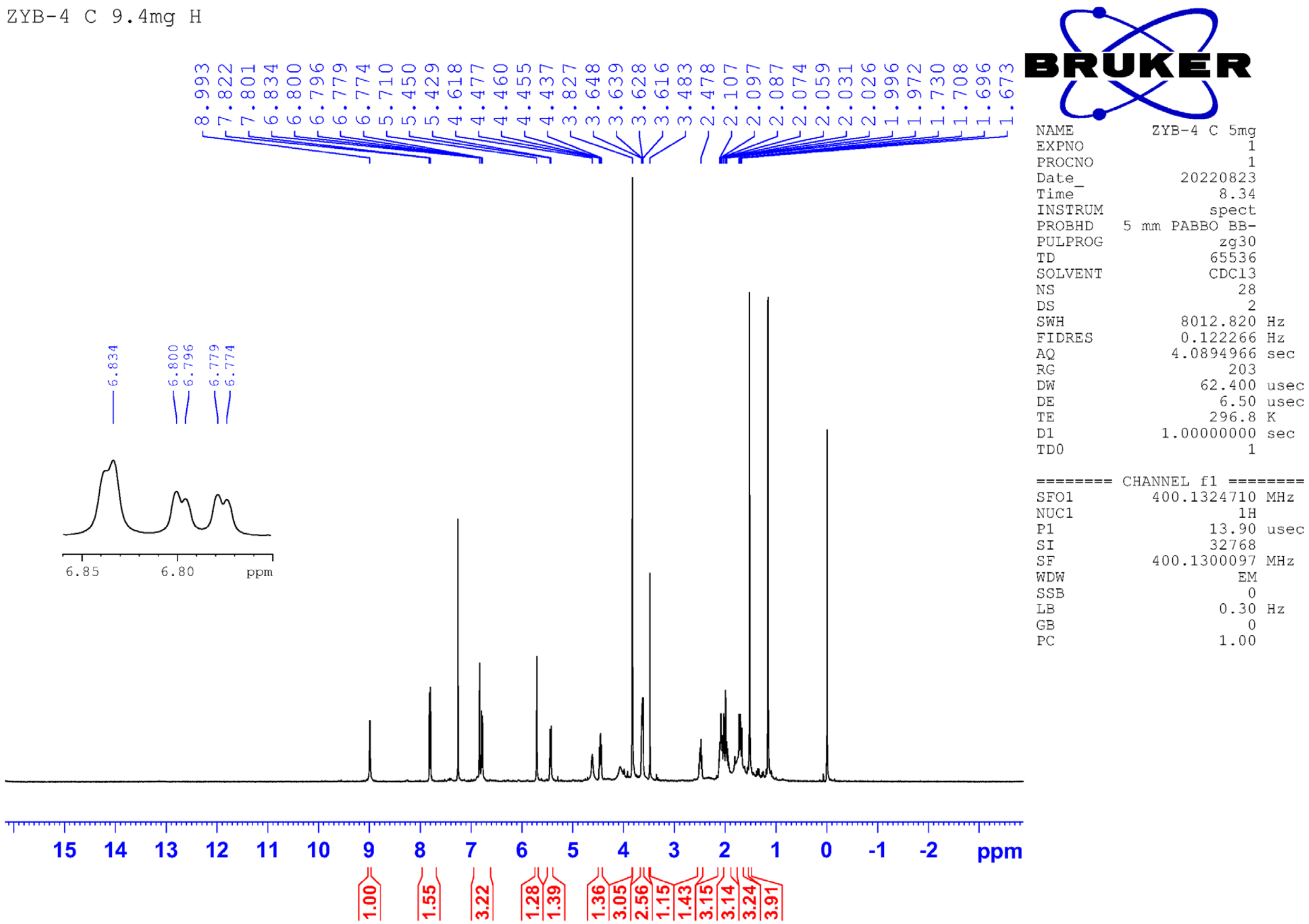

Figure S41. ^{1}H NMR spectrum of **5** in $CDCl_3$.

Elemental Composition Report

Page 1

Single Mass Analysis
Tolerance = 5.0 mDa / DBE: min = -1.5, max = 50.0
Element prediction: Off
Number of isotope peaks used for i-FIT = 3

Monoisotopic Mass, Even Electron Ions
1 formula(e) evaluated with 0 results within limits (up to 50 best isotopic matches for each mass)
Elements Used:
C: 0-35 H: 0-50 N: 0-10 O: 0-15 23Na: 0-1
ZYB-4-N 70 (0.290) Cm (57:102)
1: TOF MS ES-

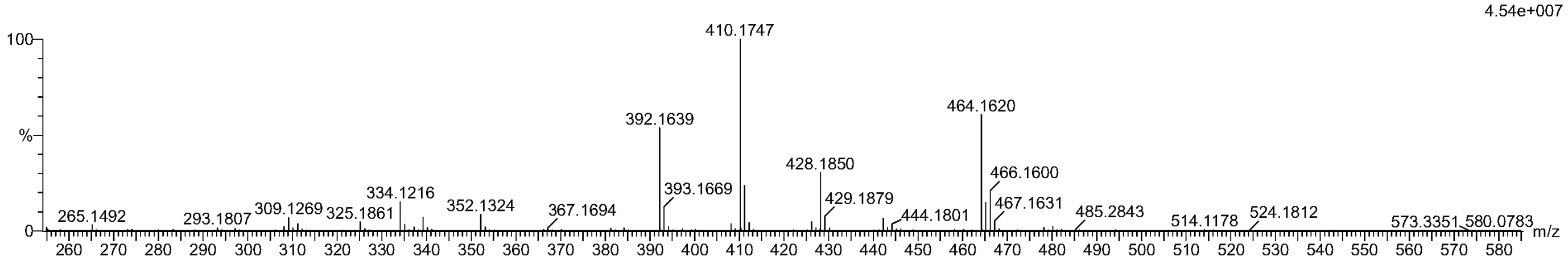


```
Minimum:                          -1.5
Maximum:              5.0   10.0  50.0

Mass     Calc. Mass  mDa  PPM   DBE   i-FIT   Norm   Conf(%)  Formula
0.0000   ---
```

Figure S42. HR-ESI-MS spectrum of **5**.

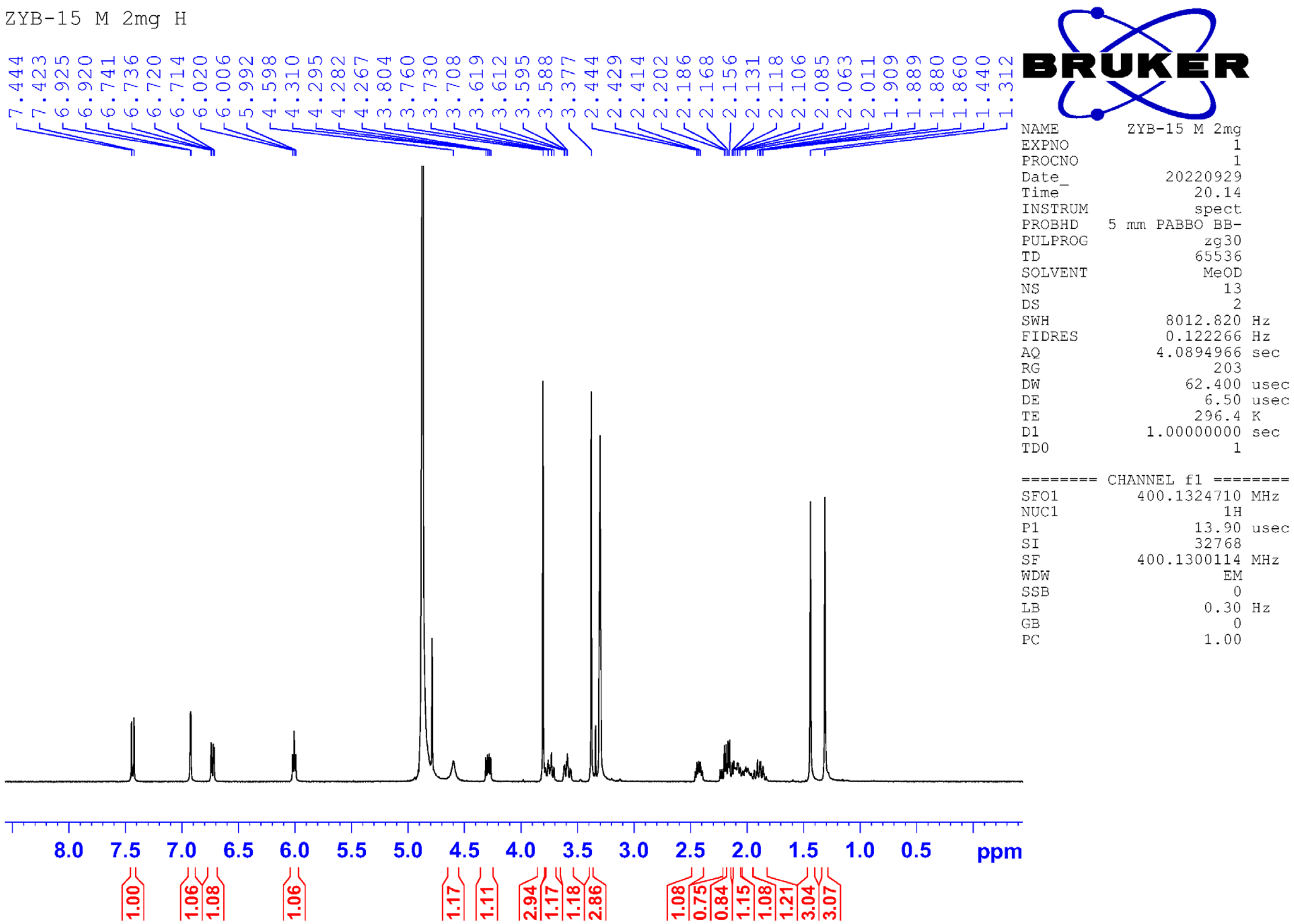

Figure S43. ^{1}H NMR spectrum of **6** in CD_3OD.

Tolerance = 5.0 mDa / DBE: min = -1.5, max = 50.0
Element prediction: Off
Number of isotope peaks used for i-FIT = 3

Monoisotopic Mass, Even Electron Ions
1031 formula(e) evaluated with 11 results within limits (up to 50 best isotopic matches for each mass)
Elements Used:
C: 0-50 H: 0-50 N: 0-10 O: 0-10 23Na: 0-1
ZYB-15 72 (0.297) Cm (67:85)
1: TOF MS ES+

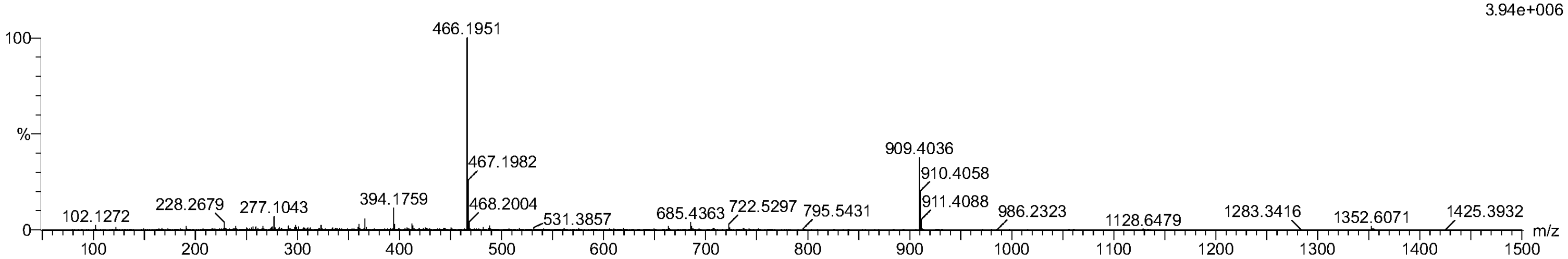

Minimum:	80.00				-1.5				
Maximum:	100.00		5.0	10.0	50.0				

Mass	RA	Calc. Mass	mDa	PPM	DBE	i-FIT	Norm	Conf(%)	Formula
466.1951	100.00	466.1951	0.0	0.0	14.5	1617.8	0.068	93.47	C21 H24 N9 O4
		466.1954	-0.3	-0.6	10.5	1621.0	3.236	3.93	C23 H29 N3 O6 23Na
		466.1938	1.3	2.8	9.5	1621.8	4.087	1.68	C20 H28 N5 O8
		466.1927	2.4	5.1	11.5	1622.7	4.991	0.68	C19 H25 N9 O4 23Na
		466.1967	-1.6	-3.4	15.5	1624.0	6.257	0.19	C24 H25 N7 O2 23Na
		466.1978	-2.7	-5.8	13.5	1625.9	8.224	0.03	C25 H28 N3 O6
		466.1914	3.7	7.9	6.5	1626.4	8.722	0.02	C18 H29 N5 O8 23Na
		466.1991	-4.0	-8.6	18.5	1628.2	10.474	0.00	C26 H24 N7 O2
		466.1994	-4.3	-9.2	14.5	1629.5	11.819	0.00	C28 H29 N O4 23Na
		466.1986	-3.5	-7.5	2.5	1630.5	12.753	0.00	C12 H29 N9 O9 23Na
		466.1919	3.2	6.9	22.5	1630.8	13.109	0.00	C32 H24 N3 O

Figure S44. HR-ESI-MS spectrum of **6**.

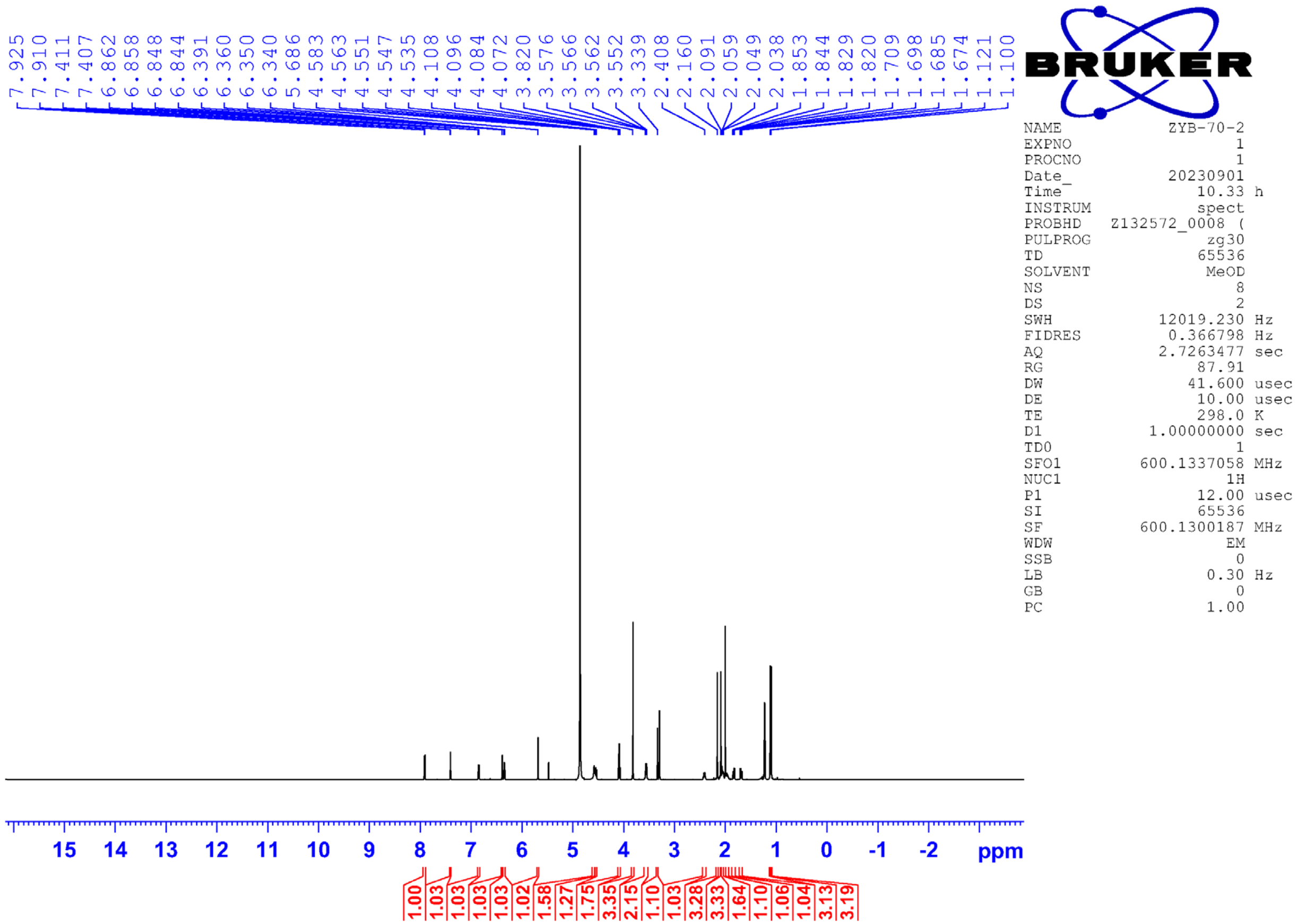

Figure S45. ^{1}H NMR spectrum of **7** in CD_3OD.

Single Mass Analysis
Tolerance = 5.0 mDa / DBE: min = -1.5, max = 50.0
Element prediction: Off
Number of isotope peaks used for i-FIT = 3

Monoisotopic Mass, Even Electron Ions
1 formula(e) evaluated with 0 results within limits (up to 50 best isotopic matches for each mass)
Elements Used:
C: 0-30 H: 0-60 O: 0-10 23Na: 0-1
ZYB-70-2-April 40 (0.258) Cm (38:46)
1: TOF MS ES+

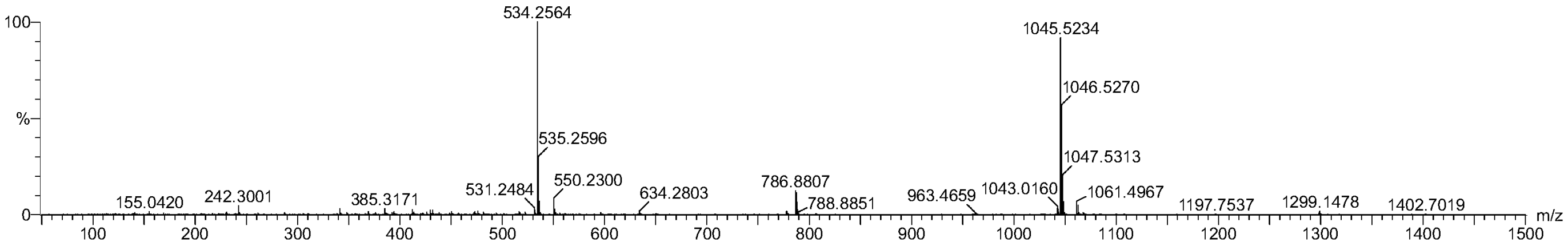

Minimum:				-1.5				
Maximum:		5.0	10.0	50.0				
Mass	Calc. Mass	mDa	PPM	DBE	i-FIT	Norm	Conf(%)	Formula
0.0000	---							

Figure S46. HR-ESI-MS spectrum of **7**.

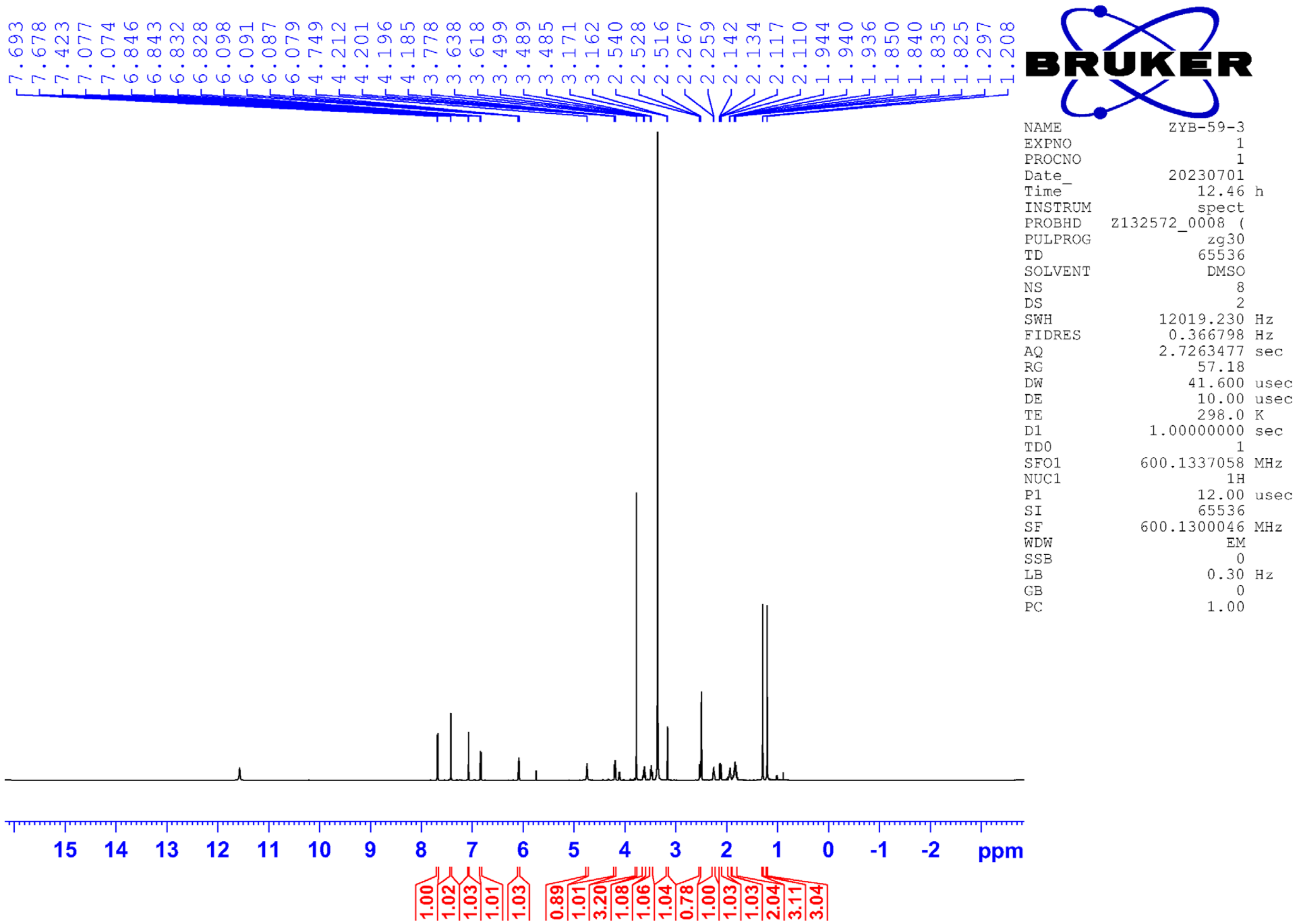

Figure S47. ^{1}H NMR spectrum of **8** in DMSO-d_6.

Elemental Composition Report **Page 1**

Single Mass Analysis
Tolerance = 5.0 mDa / DBE: min = -1.5, max = 50.0
Element prediction: Off
Number of isotope peaks used for i-FIT = 3

Monoisotopic Mass, Even Electron Ions
1 formula(e) evaluated with 0 results within limits (up to 50 best isotopic matches for each mass)
Elements Used:
C: 0-30 H: 0-60 O: 0-10 23Na: 0-1
ZYB-59-3-April 50 (0.316) Cm (41:50)
1: TOF MS ES+

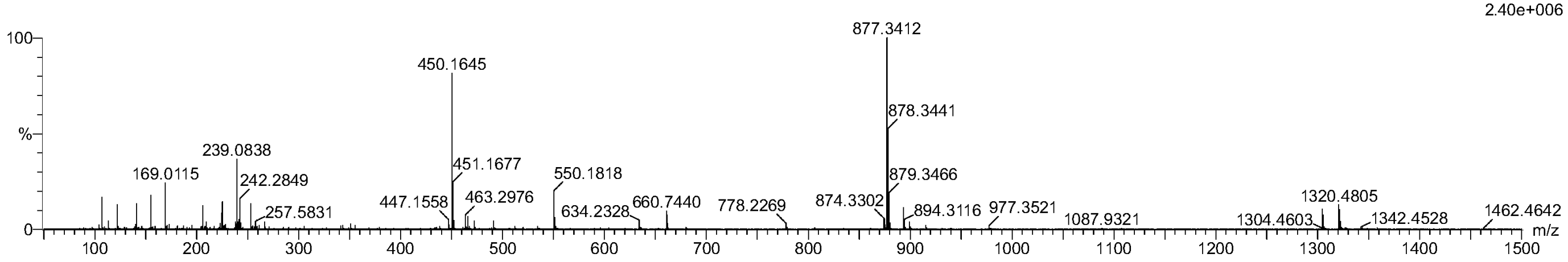

Minimum:				-1.5				
Maximum:		5.0	10.0	50.0				
Mass	Calc. Mass	mDa	PPM	DBE	i-FIT	Norm	Conf(%)	Formula
0.0000	---							

Figure S48. HR-ESI-MS spectrum of **8**.

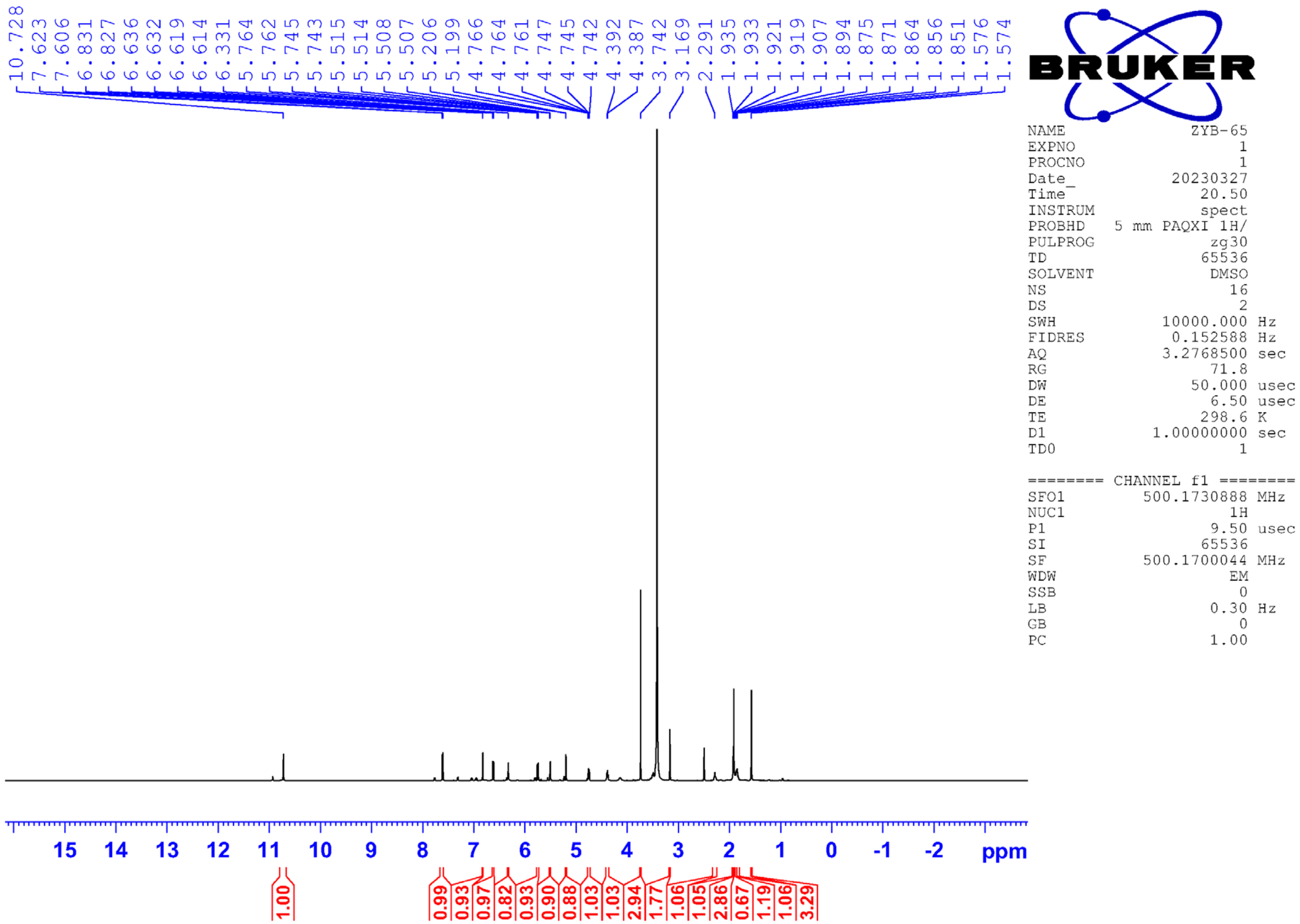

Figure S49. ^{1}H NMR spectrum of **9** in DMSO-d_6.

Elemental Composition Report

Single Mass Analysis

Tolerance = 5.0 mDa / DBE: min = -1.5, max = 50.0
Element prediction: Off
Number of isotope peaks used for i-FIT = 3

Monoisotopic Mass, Even Electron Ions
1 formula(e) evaluated with 0 results within limits (up to 50 best isotopic matches for each mass)
Elements Used:
C: 0-30 H: 0-60 O: 0-10 23Na: 0-1
ZYB-65-April 123 (0.778) Cm (120:126)
1: TOF MS ES+

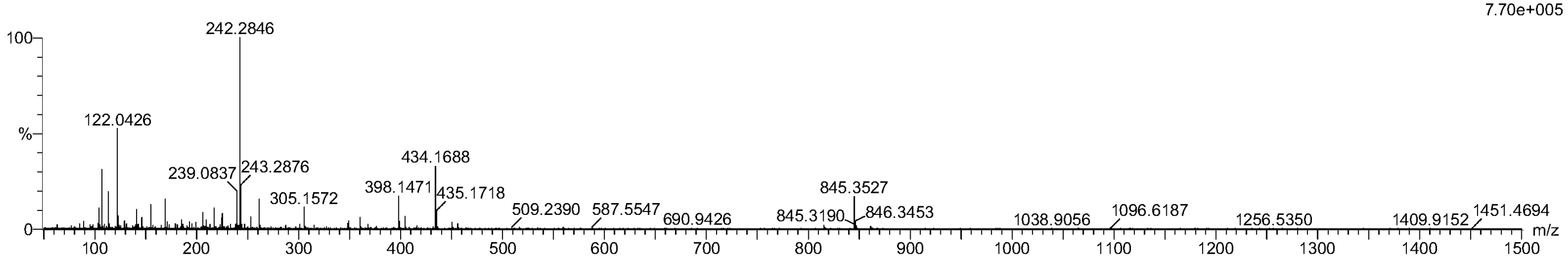

Minimum:				-1.5
Maximum:		5.0	10.0	50.0

Mass	Calc. Mass	mDa	PPM	DBE	i-FIT	Norm	Conf(%)	Formula
0.0000	---							

Figure S50. HR-ESI-MS spectrum of **9**.

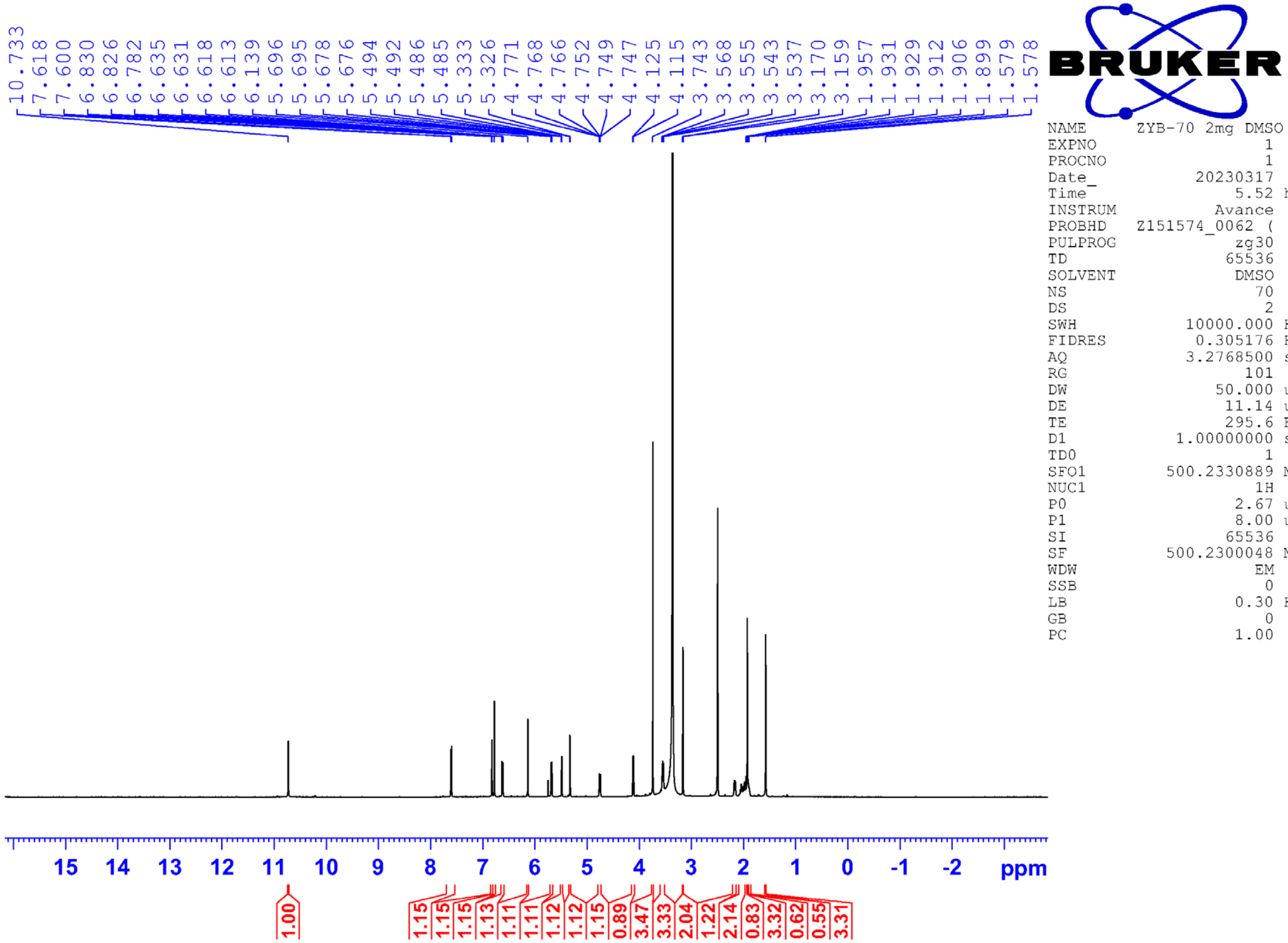

Figure S51. ^{1}H NMR spectrum of **10** in DMSO-d_6.

Elemental Composition Report **Page 1**

Single Mass Analysis
Tolerance = 5.0 mDa / DBE: min = -1.5, max = 50.0
Element prediction: Off
Number of isotope peaks used for i-FIT = 3

Monoisotopic Mass, Even Electron Ions
1 formula(e) evaluated with 0 results within limits (up to 50 best isotopic matches for each mass)
Elements Used:
C: 0-30 H: 0-60 O: 0-10 23Na: 0-1
ZYB-70-April 36 (0.245) Cm (32:44)
1: TOF MS ES+

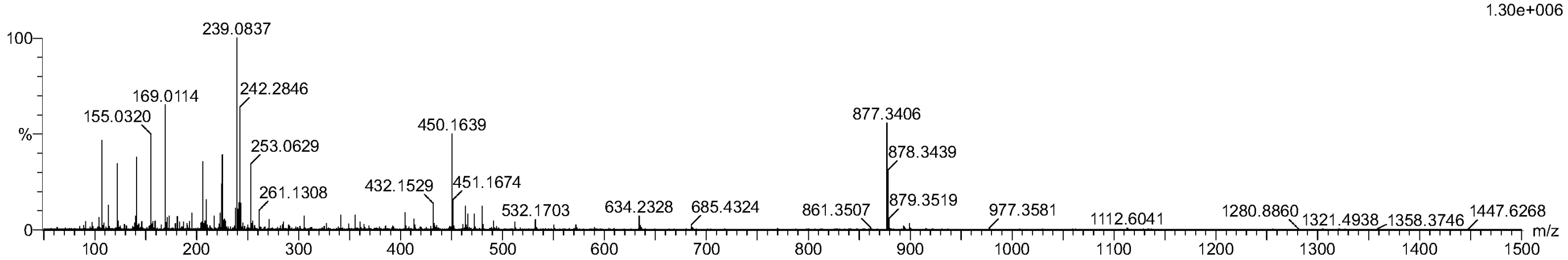

Minimum:				-1.5				
Maximum:		5.0	10.0	50.0				
Mass	Calc. Mass	mDa	PPM	DBE	i-FIT	Norm	Conf(%)	Formula
0.0000	---							

Figure S52. HR-ESI-MS spectrum of **10**.

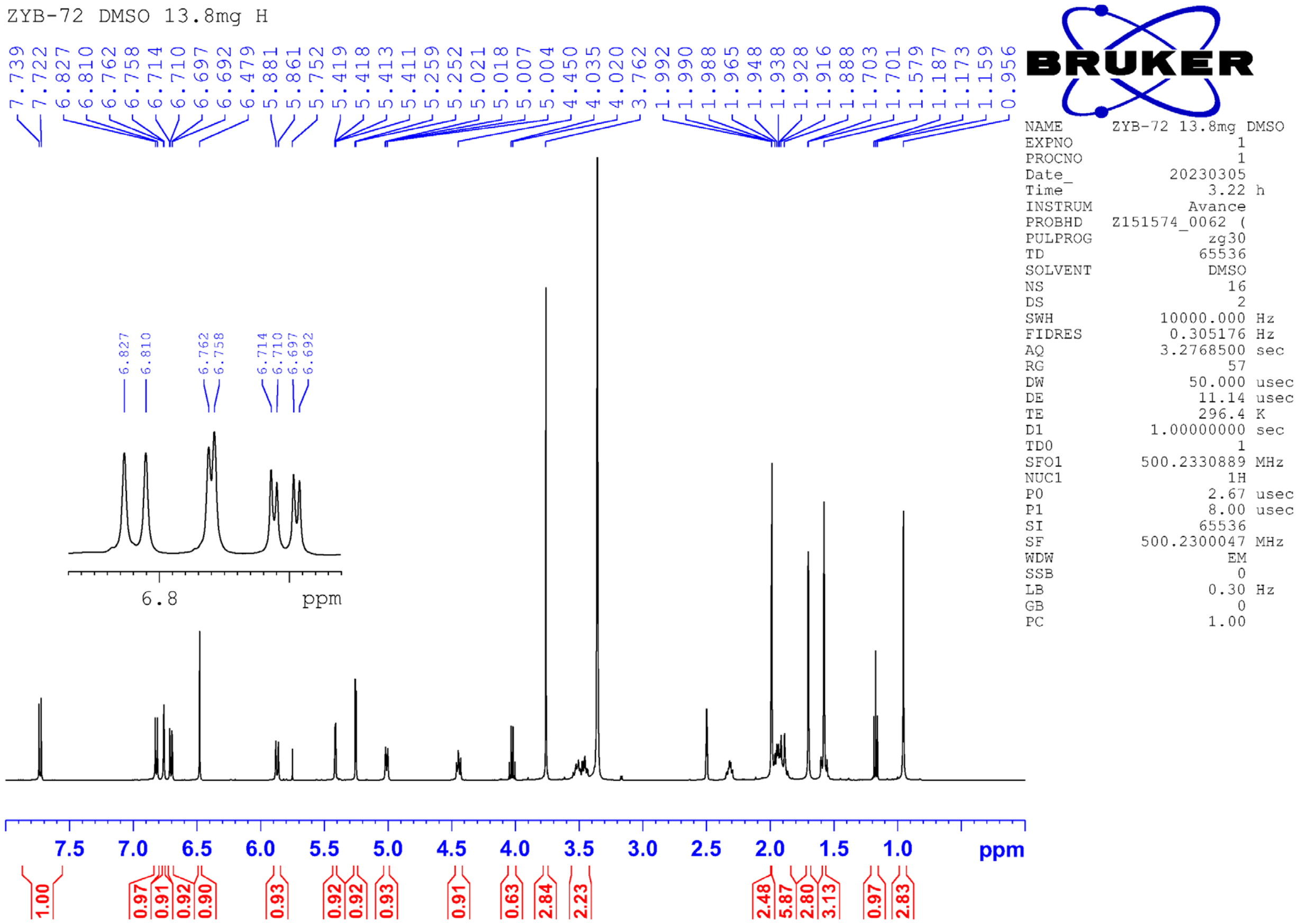

Figure S53. ^{1}H NMR spectrum of **11** in DMSO-d_6.

Single Mass Analysis

Tolerance = 5.0 mDa / DBE: min = -1.5, max = 50.0
Element prediction: Off
Number of isotope peaks used for i-FIT = 3

Monoisotopic Mass, Even Electron Ions
1 formula(e) evaluated with 0 results within limits (up to 50 best isotopic matches for each mass)
Elements Used:
C: 0-30 H: 0-60 O: 0-10 23Na: 0-1
ZYB-72-April 95 (0.615) Cm (90:115)
1: TOF MS ES+

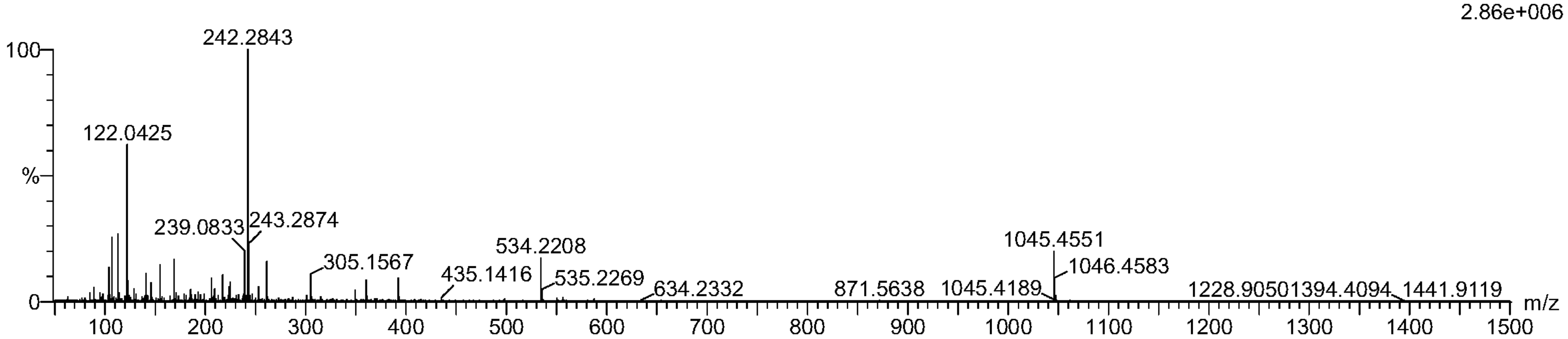

Minimum:				-1.5				
Maximum:		5.0	10.0	50.0				
Mass	Calc. Mass	mDa	PPM	DBE	i-FIT	Norm	Conf(%)	Formula
0.0000	---							

Figure S54. HR-ESI-MS spectrum of **11**.

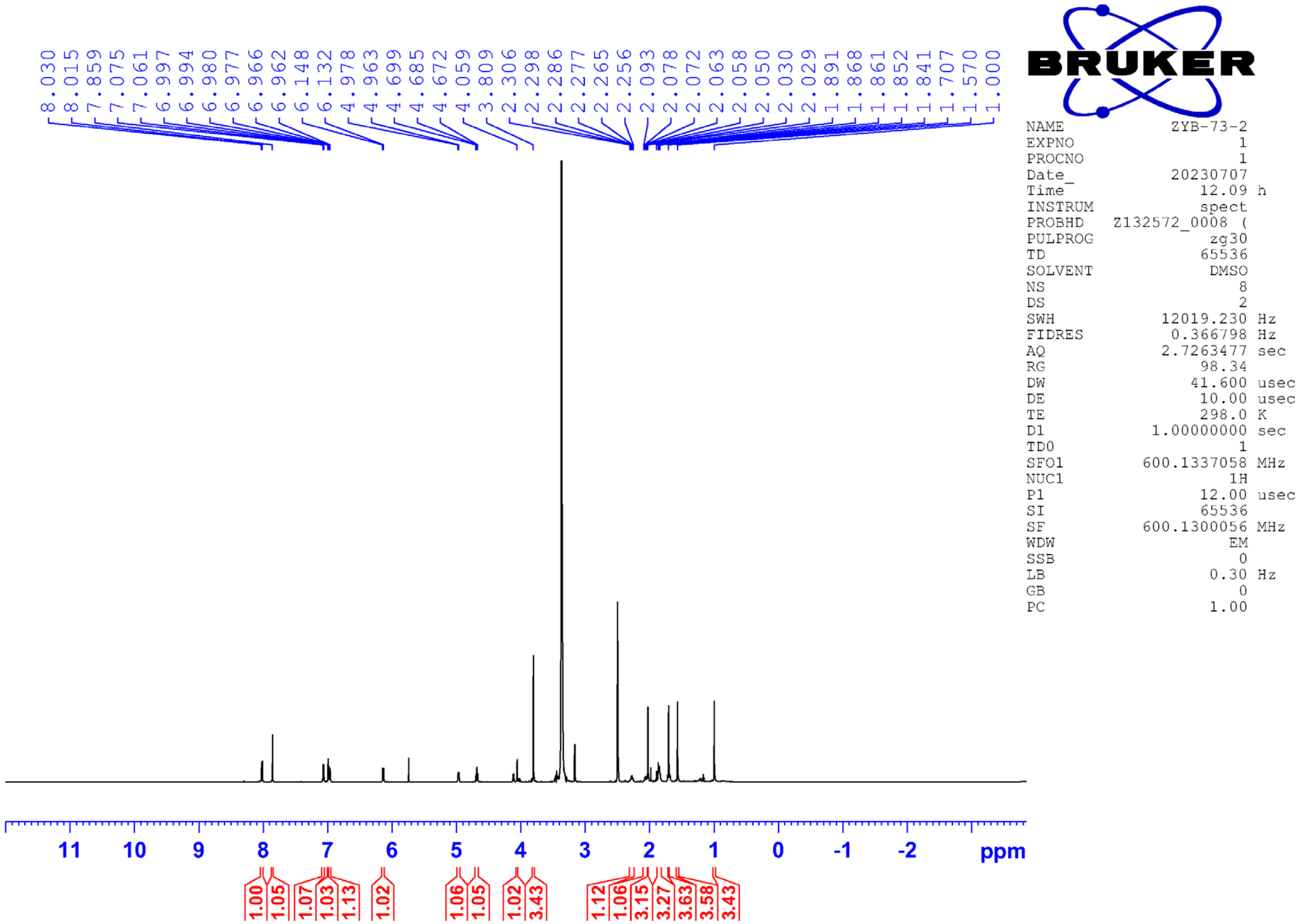

Figure S55. ^{1}H NMR spectrum of **12** in DMSO-d_6.

Elemental Composition Report **Page 1**

Single Mass Analysis
Tolerance = 5.0 mDa / DBE: min = -1.5, max = 50.0
Element prediction: Off
Number of isotope peaks used for i-FIT = 3

Monoisotopic Mass, Even Electron Ions
1 formula(e) evaluated with 0 results within limits (up to 50 best isotopic matches for each mass)
Elements Used:
C: 0-30 H: 0-60 O: 0-10 23Na: 0-1
ZYB-73-2-April 44 (0.285) Cm (40:48)
1: TOF MS ES+

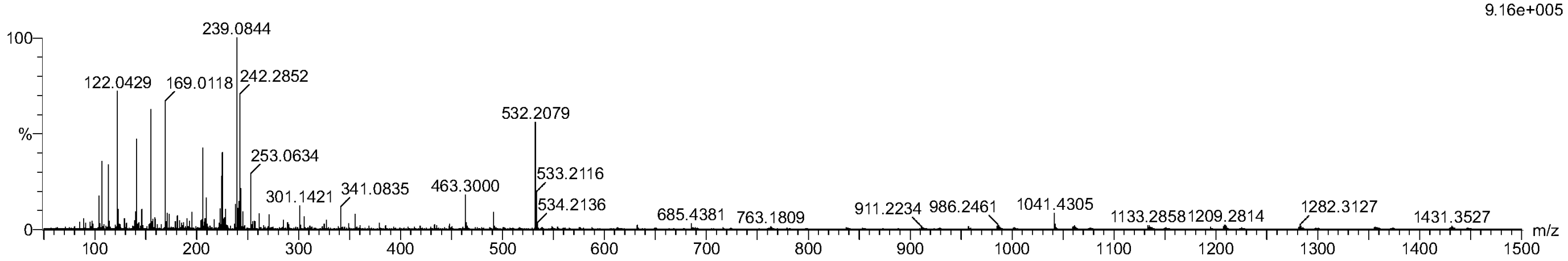

Minimum:				-1.5				
Maximum:		5.0	10.0	50.0				
Mass	Calc. Mass	mDa	PPM	DBE	i-FIT	Norm	Conf(%)	Formula
0.0000	---							

Figure S56. HR-ESI-MS spectrum of **12**.

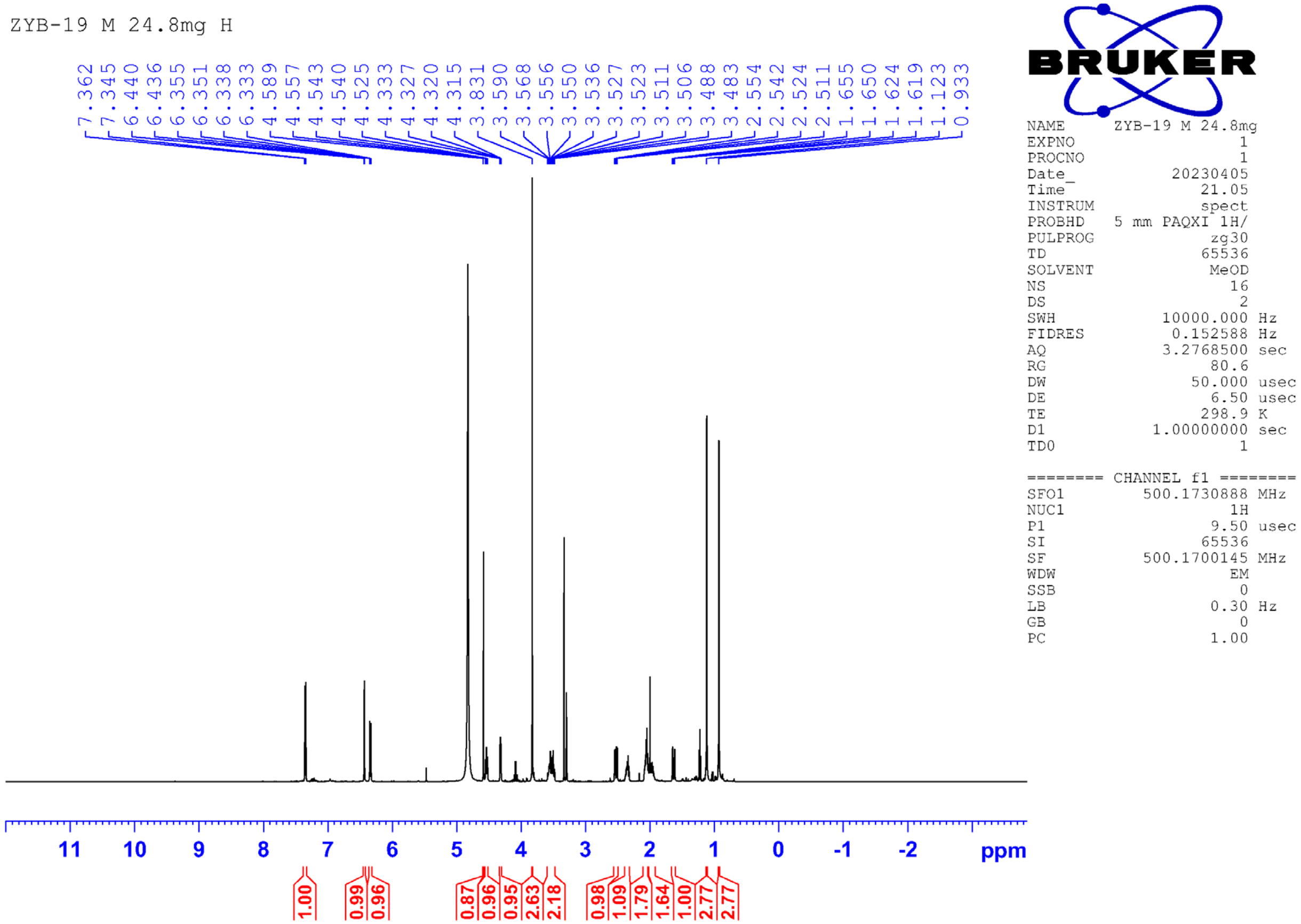

Figure S57. ^{1}H NMR spectrum of **13** in CD_3OD.

Elemental Composition Report **Page 1**

Single Mass Analysis
Tolerance = 5.0 mDa / DBE: min = -1.5, max = 50.0
Element prediction: Off
Number of isotope peaks used for i-FIT = 3

Monoisotopic Mass, Even Electron Ions
1 formula(e) evaluated with 0 results within limits (up to 50 best isotopic matches for each mass)
Elements Used:
C: 0-30 H: 0-60 O: 0-10 23Na: 0-1
ZYB-19 74 (0.304) Cm (69:77)
1: TOF MS ES+

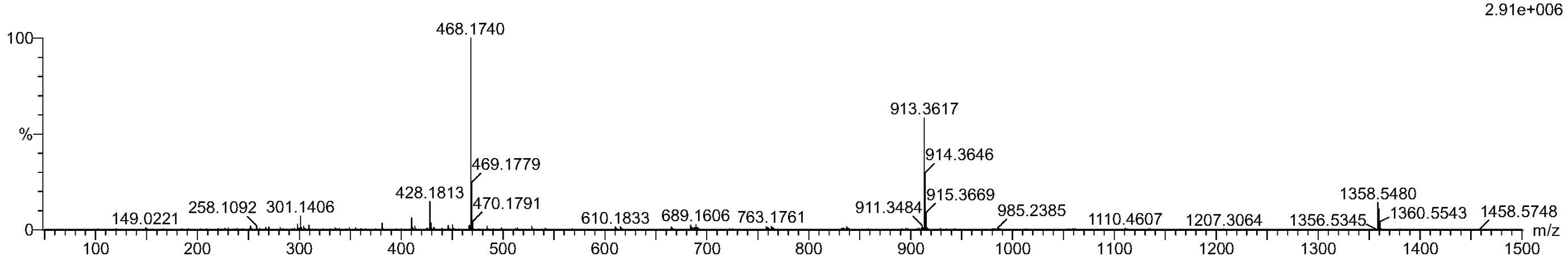

Minimum:				-1.5				
Maximum:		5.0	10.0	50.0				
Mass	Calc. Mass	mDa	PPM	DBE	i-FIT	Norm	Conf(%)	Formula
0.0000	---							

Figure S58. HR-ESI-MS spectrum of **13**.

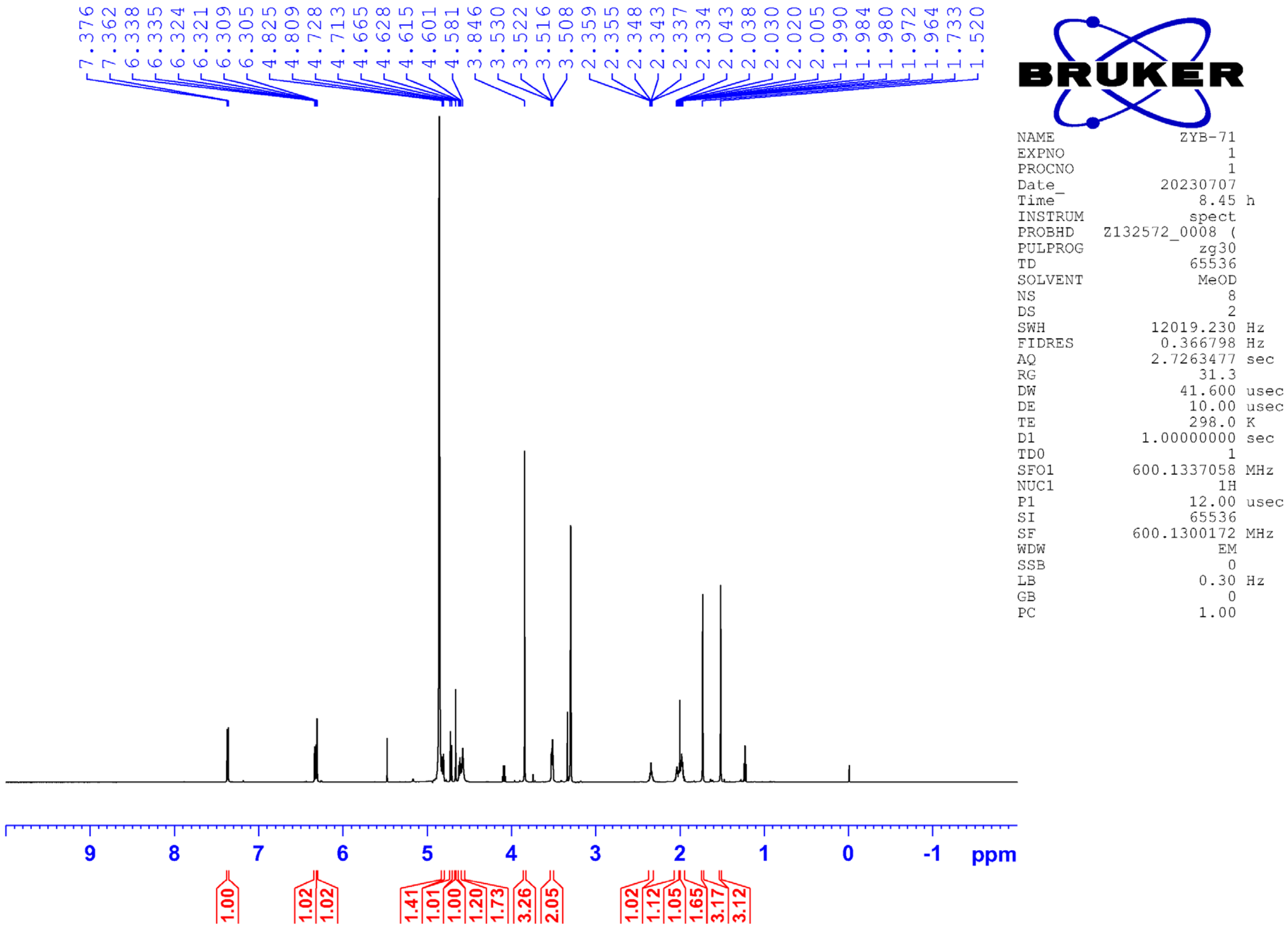

Figure S59. ^{1}H NMR spectrum of **14** in CD_3OD.

Elemental Composition Report

Single Mass Analysis

Tolerance = 5.0 mDa / DBE: min = -1.5, max = 50.0
Element prediction: Off
Number of isotope peaks used for i-FIT = 3

Monoisotopic Mass, Even Electron Ions
1 formula(e) evaluated with 0 results within limits (up to 50 best isotopic matches for each mass)
Elements Used:
C: 0-30 H: 0-60 O: 0-10 23Na: 0-1

ZYB-71-April 35 (0.268) Cm (33:39)
1: TOF MS ES+

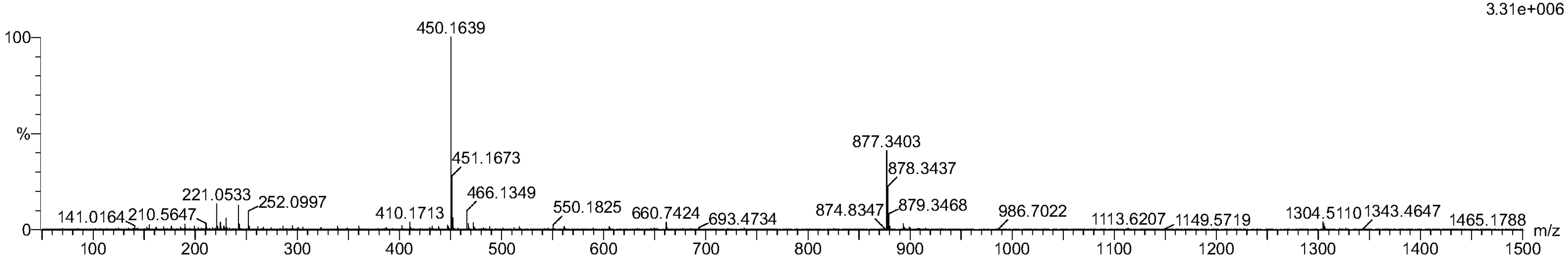

Minimum:				-1.5				
Maximum:		5.0	10.0	50.0				
Mass	Calc. Mass	mDa	PPM	DBE	i-FIT	Norm	Conf(%)	Formula
0.0000	---							

Figure S60. HR-ESI-MS spectrum of **14**.

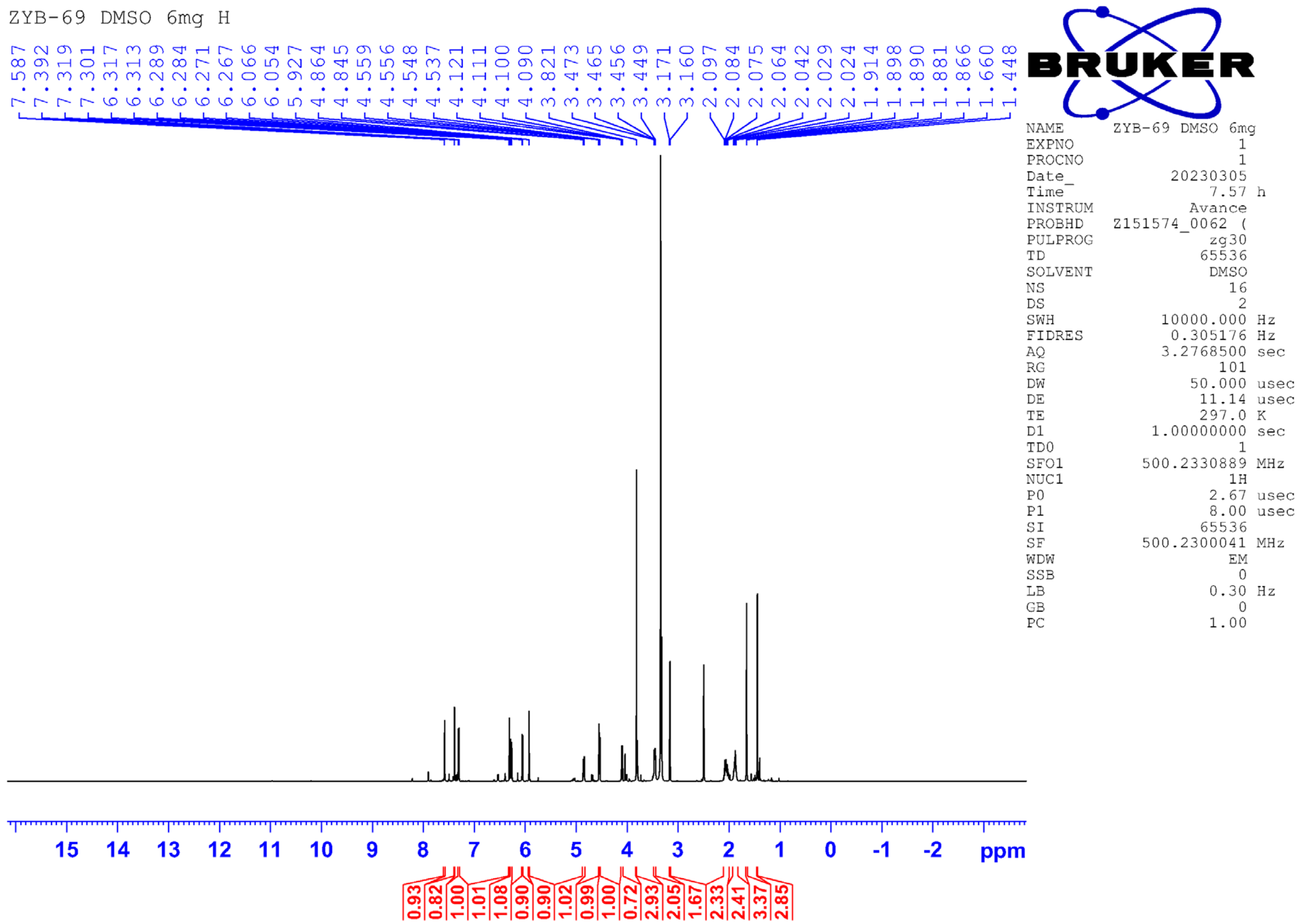

Figure S61. ^{1}H NMR spectrum of **15** in DMSO-d_6.

Elemental Composition Report **Page 1**

Single Mass Analysis
Tolerance = 5.0 mDa / DBE: min = -1.5, max = 50.0
Element prediction: Off
Number of isotope peaks used for i-FIT = 3

Monoisotopic Mass, Even Electron Ions
1 formula(e) evaluated with 0 results within limits (up to 50 best isotopic matches for each mass)
Elements Used:
C: 0-30 H: 0-60 O: 0-10 23Na: 0-1
ZYB-69-April 35 (0.251) Cm (32:38)
1: TOF MS ES+

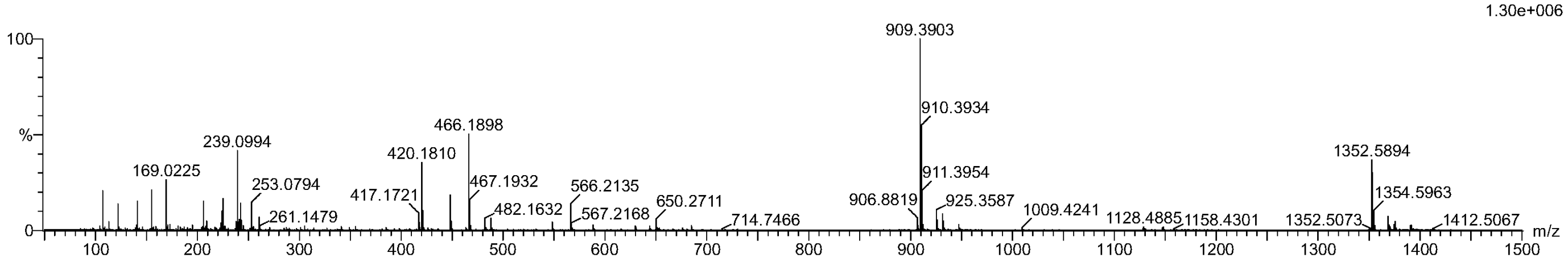

Minimum:				-1.5				
Maximum:		5.0	10.0	50.0				
Mass	Calc. Mass	mDa	PPM	DBE	i-FIT	Norm	Conf(%)	Formula
0.0000	---							

Figure S62. HR-ESI-MS spectrum of **15**.

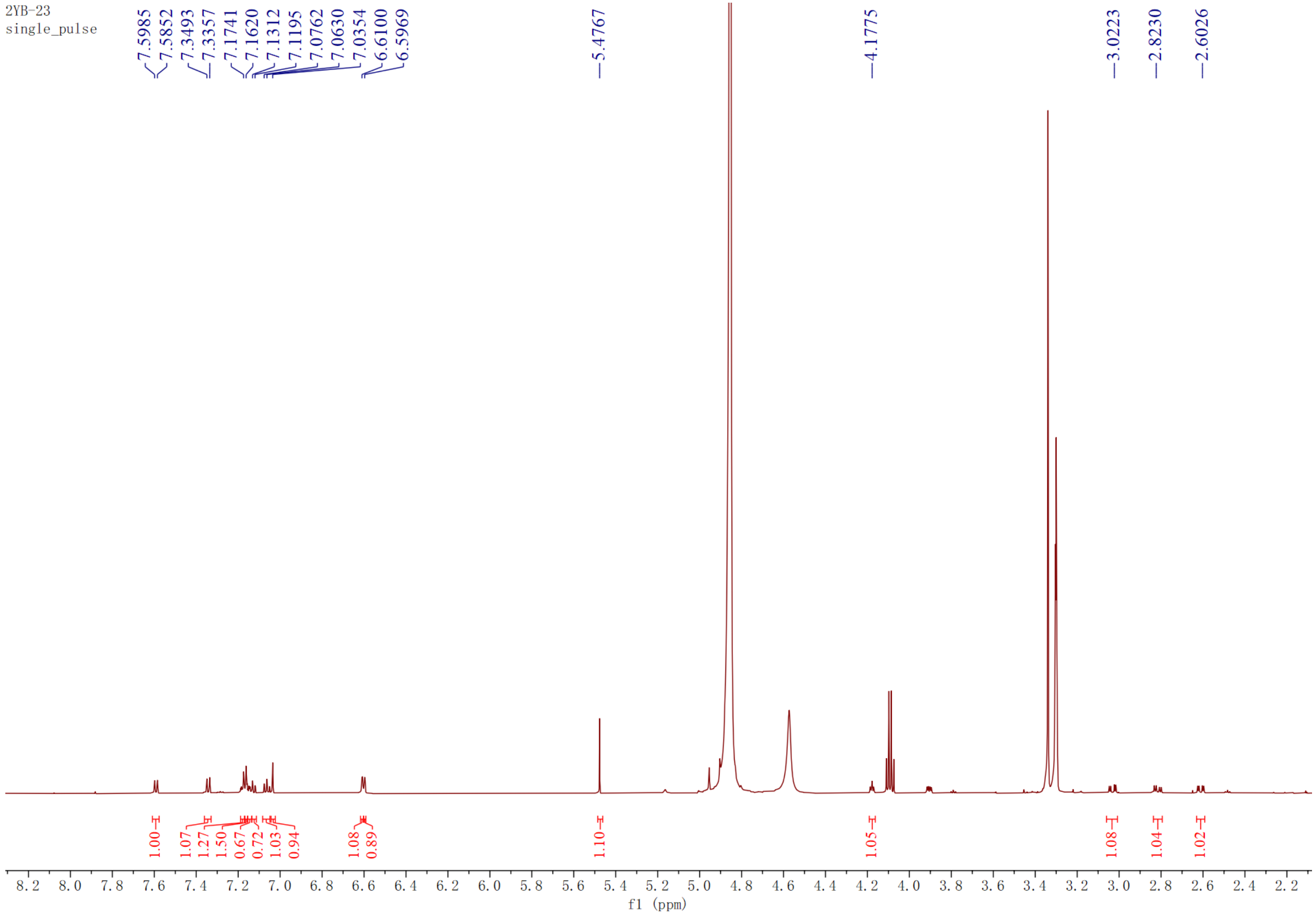

Figure S63. ^{1}H NMR spectrum of **16** in CD_3OD.

Elemental Composition Report **Page 1**

Single Mass Analysis
Tolerance = 5.0 mDa / DBE: min = -1.5, max = 50.0
Element prediction: Off
Number of isotope peaks used for i-FIT = 3

Monoisotopic Mass, Even Electron Ions
1 formula(e) evaluated with 0 results within limits (up to 50 best isotopic matches for each mass)
Elements Used:
C: 0-30 H: 0-60 O: 0-10 23Na: 0-1
ZYB-23 70 (0.290) Cm (70:73)
1: TOF MS ES+

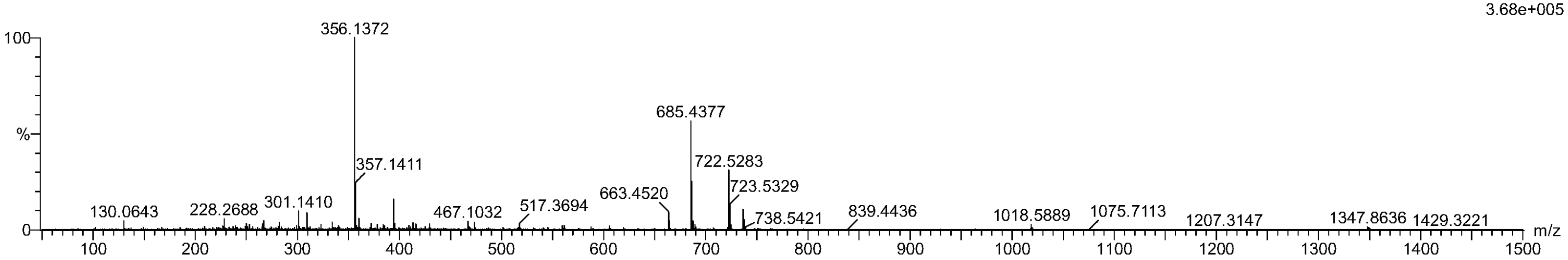

Minimum:				-1.5				
Maximum:		5.0	10.0	50.0				
Mass	Calc. Mass	mDa	PPM	DBE	i-FIT	Norm	Conf(%)	Formula
0.0000	---							

Figure S64. HR-ESI-MS spectrum of **16**.

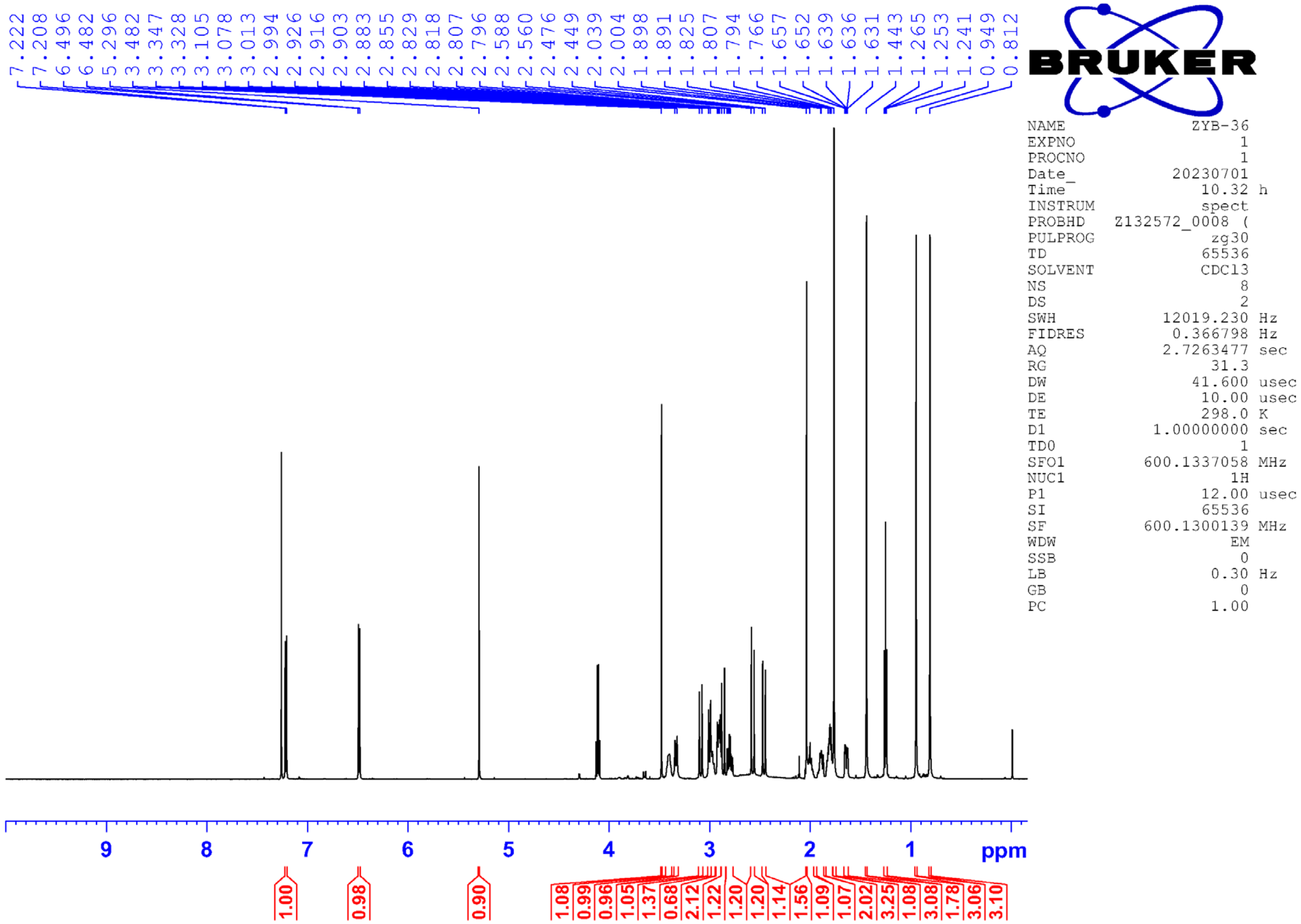

Figure S65. ^{1}H NMR spectrum of **17** in $CDCl_3$.

Single Mass Analysis
Tolerance = 5.0 mDa / DBE: min = -1.5, max = 50.0
Element prediction: Off
Number of isotope peaks used for i-FIT = 3

Monoisotopic Mass, Even Electron Ions
1 formula(e) evaluated with 0 results within limits (up to 50 best isotopic matches for each mass)
Elements Used:
C: 0-30 H: 0-60 O: 0-10 23Na: 0-1
ZYB-36 75 (0.308) Cm (70:77)
1: TOF MS ES+

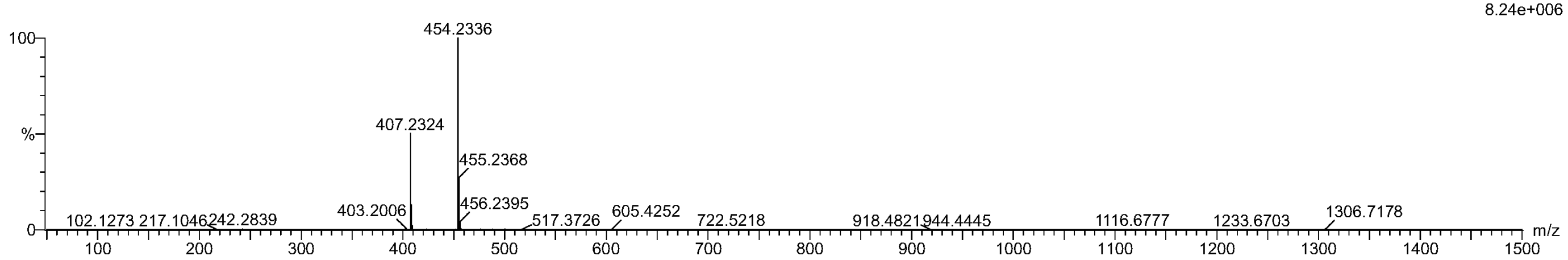

Minimum:				-1.5				
Maximum:		5.0	10.0	50.0				
Mass	Calc. Mass	mDa	PPM	DBE	i-FIT	Norm	Conf(%)	Formula
0.0000	---							

Figure S66. HR-ESI-MS spectrum of **17**.

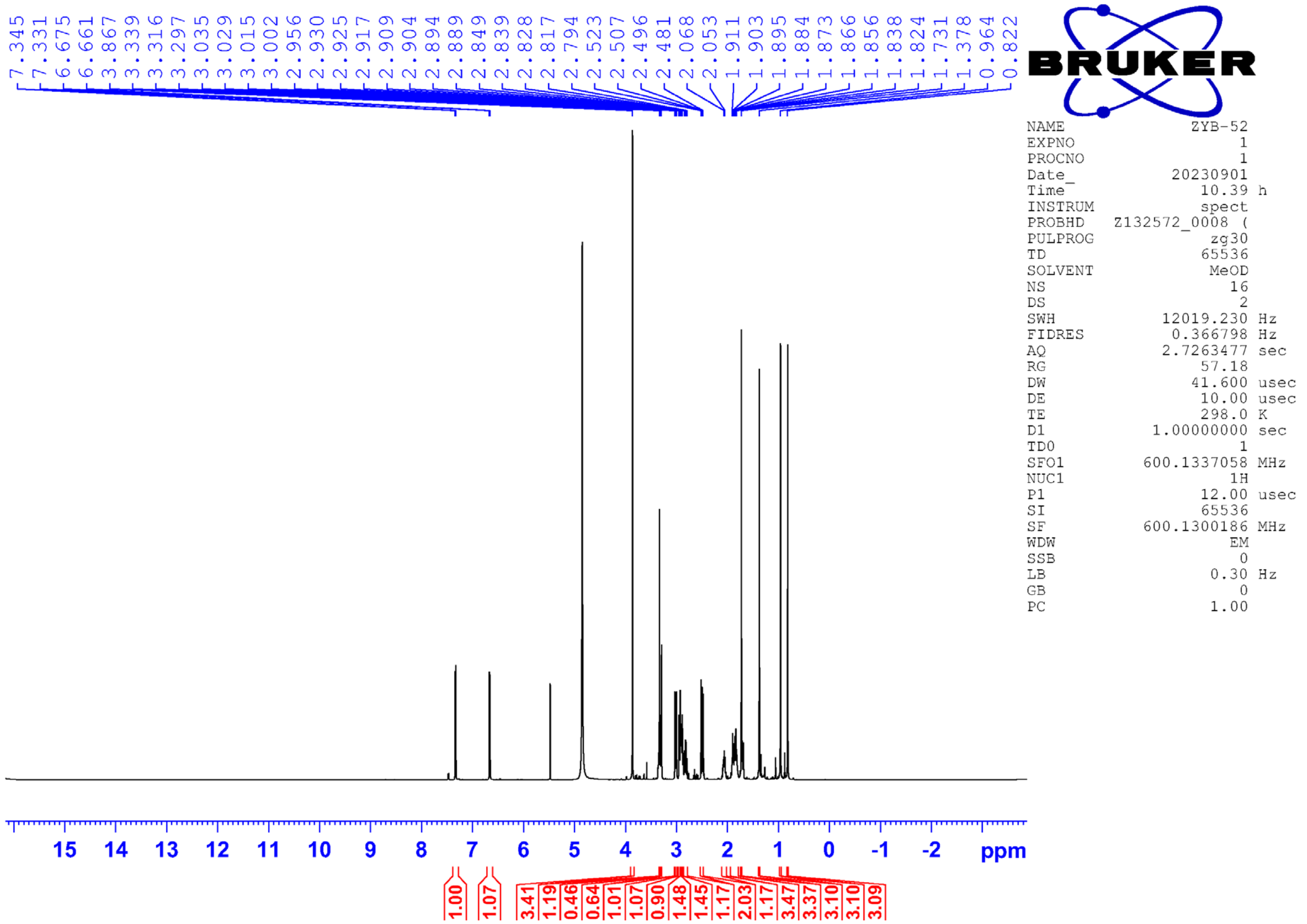

Figure S67. ^{1}H NMR spectrum of **18** in CD_3OD.

Elemental Composition Report **Page 1**

Single Mass Analysis
Tolerance = 5.0 mDa / DBE: min = -1.5, max = 50.0
Element prediction: Off
Number of isotope peaks used for i-FIT = 3

Monoisotopic Mass, Even Electron Ions
1 formula(e) evaluated with 0 results within limits (up to 50 best isotopic matches for each mass)
Elements Used:
C: 0-30 H: 0-60 O: 0-10 23Na: 0-1
ZYB-52-April 32 (0.226) Cm (31:42)
1: TOF MS ES+

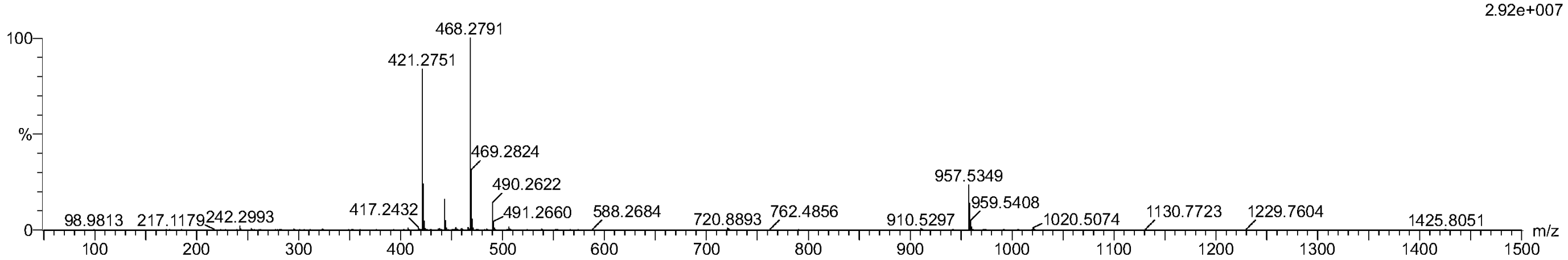

Minimum:				-1.5				
Maximum:		5.0	10.0	50.0				

Mass	Calc. Mass	mDa	PPM	DBE	i-FIT	Norm	Conf(%)	Formula
0.0000	---							

Figure S68. HR-ESI-MS spectrum of **18**.

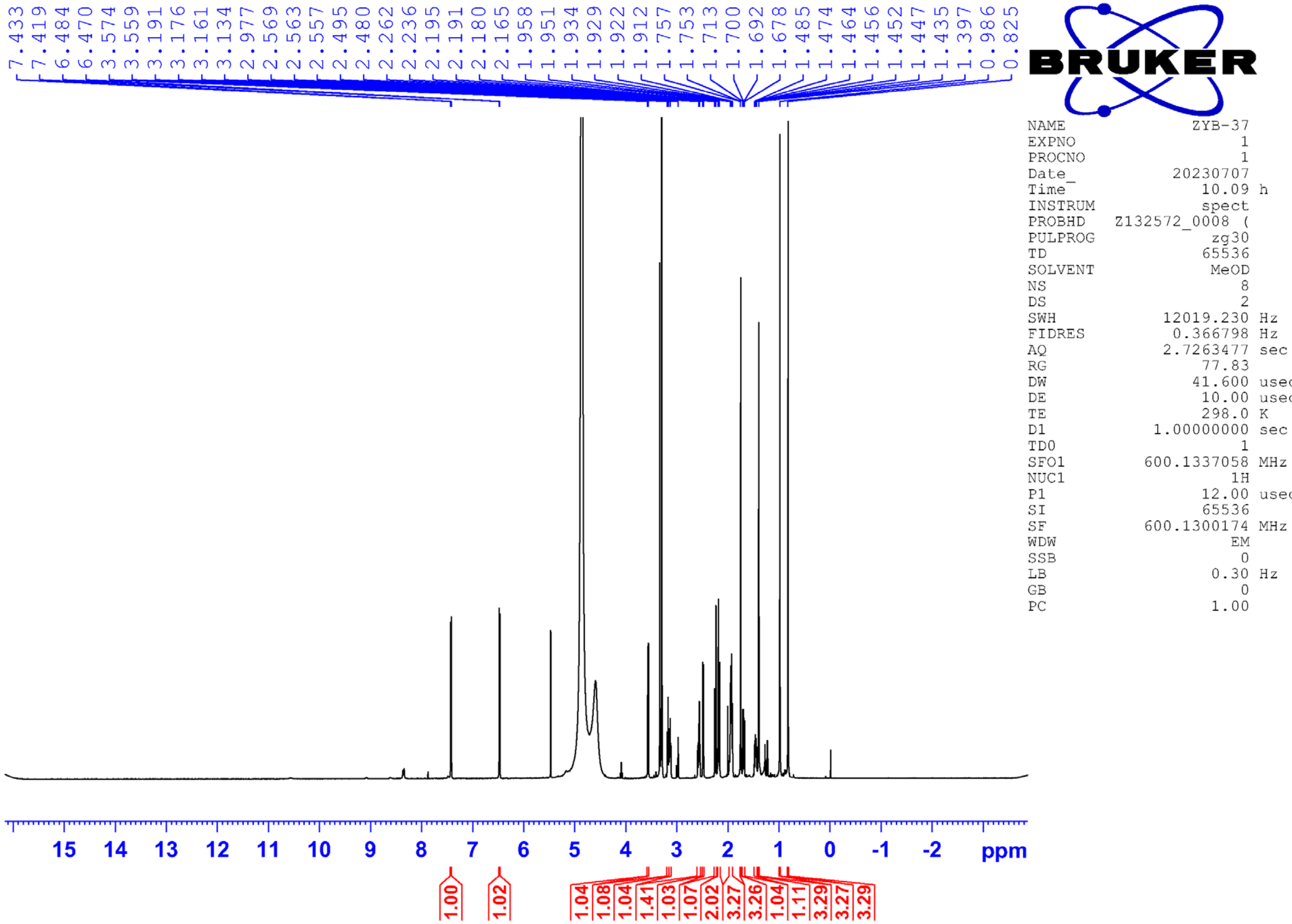

Figure S69. ^{1}H NMR spectrum of **19** in CD_3OD.

Elemental Composition Report

Single Mass Analysis

Tolerance = 5.0 mDa / DBE: min = -1.5, max = 50.0
Element prediction: Off
Number of isotope peaks used for i-FIT = 3

Monoisotopic Mass, Even Electron Ions
1 formula(e) evaluated with 0 results within limits (up to 50 best isotopic matches for each mass)
Elements Used:
C: 0-30 H: 0-60 O: 0-10 23Na: 0-1
ZYB-37 70 (0.290) Cm (68:79)
1: TOF MS ES+

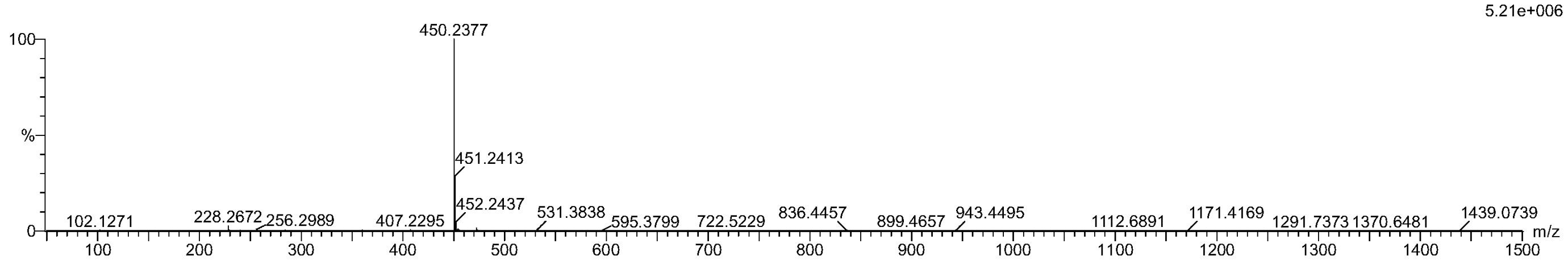

Minimum:				-1.5				
Maximum:		5.0	10.0	50.0				
Mass	Calc. Mass	mDa	PPM	DBE	i-FIT	Norm	Conf(%)	Formula
0.0000	---							

Figure S70. HR-ESI-MS spectrum of **19**.

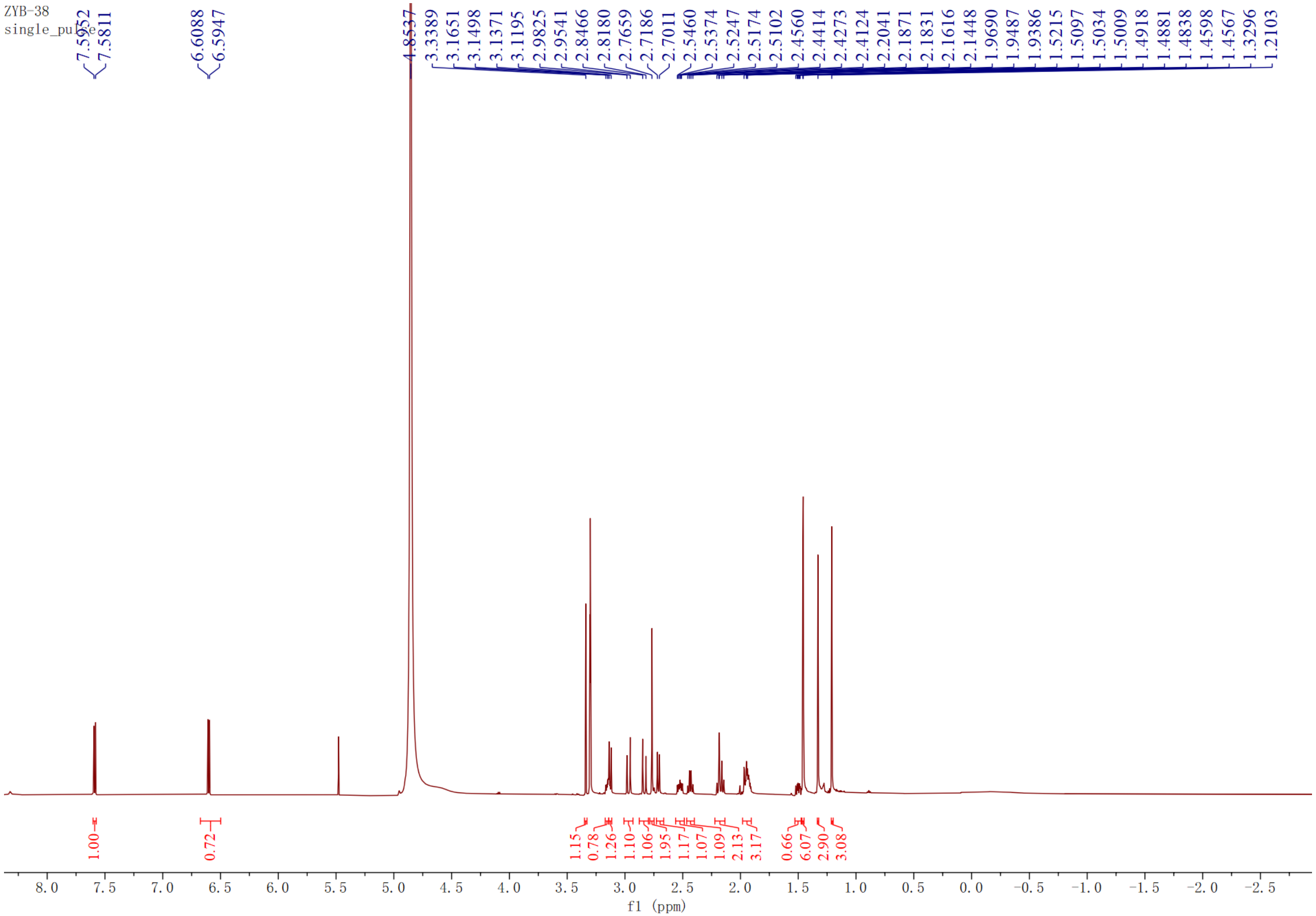

Figure S71. ^{1}H NMR spectrum of **20** in CD_3OD.

Elemental Composition Report **Page 1**

Single Mass Analysis
Tolerance = 5.0 mDa / DBE: min = -1.5, max = 50.0
Element prediction: Off
Number of isotope peaks used for i-FIT = 3

Monoisotopic Mass, Even Electron Ions
1 formula(e) evaluated with 0 results within limits (up to 50 best isotopic matches for each mass)
Elements Used:
C: 0-30 H: 0-60 O: 0-10 23Na: 0-1
ZYB-38 70 (0.290) Cm (70:74)
1: TOF MS ES+

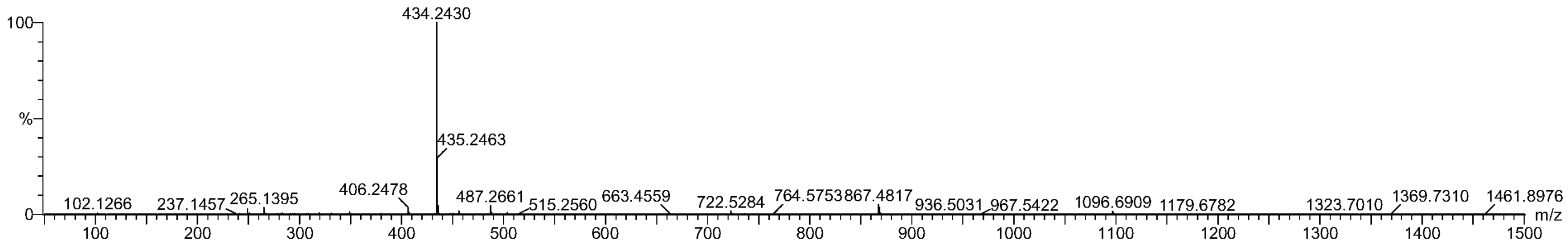

Minimum:				-1.5				
Maximum:		5.0	10.0	50.0				
Mass	Calc. Mass	mDa	PPM	DBE	i-FIT	Norm	Conf(%)	Formula
0.0000	---							

Figure S72. HR-ESI-MS spectrum of **20**.

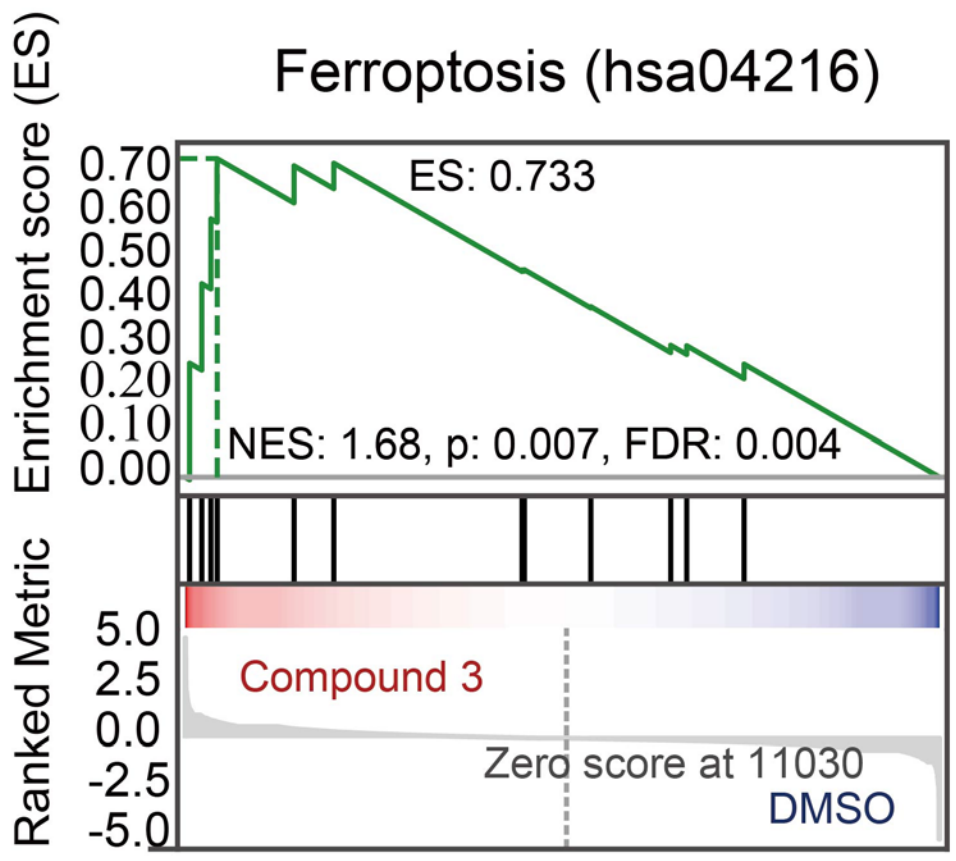

Figure S73. Compund **3** inhibits osteoclastogenesis via ferroptosis GSEA of ferroptosis-related pathways.

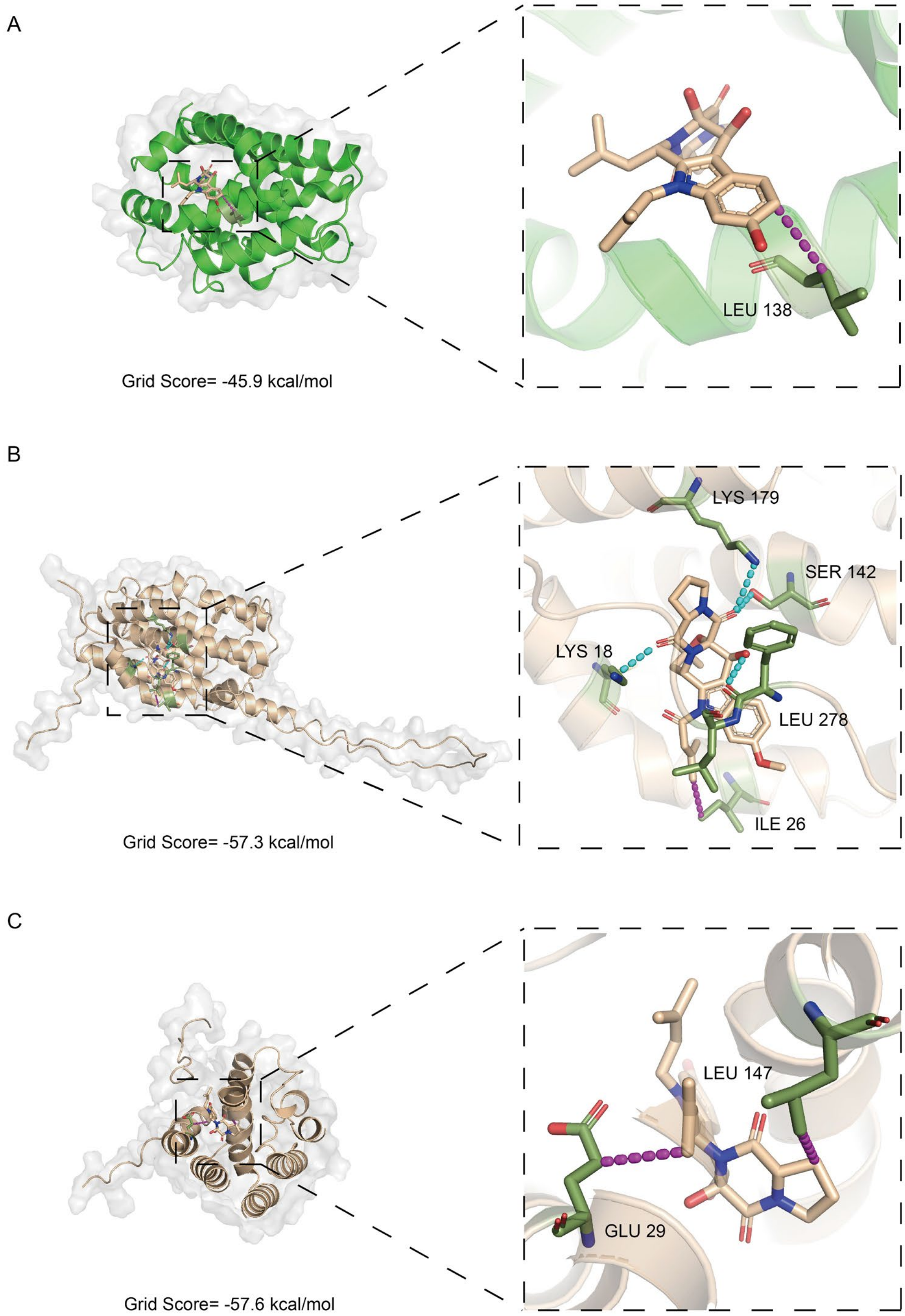

Figure S74. Molecular docking conformation of compounds **3** and **4** bound to HO-1. (**A**) Molecular docking conformation of compound **4** bound to Human HO-1. Key interactions: hydrophobic interaction with leucine 138 (LEU138) (marked with a purple line).(**B**) Molecular docking conformation of compound **3** bound to mouse-derived HO-

1 (**P14901**): Key interactions include hydrogen bonds formed with lysine 18 (LYS 18), leucine 278 (LEU 278), serine 142 (SER 142), and lysine 179 (LYS 179) (marked with cyan lines), as well as a hydrophobic interaction with isoleucine 26 (ILE 26) (marked with a purple line). (**C**) Molecular docking conformation of compound **4** bound to mouse-derived HO-1: Key interactions are the hydrophobic interactions with leucine 147 (LEU 147) and glutamic acid 29 (GLU 29) (marked with purple lines).

.

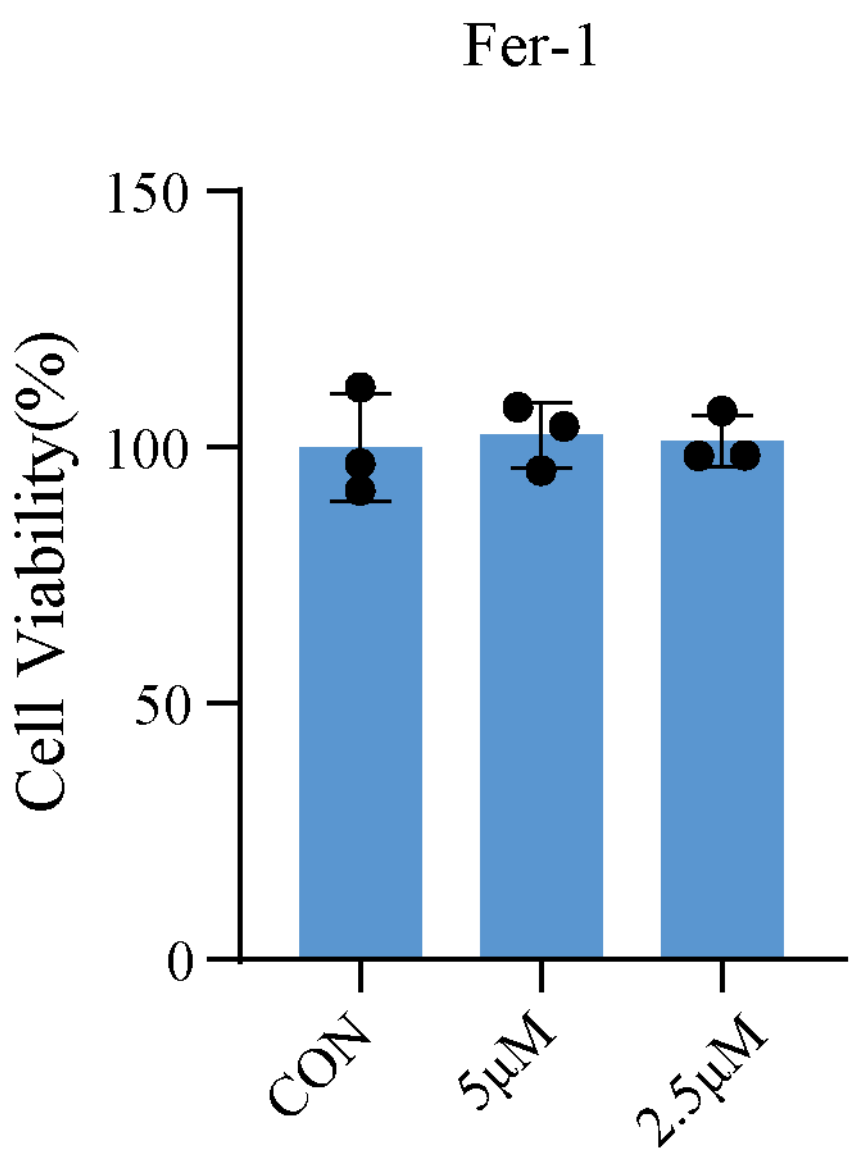

Figure S75. Effect of Fer-1 on BMMs viability measured by the CCK-8 assay. (CON: 0.1%DMSO)

Table S1. Primers used in this work

Primers	Sequences (5' to 3')
Acp5 Mouse-For	GCGACCATTGTTAGCCACATACG
Acp5 Mouse-Rev	CGTTGATGTCGCACAGAGGGAT
Ctsk Mouse-For	AGCAGAACGGAGGCATTGACTC
Ctsk Mouse-Rev	CCCTCTGCATTTAGCTGCCTTTG
Mmp9 Mouse-For	GCTGACTACGATAAGGACGGCA
Mmp9 Mouse-Rev	TAGTGGTGCAGGCAGAGTAGGA
Nfatc1 Mouse-For	GGTGCCTTTTGCGAGCAGTATC
Nfatc1 Mouse-Rev	CGTATGGACCAGAATGTGACGG
Atp6v0d2 Mouse-For	ACGGTGATGTCACAGCAGACGT
Atp6v0d2 Mouse-Rev	CTCTGGATAGAGCCTGCCGCA
Cybb Mouse -For	TGGCGATCTCAGCAAAAGGTGG
Cybb Mouse -Rev	GTACTGTCCCACCTCCATCTTG
Gclm Mouse-For	TCCTGCTGTGTGATGCCACCAG
Gclm Mouse-Rev	GCTTCCTGGAAACTTGCCTCAG
Hmox1 Mouse-For	CACTCTGGAGATGACACCTGAG
Hmox1 Mouse-Rev	GTGTTCCTCTGTCAGCATCACC
Slc7a11 Mouse-For	CTTTGTTGCCCTCTCCTGCTTC
Slc7a11 Mouse-Rev	CAGAGGAGTGTGCTTGTGGACA
Slc40a1 Mouse -For	CCATAGTCTCTGTCAGCCTGCT
Slc40a1 Mouse -Rev	CTTGCAGCAACTGTGTCACCGT

Table S2. Gibbs fee energyand equilibrium populations of low-energy conformers of **1-4** in NMR calculations.

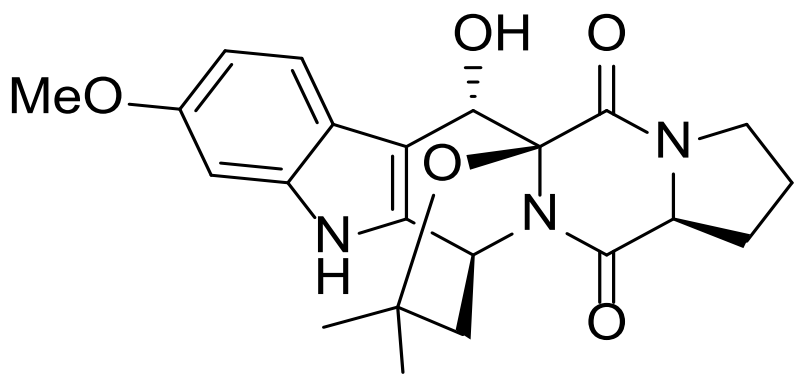

1

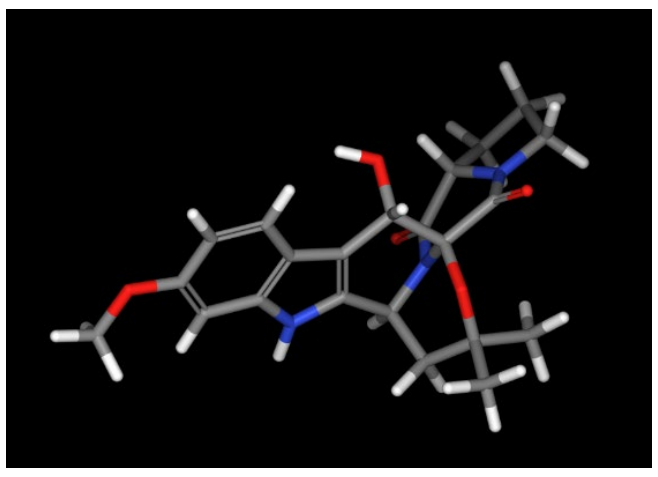

3*S*,6*S*,12*S*,13*S*-**1A**

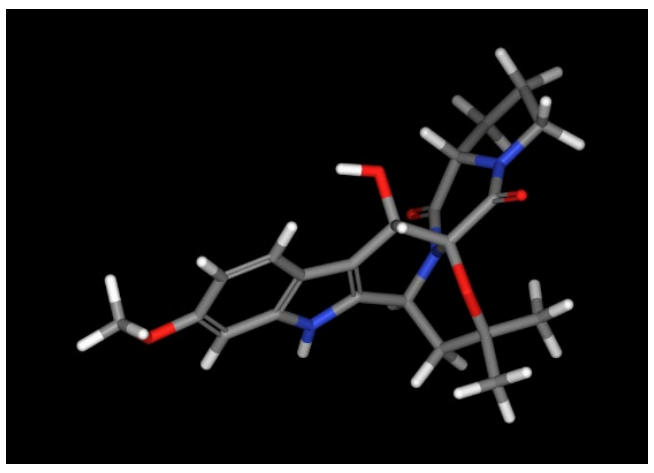

3*S*,6*S*,12*S*,13*S*-**1B**

Conf.	Energy (Hartree)	Energy (kcal/mol)	Population (%)	Theory level	Solvent
3*S*,6*S*,12*S*,13*S*-**1A**	−1393.87415180	−874669.23	75.0	mPW1PW91/6-311G(2d,p)	DMSO
3*S*,6*S*,12*S*,13*S*-**1B**	−1393.87311320	−874668.58	25.0	mPW1PW91/6-311G(2d,p)	DMSO

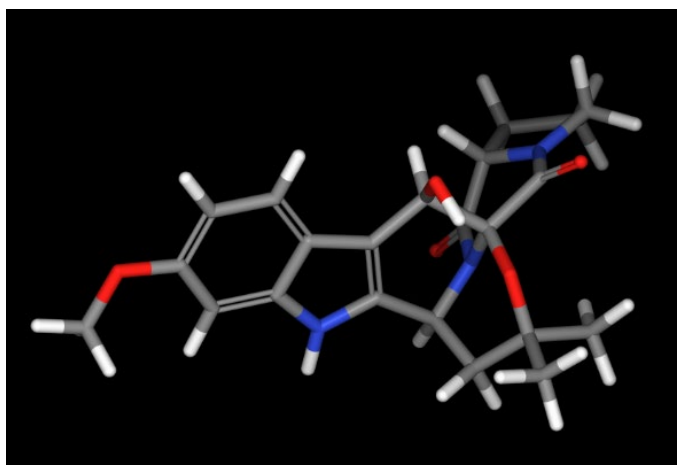

3*S*,6*S*,12*S*,13*R*-**1A**

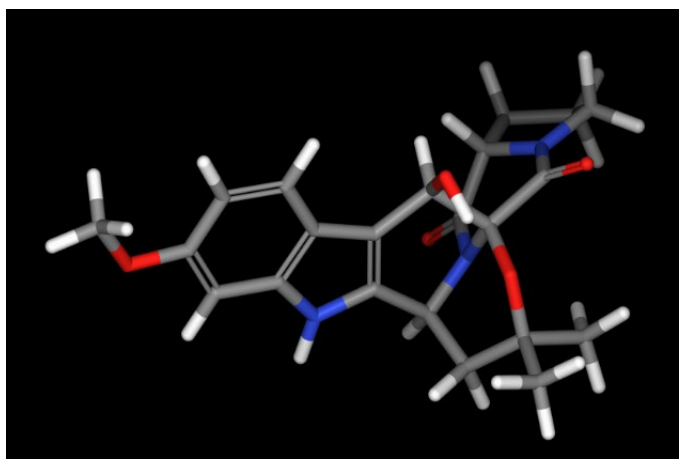

3*S*,6*S*,12*S*,13*R*-**1B**

Conf.	Energy (Hartree)	Energy (kcal/mol)	Population (%)	Theory level	Solvent
3*S*,6*S*,12*S*, 13*R*-**1A**	−1393.87044970	−874666.91	75.3	mPW1PW91/6-311G(2d,p)	DMSO
3*S*,6*S*,12*S*, 13*R*-**1B**	−1393.86939770	−874666.25	24.7	mPW1PW91/6-311G(2d,p)	DMSO

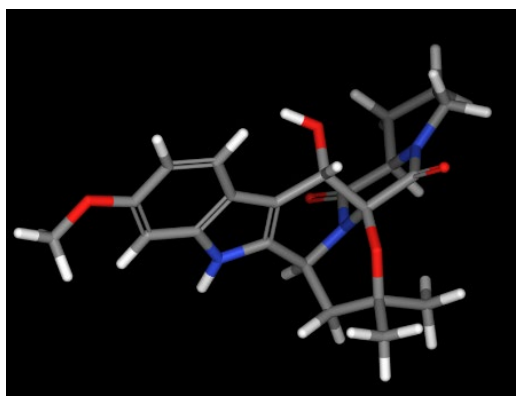 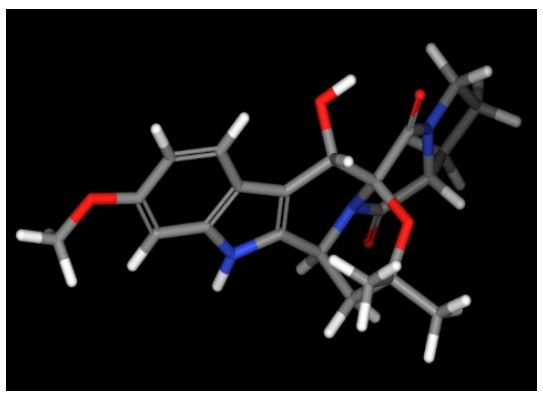 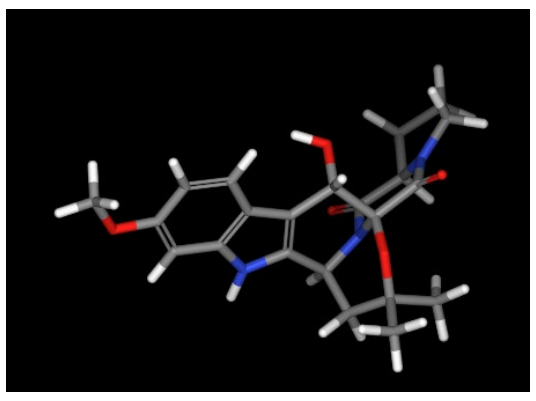 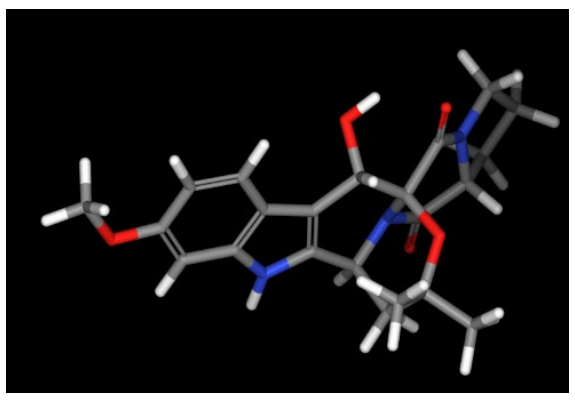

3*S*,6*R*,12*S*,13*S*-**1A** 3*S*,6*R*,12*S*,13*S*-**1B** 3*S*,6*R*,12*S*,13*S*-**1C** 3*S*,6*R*,12*S*,13*S*-**1D**

Conf.	Energy (Hartree)	Energy (kcal/mol)	Population (%)	Theory level	Solvent
3*S*,6*R*,12*S*, 13*S*-**1A**	−1393.87079210	−874667.12	41.6	mPW1PW91/6-311G(2d,p)	DMSO
3*S*,6*R*,12*S*, 13*S*-**1B**	−1393.87058670	−874666.99	33.4	mPW1PW91/6-311G(2d,p)	DMSO
3*S*,6*R*,12*S*, 13*S*-**1C**	−1393.86979030	−874666.49	14.4	mPW1PW91/6-311G(2d,p)	DMSO
3*S*,6*R*,12*S*, 13*S*-**1D**	−1393.86950800	−874666.31	10.6	mPW1PW91/6-311G(2d,p)	DMSO

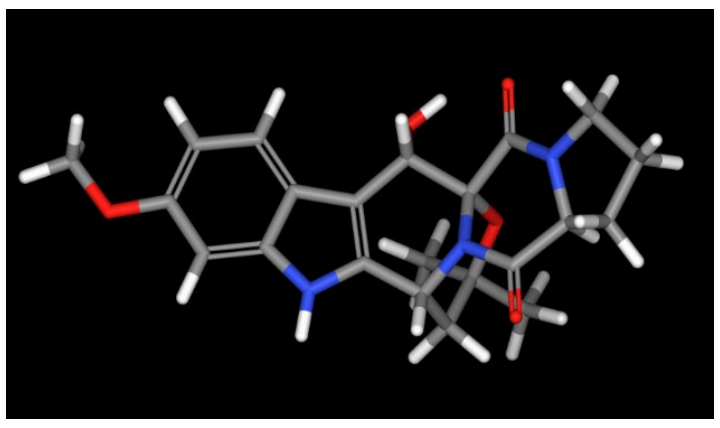

3*R*,6*S*,12*R*,13*S*-**1A**

Conf.	Energy (Hartree)	Energy (kcal/mol)	Population (%)	Theory level	Solvent
3*R*,6*S*,12*R*, 13*S*-**1A**	−1393.86996710	−874666.60	100.0	mPW1PW91/6-311G(2d,p)	DMSO

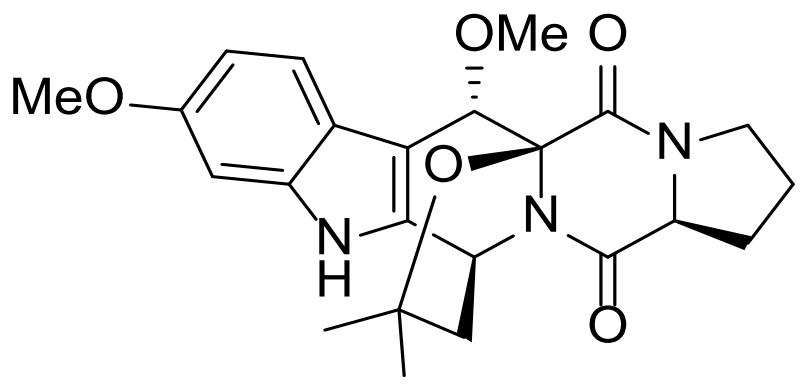

2

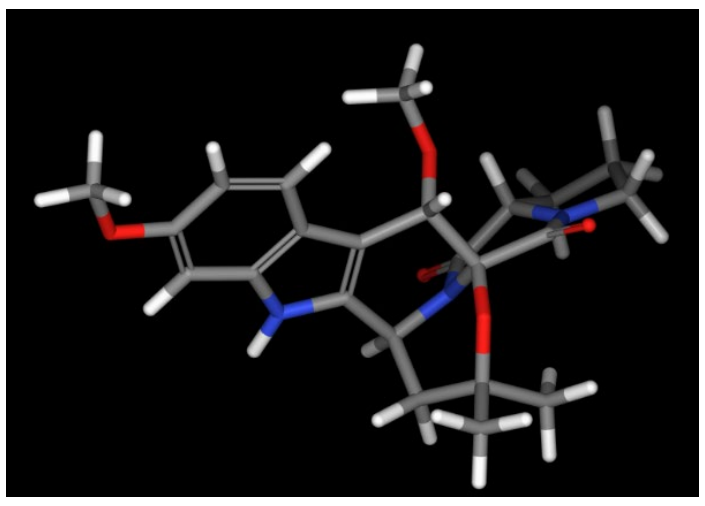

3*S*,6*S*,12*S*,13*S*-**2A**

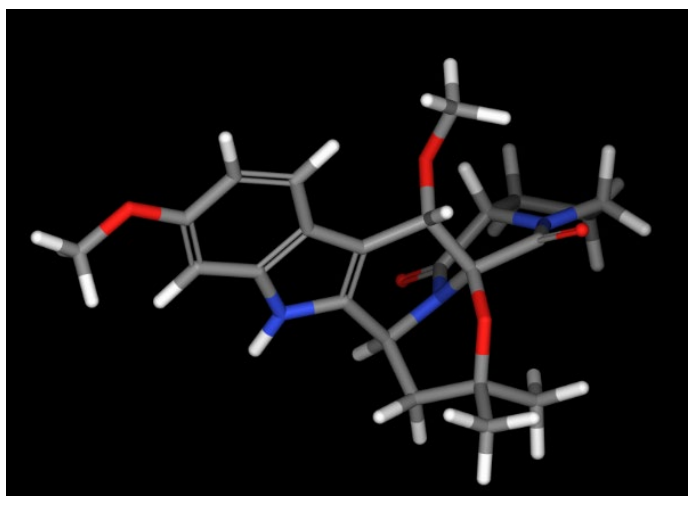

3*S*,6*S*,12*S*,13*S*-**2B**

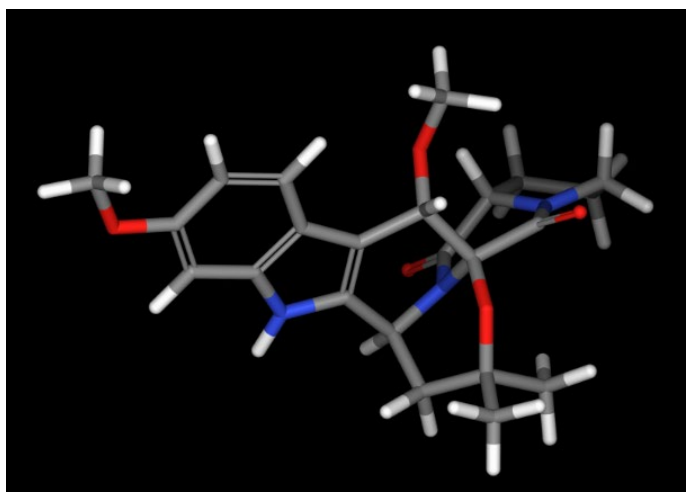

3*S*,6*S*,12*S*,13*S*-**2C**

Conf.	Energy (Hartree)	Energy (kcal/mol)	Population (%)	Theory level	Solvent
3*S*,6*S*,12*S*, 13*S*-**2A**	−1433.16995470	−899327.72	75.7	mPW1PW91/6-311G(2d,p)	DMSO
3*S*,6*S*,12*S*, 13*S*-**2B**	−1433.16861170	−899326.87	18.0	mPW1PW91/6-311G(2d,p)	DMSO
3*S*,6*S*,12*S*, 13*S*-**2C**	−1433.16759420	−874666.49	6.2	mPW1PW91/6-311G(2d,p)	DMSO

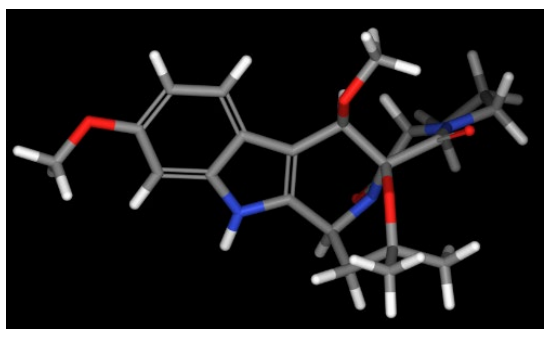

3*S*,6*S*,12*S*,13*R*-**2A**

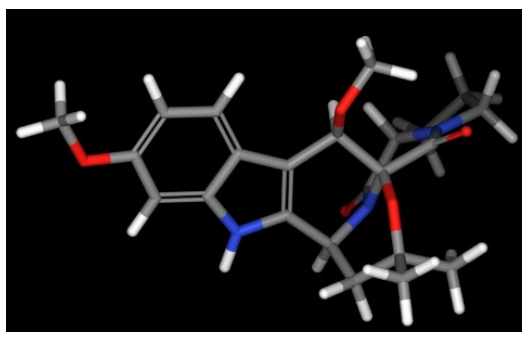

3*S*,6*S*,12*S*,13*R*-**2B**

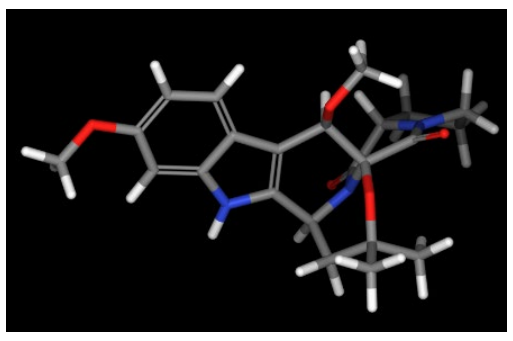

3*S*,6*S*,12*S*,13*R*-**2C**

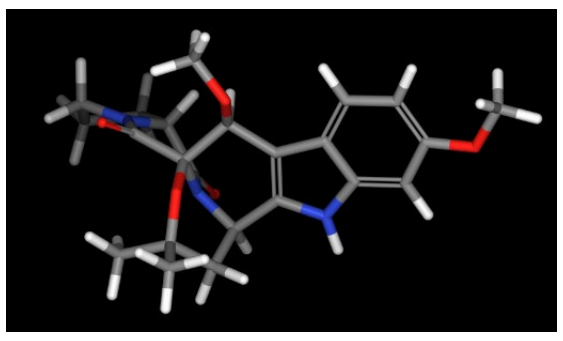

3*S*,6*S*,12*S*,13*R*-**2D**

Conf.	Energy (Hartree)	Energy (kcal/mol)	Population (%)	Theory level	Solvent
3*S*,6*S*,12*S*, 13*R*-**2A**	−1433.16478900	−899324.48	65.3	mPW1PW91/6-311G(2d,p)	DMSO
3*S*,6*S*,12*S*, 13*R*-**2B**	−1433.16370570	−899323.80	20.7	mPW1PW91/6-311G(2d,p)	DMSO
3*S*,6*S*,12*S*, 13*R*-**2C**	−1433.16308060	−899323.40	10.6	mPW1PW91/6-311G(2d,p)	DMSO
3*S*,6*S*,12*S*, 13*R*-**2D**	−1433.16200590	−899322.73	3.4	mPW1PW91/6-311G(2d,p)	DMSO

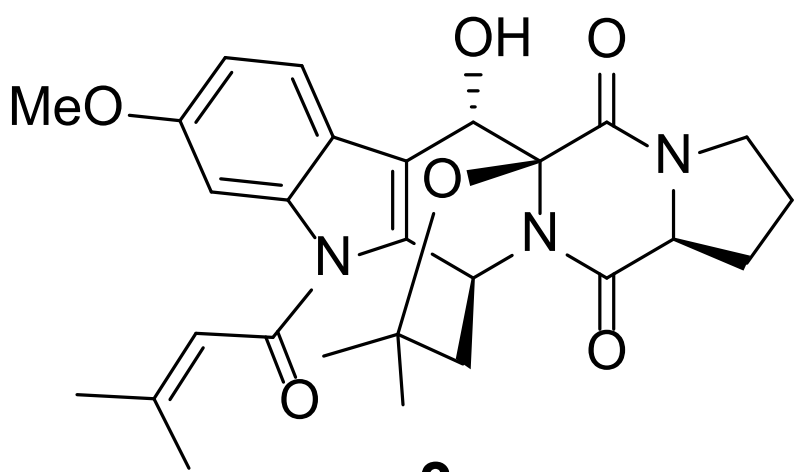

3

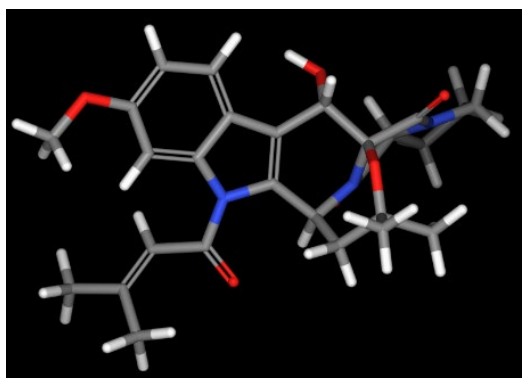

3*S*,6*S*,12*S*,13*S*-**3A**

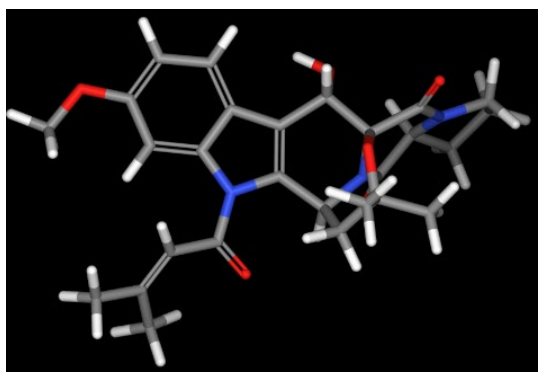

3*S*,6*S*,12*S*,13*S*-**3B**

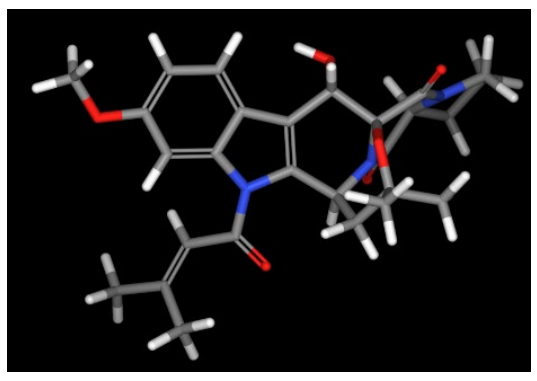

3*S*,6*S*,12*S*,13*S*-**3C**

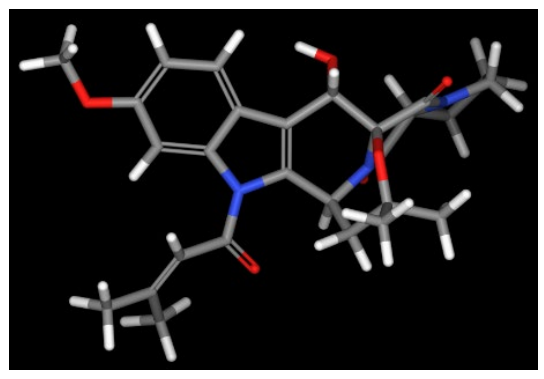

3*S*,6*S*,12*S*,13*S*-**3D**

Conf.	Energy (Hartree)	Energy (kcal/mol)	Population (%)	Theory level	Solvent
3*S*,6*S*,12*S*, 13*S*-**3A**	−1663.24637430	−1043702.85	42.0	mPW1PW91/6-311G(2d,p)	DMSO
3*S*,6*S*,12*S*, 13*S*-**3B**	−1663.24579950	−1043702.49	22.9	mPW1PW91/6-311G(2d,p)	DMSO
3*S*,6*S*,12*S*, 13*S*-**3C**	−1663.24577230	−1043702.47	22.1	mPW1PW91/6-311G(2d,p)	DMSO
3*S*,6*S*,12*S*, 13*S*-**3D**	−1663.24527050	−1043702.16	13.1	mPW1PW91/6-311G(2d,p)	DMSO

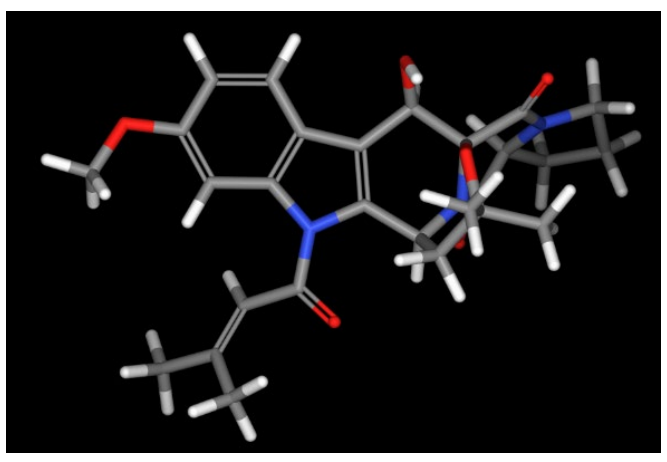

3*S*,6*S*,12*S*,13*R*-**3A**

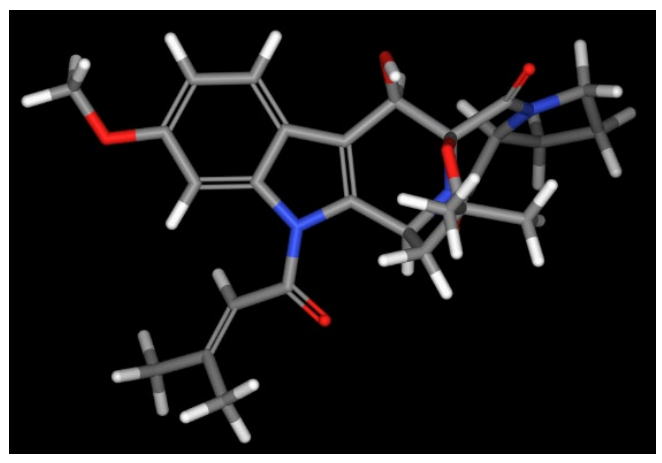

3*S*,6*S*,12*S*,13*R*-**3A**

Conf.	Energy (Hartree)	Energy (kcal/mol)	Population (%)	Theory level	Solvent
3*S*,6*S*,12*S*, 13*R*-**3A**	−1663.24343830	−1043701.01	64.0	mPW1PW91/6-311G(2d,p)	DMSO
3*S*,6*S*,12*S*, 13*R*-**3B**	−1663.24290800	−1043700.67	36.0	mPW1PW91/6-311G(2d,p)	DMSO

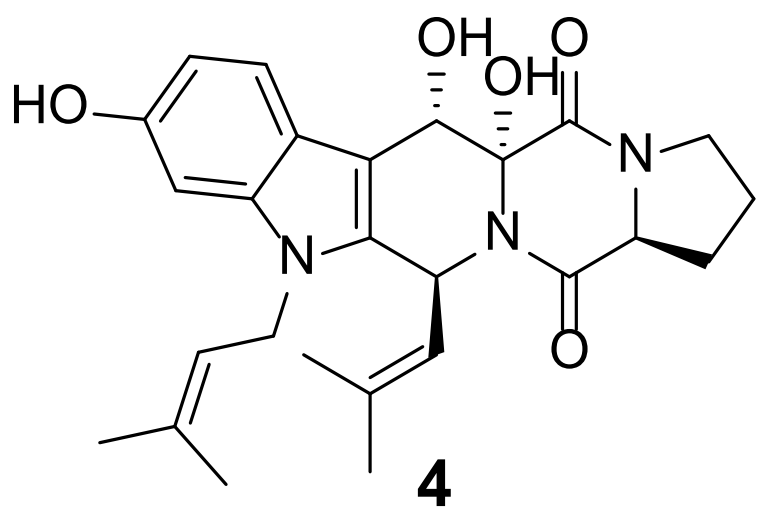

4

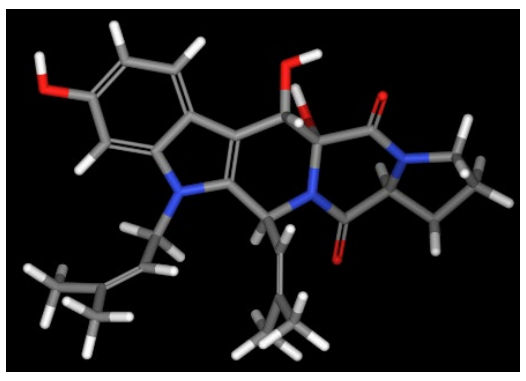

3*S*,6*S*,12*R*,13*S*-**4A**

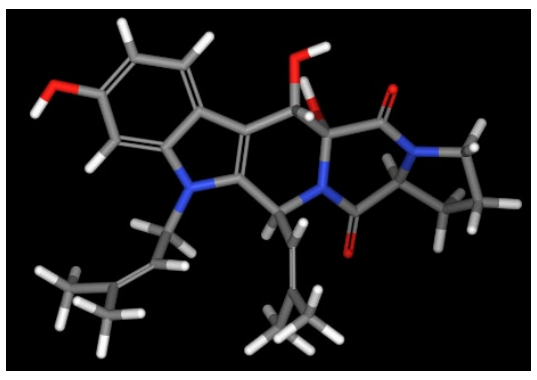

3*S*,6*S*,12*R*,13*S*-**4B**

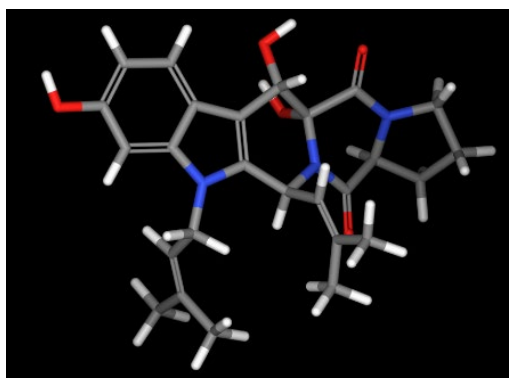

3*S*,6*S*,12*R*,13*S*-**4C**

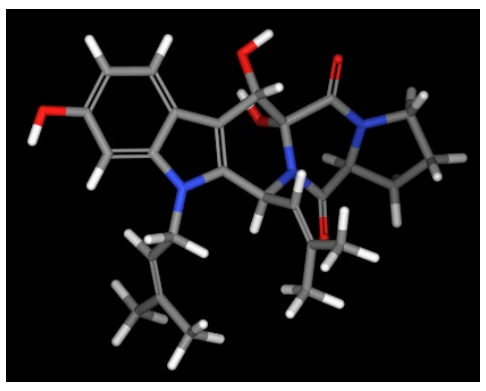

3*S*,6*S*,12*R*,13*S*-**4D**

Conf.	Energy (Hartree)	Energy (kcal/mol)	Population (%)	Theory level	Solvent
3*S*,6*S*,12*R*, 13*S*-**4A**	−1549.90464420	−972579.84	70.7	mPW1PW91/6-311G(2d,p)	MeOH
3*S*,6*S*,12*R*, 13*S*-**4B**	−1549.90325570	−972578.97	16.3	mPW1PW91/6-311G(2d,p)	MeOH
3*S*,6*S*,12*R*, 13*S*-**4C**	−1549.90241510	−972578.44	6.7	mPW1PW91/6-311G(2d,p)	MeOH
3*S*,6*S*,12*R*, 13*S*-**4D**	−1549.90238270	−972578.42	6.4	mPW1PW91/6-311G(2d,p)	MeOH

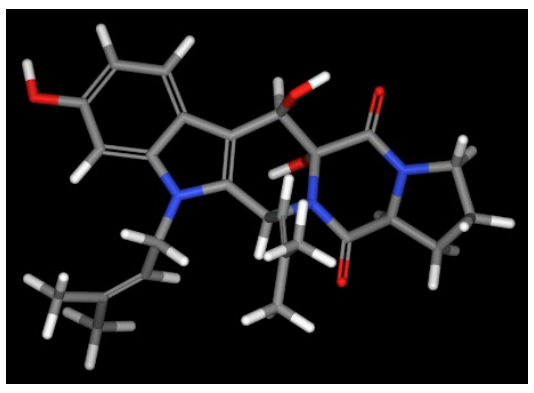

3*S*,6*S*,12*R*,13*R*-**4A**

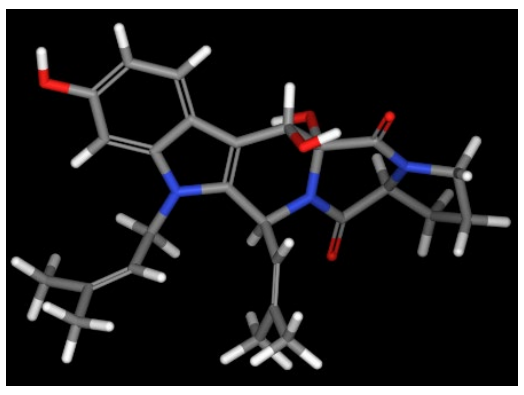

3*S*,6*S*,12*R*,13*R*-**4B**

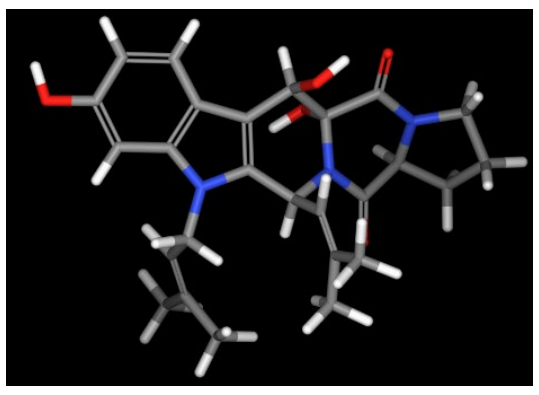

3*S*,6*S*,12*R*,13*R*-**4C**

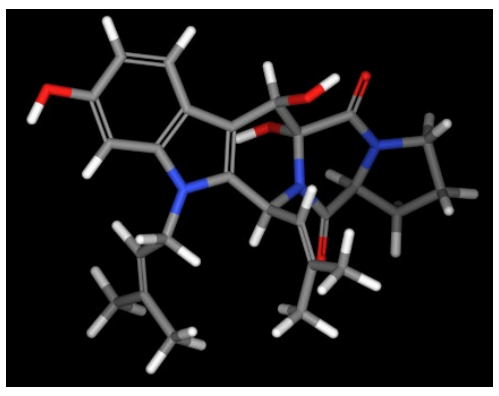

3*S*,6*S*,12*R*,13*R*-**4D**

Conf.	Energy (Hartree)	Energy (kcal/mol)	Population (%)	Theory level	Solvent
3*S*,6*S*,12*R*, 13*R*-**4A**	−1549.90356060	−972579.16	36.7	mPW1PW91/6-311G(2d,p)	MeOH
3*S*,6*S*,12*R*, 13*R*-**4B**	−1549.90353720	−972579.15	36.1	mPW1PW91/6-311G(2d,p)	MeOH
3*S*,6*S*,12*R*, 13*R*-**4C**	−1549.90264080	−972578.58	13.8	mPW1PW91/6-311G(2d,p)	MeOH
3*S*,6*S*,12*R*, 13*R*-**4D**	−1549.90260070	−972578.56	13.3	mPW1PW91/6-311G(2d,p)	MeOH

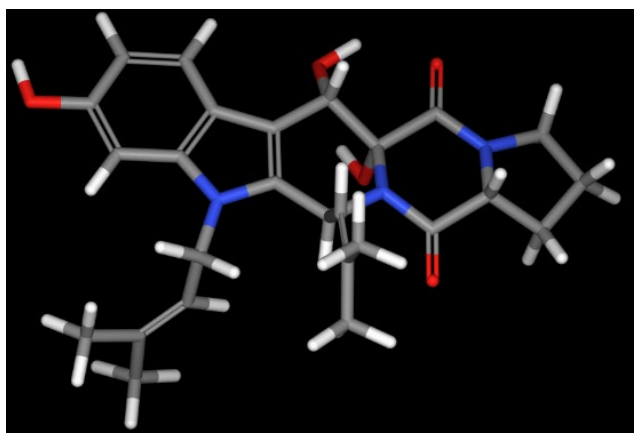

3*S*,6*R*,12*R*,13*S*-**4A**

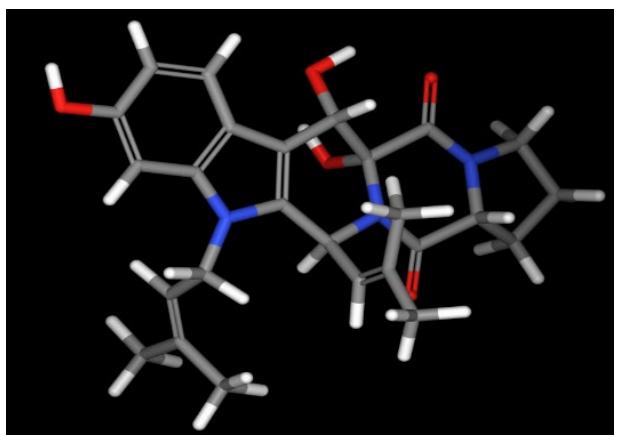

3*S*,6*R*,12*R*,13*S*-**4B**

Conf.	Energy (Hartree)	Energy (kcal/mol)	Population (%)	Theory level	Solvent
3*S*,6*R*,12*R*, 13*S*-**4A**	−1549.89804130	−972575.70	96.3	mPW1PW91/6-311G(2d,p)	MeOH
3*S*,6*R*,12*R*, 13*S*-**4B**	−1549.89495050	−972573.76	3.6	mPW1PW91/6-311G(2d,p)	MeOH

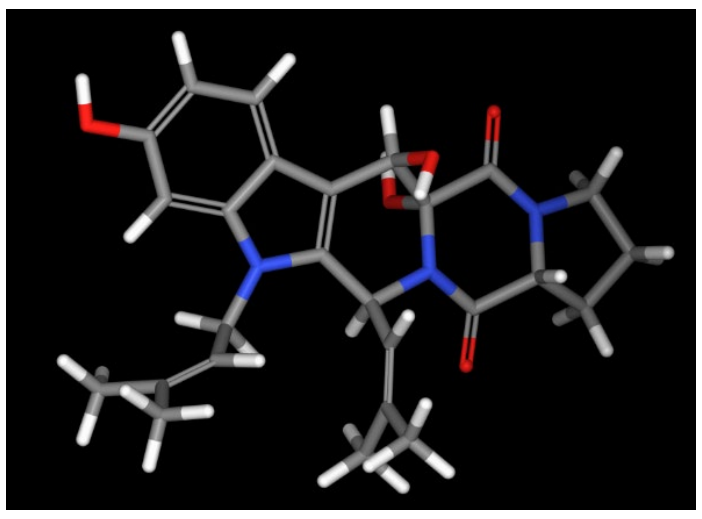

3*S*,6*R*,12*R*,13*R*-**4A**

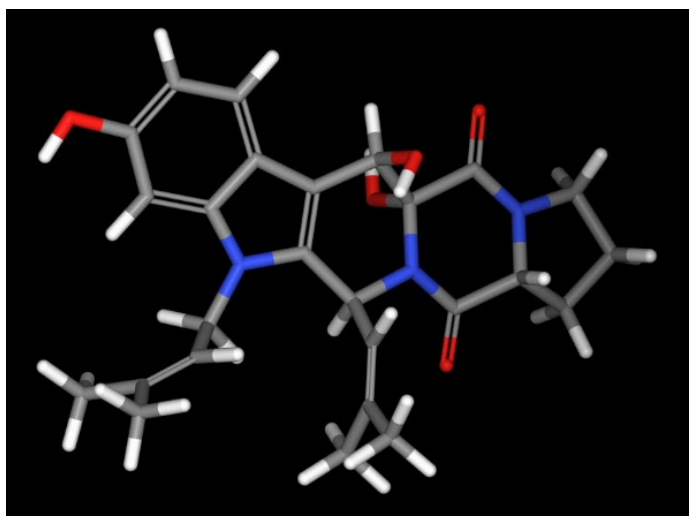

3*S*,6*R*,12*R*,13*R*-**4B**

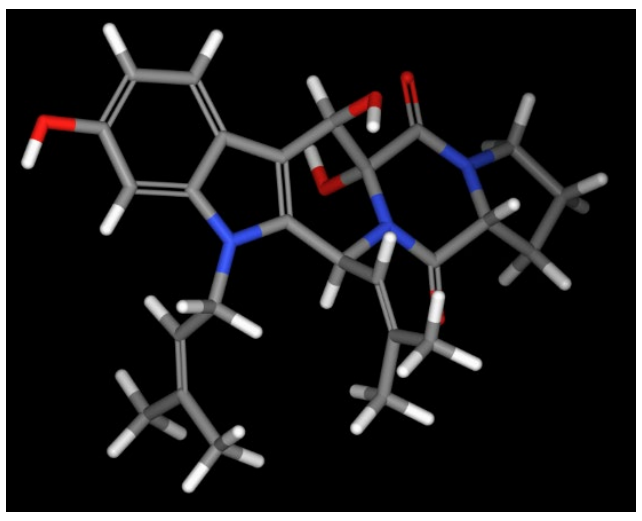

3*S*,6*R*,12*R*,13*R*-**4C**

Conf.	Energy (Hartree)	Energy (kcal/mol)	Population (%)	Theory level	Solvent
3*S*,6*S*,12*R*, 13*R*-**4A**	−1549.89335460	−972572.76	47.2	mPW1PW91/6-311G(2d,p)	MeOH
3*S*,6*S*,12*R*, 13*R*-**4B**	−1549.89326910	−972572.70	42.6	mPW1PW91/6-311G(2d,p)	MeOH
3*S*,6*S*,12*R*, 13*R*-**4C**	−1549.89191350	−972571.85	10.2	mPW1PW91/6-311G(2d,p)	MeOH
